# Design and Synthesis of Fluorescence-Labeled TAK779 Analogs as Chemical Probes

**DOI:** 10.3390/molecules30122655

**Published:** 2025-06-19

**Authors:** Hiroyuki Konno, Takuya Saito, Taichi Aota, Daiki Takanuma, Mizuho Okuyama, Chikako Yokoyama

**Affiliations:** 1 Department of Chemistry and Biological Engineering, Graduate School of Science and Engineering, Yamagata University, Yonezawa 992-8510, Yamagata, Japan; 2 Department of Chemistry and Biological Engineering, Graduate School of Engineering, Osaka Metropolitan University, Sumiyoshi-ku, Osaka 558-8585, Japan

**Keywords:** TAK779, molecular probe, cell penetration

## Abstract

*N,N*-Dimethyl *N*-[4-[[[2-(4-methylphenyl)-6,7-dihydro-5*H*-benzocyclohepten-8-yl]carbonyl]amino]benzyl]tetra-hydro-2*H*-pyran-4-aminium chloride (TAK779) has a potent binding affinity for the chemokine receptor CCR5 and low cytotoxicity; however, their interaction remains unknown. We designed and synthesized four fluorescence-labeled TAK779 analogs as chemical probes. Although the binding properties of the fluorescence-labeled TAK779 analogs for CCR5 could not be determined, it was found that they penetrate the cell membranes and localize to the microtubes of HeLa cells.

## 1. Introduction

*N,N*-Dimethyl *N*-[4-[[[2-(4-methylphenyl)-6,7-dihydro-5*H*-benzocyclohepten-8-yl]carbonyl]amino]benzyl]tetra-hydro-2*H*-pyran-4-aminium chloride (**1**, TAK779) [1,2,3] has a potent binding affinity with an IC_50_ value of 1.4 nM for the chemokine receptor CCR5, a G-protein-coupled seven-transmembrane domain receptor, and low cytotoxicity. TAK779 (**1**) inhibits HIV-1 entry into target cells by blocking the interaction between the gp120/CD4 complex and CCR5 [4,5,6,7,8,9,10]. However, the molecular interactions between TAK779 (**1**) and CCR5 are still unknown, although they have been investigated via molecular modeling [11,12,13,14,15]. To perform high-resolution solid-state NMR spectroscopy studies on this compound, we previously reported the synthesis of 100% enriched [^13^C_3_]-labeled TAK779, which contains ^13^C isotopes at C-19, -35, and -36 [16]. Moreover, the synthesis of the TAK779 derivatives that conjugated the fluorescent materials and its evaluation have never been reported, and TAK779 (**1**) has failed in clinical trials. Although it can penetrate cells, its selective interaction with CCR5 is difficult to achieve. Cell penetration of bioactive and biofunctional compounds is essential for their interaction with cell organelles. However, compounds with cell-penetrating behavior frequently show potent cytotoxicity; therefore, the development of nontoxic compounds with cell penetrating behavior is an important yet challenging task. Other CCR5 inhibitors, like the callipletin family [17,18] and anibamine [19,20,21] as natural products and SCH-D [22,23] and cenicriviroc [24,25] as synthetic compounds, also suffer from cytotoxicity. Moreover, maraviroc is a CCR5 inhibitor only used in the treatment of HIV [26,27]. As a result of our investigation on the modification of TAK779 (**1**) to control its behavior as a cell-penetrating bioprobe, we herein report the design, synthesis, and behavior as chemical probes of fluorescence-labeled TAK779 analog (**2**).

## 2. Results and Discussion

**Figure 1:**
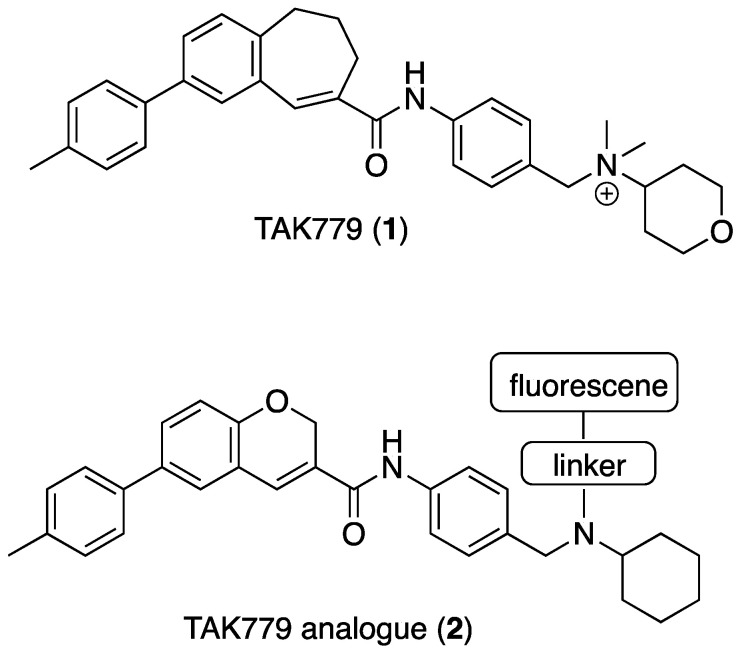
Chemical structure of TAK779 (**1**) and its analogs (**2**) as chemical probes.

In the molecular design for a CCR5-binding fluorescence probe, TAK779 (**1**) was used as a model compound. To decrease its interaction with CCR5, the seven-membered ring of TAK779 (**1**) was replaced with a cyclopyran ring, and the amine salt was bound to a fluorescence material through a linker to give TAK779 analogs (**2**). The IC_50_ value of the TAK779 analog with the cyclopyran ring is reduced 26-fold compared with those with the cycloheptane ring from the structure activity relationship study. In addition, when the cyclohexane ring of TAK779 analog was replaced with the cyclopyran ring, its IC_50_ value was decreased 3-fold [1,2,3]. We estimated that **2** exhibited 100 times less affinity for CCR5 than TAK779 (**1**) (Figure 1).

**Scheme 1:**
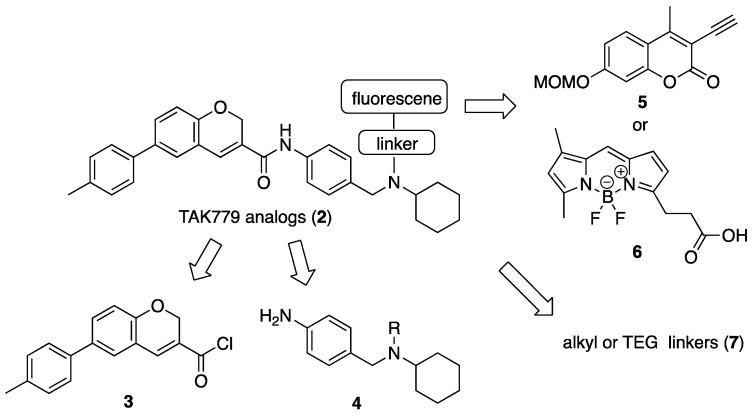
Retrosynthetic analysis of TAK779 analog-based chemical probes (**2**).

Scheme 1 shows the retrosynthetic analysis for TAK779 analogs (**2**), which were synthesized from phenylpyran (**3**) [28,29] and amine (**4**) [30]. A hydrophobic C11-alkyl linker was prepared to bind the TAK779 analog and coumarin (**5**) [31,32,33]. Amide bond formation between the TAK779 analog and the C11-alkyl linker and Huisgen coupling reaction between coumarin (**5**) and the C11-alkyl linker were performed to obtain **2a**. Meanwhile, **2b** bearing glutaramide and triethylene glycol (TEG) was designed to exhibit increased hydrophilicity. Moreover, the BODIPY derivative (**6**) [34] was used as a fluorescent material to prepare **2c**. TAK779 analogs bearing the TEG linker and the BODIPY derivative (**6**) were coupled via amide bond formations (Scheme 1). The preparation of four segments (**3**–**6**) was performed as previously reported [28,29,30,31,32,33,34] (see Supporting Information).

**Scheme 2:**
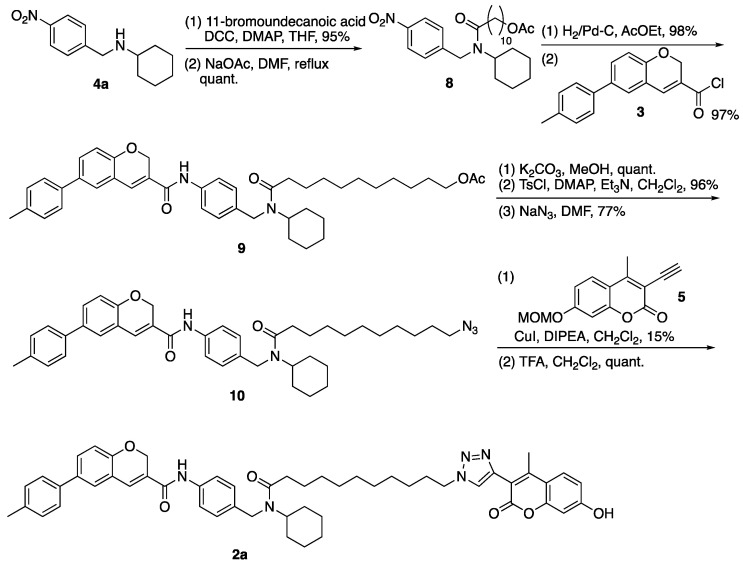
Synthesis of the coumarin-labeled TAK779 analog (**2a**).

Coumarin-labeled TAK779 analog (**2a**) was synthesized as shown in Scheme 2. Coupling of amine (**4a**) and 12-bromoundecanoic acid in the presence of DCC/DMAP in THF gave the corresponding amide derivative (**4c**) in 95% yield. Subsequent treatment with NaOAc in DMF reflux afforded **8** in quantitative yield. After reducing the nitro group of **8** (98% yield), coupling with phenylpyran (**3**) gave the TAK779 analog framework (**9**) in 97% yield. Azide (**10**) was obtained in 74% yield by subjecting compound **9** to the following three-step sequence: deprotection of the acetyl group, tosylation of the corresponding hydroxy group, and azidation with NaN_3_. Then, the click reaction [35,36,37,38,39] between azide (**10**) acetylene-containing coumarin (**5**) in the presence of CuI/DIPEA in CH_2_Cl_2_ afforded the desired coupled triazole compound (**10a**) in 15% yield. Finally, TFA-mediated deprotection of the MOM group smoothly proceeded to give the coumarin-labeled TAK779 analog (**2a**) in quantitative yield (Scheme 2).

**Scheme 3:**
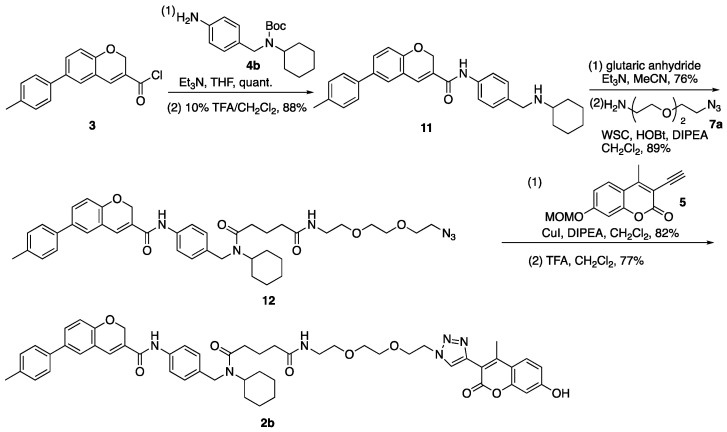
Synthesis of the coumarin-labeled TAK779 analog (**2b**).

Coumarin-labeled TAK779 analog (**2b**) was synthesized as shown in Scheme 3. After the coupling between **3** and **4b** quantitatively, deprotection of the Boc group in 10% TFA/CH_2_Cl_2_ afforded TAK779 analog (**11**) in 88% yield. To install the hydrophobic spacer, glutaric acid and TEG were selected. The coupling efficiency of the secondary amino group of TAK779 analog (**11**) and long alkyl and TEG spacers was frequently lower. Therefore, the cyclic anhydride compound, glutaric acid, was used. Then, the TEG unit was introduced via amide bond formation. The coupling of **11** and glutaric anhydride proceeded in 76% yield, and the coupling of the resulting compound (**11a**) with TEG (**7a**) in the presence of WSC/HOBt•H_2_O/DIPEA gave the azide-containing TAK779 analog (**12**) in 89% yield. The click reaction between the azide on the TAK779 analog (**12**) and the acetylene on the coumarin analog (**5**) in CuI/DIPEA in CH_2_Cl_2_ smoothly proceeded to give the TAK779 analog (**12a**), bearing a triazole unit in 82% yield. After deprotection of the MOM group in 50% TFA/CH_2_Cl_2_, the coumarin-labeled TAK779 analog (**2b**) was obtained in 77% yield (Scheme 3).

**Scheme 4:**
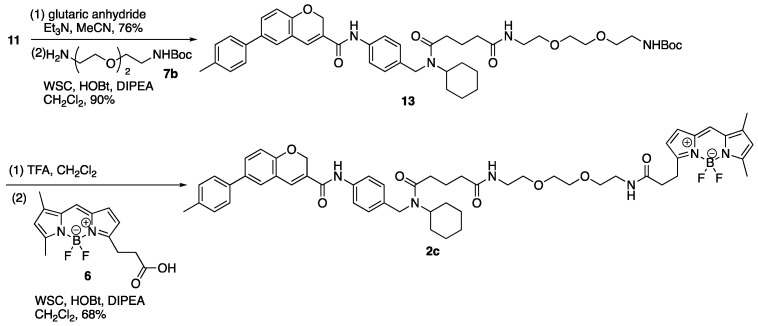
Synthesis of BODIPY-labeled TAK779 analog (**2c**).

To compare the effect of using difference fluorescent materials, BODIPY-FL (**6**) was bound to the TAK779 analog (**11**). Treatment of the TAK779 analog (**11**) and glutaric anhydride in the presence of Et_3_N resulted in the formation of an amide bond, affording the corresponding carboxylic acid (**11a**) in 76% yield. Elongation of the spacer unit using TEG derivative (**7b**) yielded the Boc compound (**13**) in 90% yield. Subsequently, deprotection of the Boc group of **13** in 10% TFA/CH_2_Cl_2_ and coupling of the corresponding amino group with the carboxylic acid of BODIPY-FL (**6**) in WSC/HOBt•H_2_O/DIPEA in CH_2_Cl_2_ gave the BODIPY-labeled TAK779 analog (**2c**) in 68% yield (Scheme 4).

**Scheme 5:**
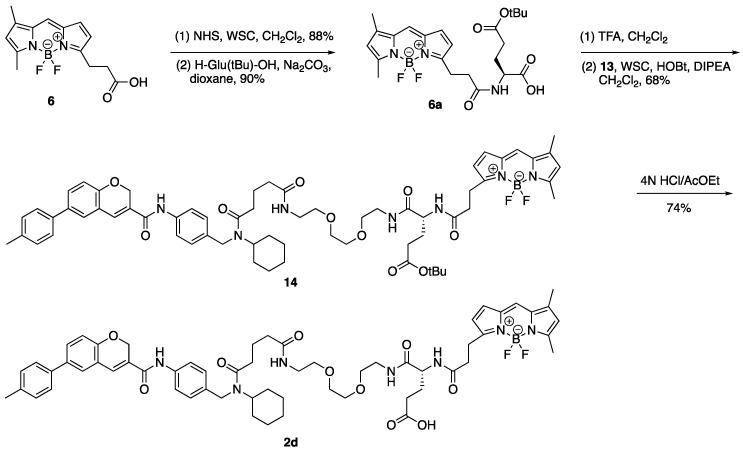
Synthesis of BODIPY-labeled Glu TAK779 analog (**2d**).

To increase the aqueous solubility of the TAK779 analogs (**2**), BODIPY-labeled Glu TAK779 analogue (**2d**) was prepared by introducing a glutamic acid residue between TEG spacer and BODIPY-FL in **2c** as shown in Scheme 5. Treatment of BODIPY-FL (**6**) and *N*-hydroxysuccinimide (NHS) with WSC in CH_2_Cl_2_ afforded the corresponding activated ester derivative (**6b**), which was coupled with H-Glu(tBu)-OH in the presence of Na_2_CO_3_-furnished BODIPY-Glu derivative (**6a**) in 90% yield. After the deprotection of the Boc group of **13** in 10% TFA/CH_2_Cl_2_, coupling with the BODIPY-Glu derivative (**6a**) gave the TAK779 derivative (**14**) in 68% yield. Finally, deprotection of the *t*-butyl group of the side chain of the Glu residue in **14** using 4 N HCl/AcOEt gave BODIPY-labeled Glu TAK779 analog (**2d**) in 74% yield (Scheme 5).

**Scheme 6:**
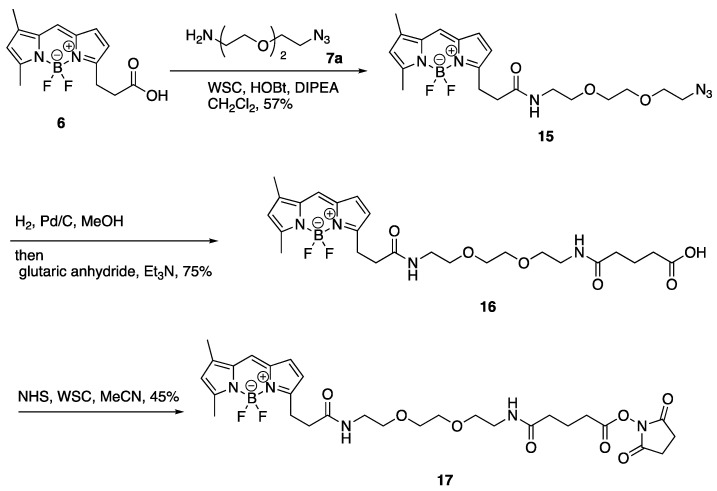
Preparation of BODIPY amide (**17**).

Next, reference compounds **15**, **16**, and **17** were prepared (Scheme 6). BODIPY-containing propionic acid (**6**) and TEG linker amine (**7a**) were coupled using WSC/HOBt•H_2_O/DIPEA in CH_2_Cl_2_ to afford BODIPY azide (**15**) in 57% yield. Hydrogenolysis of **15** in the presence of Pd/C in a hydrogen atmosphere gave the corresponding amine, and the subsequent addition of glutaric anhydride and Et_3_N to the reaction mixture afforded the BODIPY carboxylic acid (**16**) in 75% yield in a one-pot manner. BODIPY amide (**17**) was obtained by coupling of BODIPY carboxylic acid (**16**) and Et_2_NH in 54% yield (Scheme 6). All synthesized compounds were assigned by ^1^H and ^13^C-NMR, IR, and high-resolution mass spectra. And these collected data were reasonable for the resigned structures (see experimental and Supporting Information).

**Figure 2:**
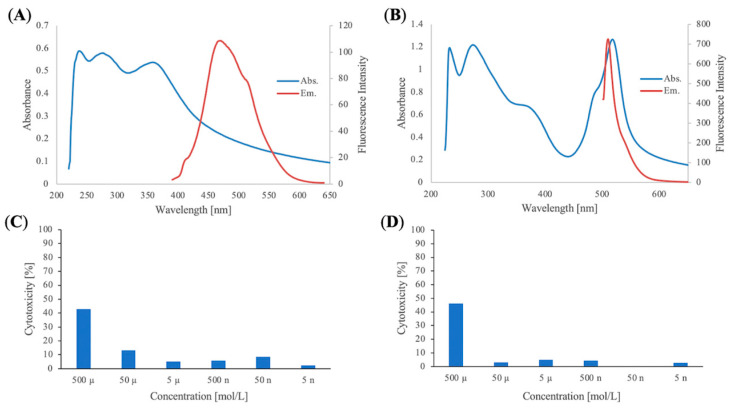
Fluorescence spectra and cytotoxicity of synthetic TAK779 analogs (**2b**, **2c**). (**A**) Fluorescence spectra of **2b**, (**B**) fluorescence spectra of **2c**, (**C**) cytotoxicity of **2b**, and (**D**) cytotoxicity of **2c**.

Under excitation with 320 nm light, the fluorescent spectrum of 50 μM coumarin (**5**) in 1% DMSO showed an emission band at 445 nm [40]. Meanwhile, 50 μM coumarin-labeled TAK779 analog (**2b**) in 1% DMSO produced an emission band at 468 nm upon excitation with 358 nm light. Moreover, the fluorescence spectrum of 50 μM BODIPY (**6**) in 1% DMSO was reported to exhibit an emission band at 515 nm under excitation with 503 nm light [41]. In our study, the fluorescence spectrum of 50 μM BODIPY-labeled TAK779 analog (**2c**) in 1% DMSO showed an emission band at 510 nm when excited with 495 nm light. In contrast, the coumarin-labeled TAK779 analog (**2a**) exhibited aggregate behavior in 1% DMSO solution, hindering a fluorescent spectrum. This suggested that the alkyl chain spacer of **2a** was not suitable to prepare TAK779 analogs as chemical probes and the TEG unit has considerable hydrophobicity. The clinical potential of an antiviral agent is greatly influenced by its selectivity for target cells [42,43]. Accordingly, the cytotoxicity of **2b** and **2c** was ascertained by studying their effect on the viability of K562 cell lines. No cytotoxicity was observed for either of the compounds (Figure 2).

**Figure 3:**
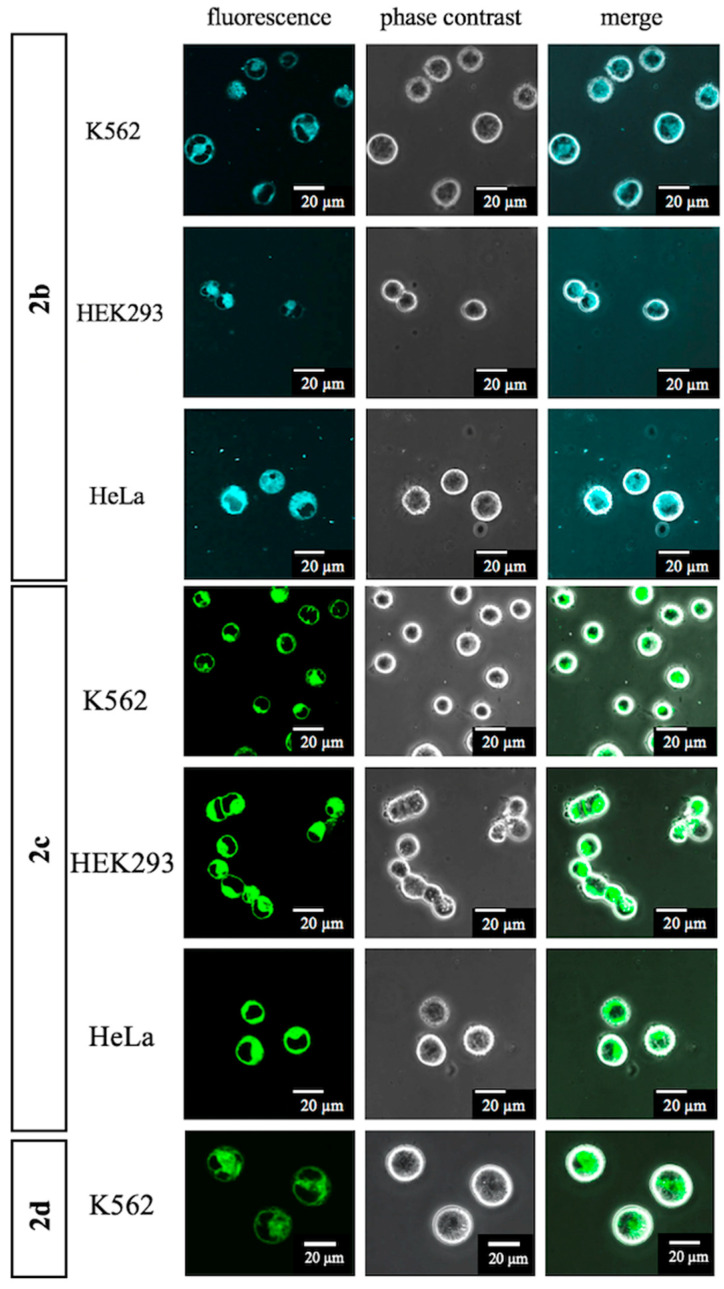
Cell images of K562, HEK293, and HeLa cell lines with coumarin-labeled TAK779 analog (**2b**) (concentration: 50 mM, excitation wavelength: 405 nm, laser intensity: 100%, detection wavelength region: 420–520 nm) and BODIPY-labeled TAK779 analog (**2c**) (concentration: 50 mM, excitation wavelength: 475 nm, laser intensity: 3%, detection wavelength region: 490–540 nm). The assay using flesh-survived cells was performed in floating conditions.

The binding property for CCR5 and the cell penetration behavior of TAK779 analogs (**2b**–**d**) were investigated. K562 cell lines bound to CCR5 on cell membrane, and HEK293 and HeLa cell lines in the absent of CCR5 proteins were selected. The results of the treatment of **2b** and **2c** with the K562, HEK293, and HeLa cell lines are depicted in Figure 3. In the emission wavelength region of 420–450 nm, blue fluorescence emissions derived from the coumarin moiety of **2b** were observed in the cells under excitation with 405 nm light. Similarly, the cells showed green fluorescence emissions derived from the BODIPY moiety of **2c** in the emission wavelength region of 490–540 nm upon excitation with 475 nm light. TAK779 analogs (**2b**,**c**) were localized in each cell line. Moreover, after treatment of K562 cells with TAK779 analog (**2d**) for 1 h, TAK779 analog (**2d**) was also localized in K562 cells without staying on the CCR5 on cell membrane. This suggests that **2b**,**d** have no CCR5 binding property and hydrophilicity, even though they exhibit cell-penetrating and localization properties. Moreover, comparison of the CCR5-expressing HeLa cells may provide useful information (Figure 3).

**Figure 4:**
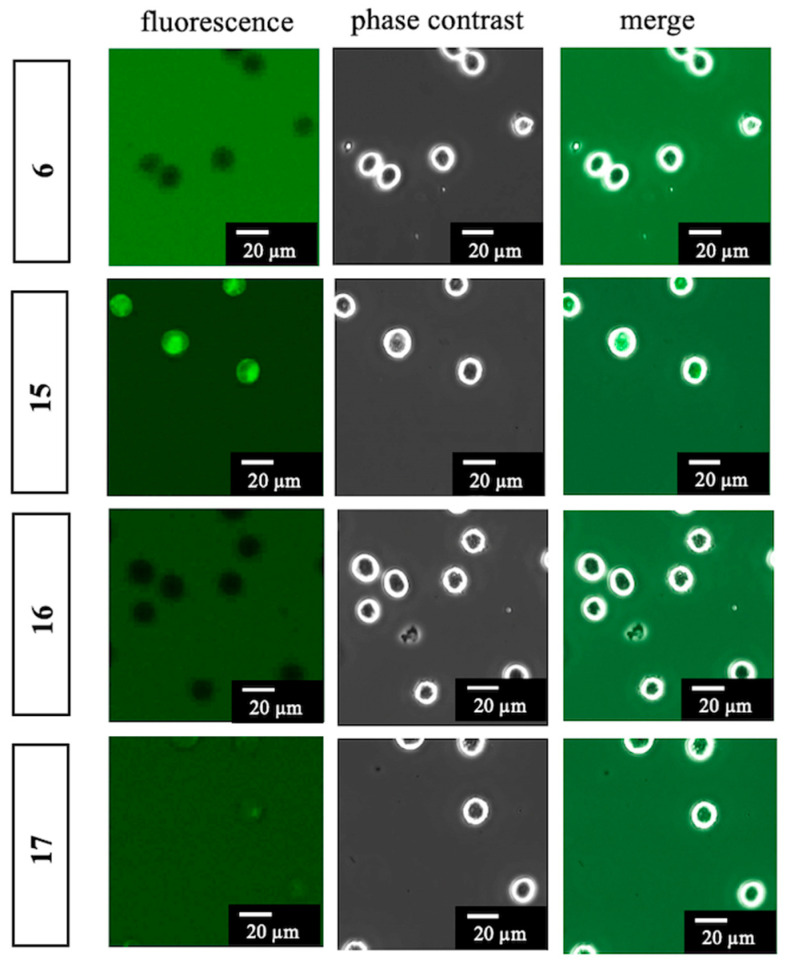
Cell images of K562 cell lines with BODIPY derivatives (**6**, **15**, **16**, and **17**) as control experiments. Concentration: 50 mM, excitation wavelength: 473 nm, laser intensity: 3%, detection wavelength region: 490–540 nm. The assay using flesh-survived cells was performed in floating conditions.

Control experiments revealed that green fluorescence emissions were not observed upon treatment of K562 cell lines with BODIPY carboxylic acid derivatives (**6**) and (**16**), indicating that **6** and **16** rarely penetrate the K562 cell membrane. In contrast, BODIPY derivatives bound with azide (**15**) or amide (**17**) gave the green fluorescence emissions against K562 cell lines. The fluorescence emissions derived from **15** were not localized and were observed in the cell matrix without exception, and those of **17** were quite weak. These results suggested that physical properties with the hydrophobicity and functionality were important and that the BODIPY moiety did not control cell penetration (Figure 4).

**Figure 5:**
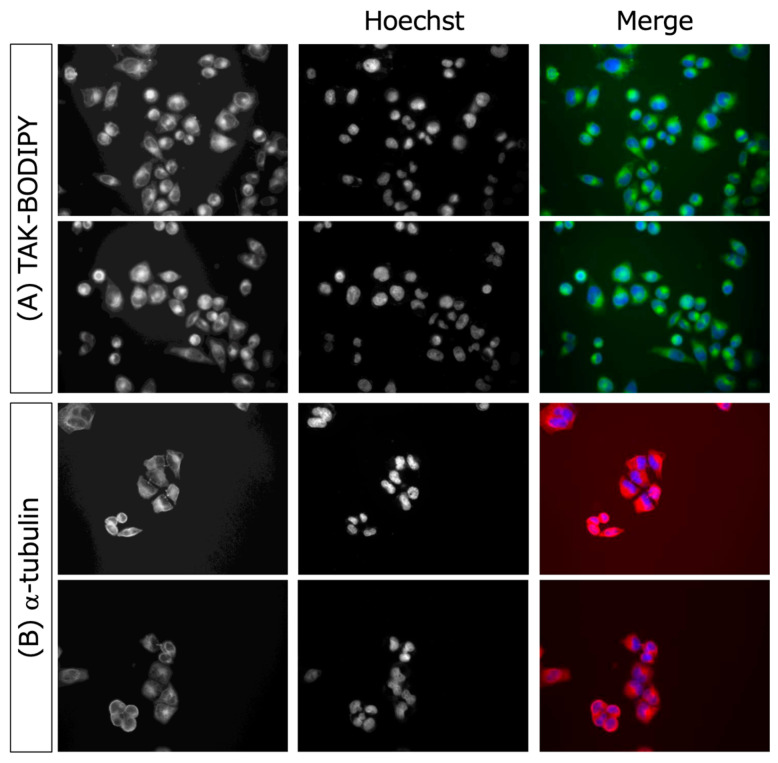
(**A**) Effects of BODIPY-labeled TAK779 analog (**2c**; green) on the microtubules of HeLa cells after 1 h of treatment. (**B**) Effects of α-tubulin antibody on the microtubules of HeLa cells after 1 h of treatment. Localization of antigens was observed using an Alexa568-conjugated goat anti-mouse IgG (1:500 dilution; red). DNA (blue) was stained with Hoechst 33258. Scale bars; 20 μm. The assay using flesh survived cells was performed in floating conditions.

Because the labeled TAK779 analogs (**2b**–**d**) have cell penetration and localization properties, the localization site of HeLa cells was investigated using BODIPY-labeled TAK779 analog (**2c**) and treated on HeLa cells. As depicted in Figure 5, **2c** was nearly localized to the microtubules of HeLa cells, suggesting that **2c** rarely localized to the DNA nuclear site from the Hoechst staining. Additionally, clear effects of the α-tubulin antibody on the microtubules of HeLa cells were observed. It is guessed that the intracellular distribution is not influenced by aggregation of **2c** because cytotoxicity of **2c** is extremely weak (Figure 5).

The interaction between microtubules and low-molecular-weight natural products such as taxol [44], vinblastine [45], and colchicine [46], among others [47], has been investigated for a long time because it causes inhibition of polymerization, depolymerization, or degradation of microtubules, and therefore affects the cell cycles for the application as anticancer agents. In this study, the mechanism of action and localized position are most likely different because the BODIPY-labeled TAK779 analog (**2c**) shows no cytotoxicity. Investigation of a secondary mechanism using the TAK779 analogs and the above natural products is the next assignment.

In conclusion, we synthesized fluorescence-labeled TAK779 analogs (**2a**–**d**) as chemical probes. Compounds (**2b**–**d**) penetrated the cell membranes and localized in the cells. Although the binding properties of **2b**–**d** for CCR5 could not be determined, TAK779 analog (**2c**) exhibited cell-penetrating property and localization behavior. In order to preferentially bind to CCR5, the physiological properties of the TAK779 analogs need to devised, including the redesign of the TAK779 analog structure based on the structure activity relationships and the selected spacers with its length and flexibility. Simultaneously, the conjugation site with spacer units and fluorophores is an important factor. The TAK779 analogs (**2b**–**d**) showed no cytotoxicity, and additionally, membrane permeation and localization inside the cell were observed. It is also interesting to develop it as an organelle-selective fluorescent agent by using this phenomenon. By the comparison of known materials, the mechanism of action in detail may become clear. Thereby, it is possible to produce useful materials with unique functionalities. The design and synthesis of the TAK779 analogs (**2b**–**d**) were performed and provided useful information for the next research. Further investigation of the binding properties and localization using the modified analogs with low cytotoxicity is currently underway.

## 3. Experimental

### 3.1. General

All solvents were of reagent grade. THF was distilled from sodium and benzophenone ketyl. CH_2_Cl_2_ was distilled from CaH_2_. All commercial reagents were of the highest purity available. Analytical TLC was performed on silica gel (60 F-254, Plates 0.25 mm). Column chromatography was carried out on silica gel 60 N (Kanto Chemical Co., Tokyo, Japan, 40–100 µm). ^1^H (600, 500 or 400 MHz) and ^13^C (150, 125 or 100 MHz) NMR spectra were recorded on a JEOL JNM-ECX 600, JNM-ECX 500, or JNM-ECX 400 (JEOL, Ltd., Tokyo, Japan). Chemical shifts are expressed in ppm relative to TMS (0 ppm), CHCl_3_ (7.26 ppm for ^1^H and 77.0 ppm for ^13^C), or DMSO-d_6_ (2.50 ppm for ^1^H and 39.5 ppm for ^13^C). IR spectra were obtained on a JASCO FT/IR-460 Plus spectrometer (Tokyo, Japan). High-resolution mass spectra (HRMS) were obtained on a JEOL The AccuTOF JMS-T100LC (ESI). Melting points were measured on an AS ONE ATM-02 (Osaka, Japan). Optical rotations were measured on a JASCO DIP-371. Ultraviolet and visible absorption spectra were obtained on a HITACHI U-1900 or U-2900 (Chiyoda, Japan). Fluorescence spectra were recorded on a JASCO FP-8200 or HITACHI F-2700. Absorbances were measured on a CORONA ELECTRIC MTP-310 Lab (Hitachinaka, Japan).

### 3.2. N-Cyclohexyl-N-(4-nitrobenzyl)-11-undecanamido Acetate (***8***)

(1) To a solution of 11-bromoundecanoic acid (0.85 g, 3.21 mmol) in THF were added DCC (0.66 g, 3.20 mmol), DMAP (0.04 g, 0.33 mmol), and **4a** (0.50 g, 2.13 mmol). The reaction mixture was stirred overnight at room temperature, filtered through a celite pad, to which was added AcOEt and H_2_O, and separated. The aqueous layer was extracted with AcOEt and the combined organic layer was washed successively with water and brine, dried over MgSO_4_, and concentrated in vacuo. The residue was purified by column chromatography (hexane/AcOEt = 5:1) to give amide (**4c**) (0.94 g, 2.01 mmol, 95%) as a brown oil. ^1^H-NMR (CDCl_3_, 600 MHz) δ 8.10 (m, 2H), 7.33 (m, 2H), 4.55 (m, 2H), 4.50 (m, 1H), 3.35–3.31 (m, 2H), 2.42–1.73 (m, 6H), 1.67–1.16 (m, 21H), 1.00 (m, 1H). ^13^C-NMR (CDCl_3_, 150 MHz) δ 173.33, 173.29, 147.6, 146.9, 146.7, 146.4, 127.3, 126.5, 123.7, 123.3, 57.3, 53.4, 46.2, 44.3, 33.8, 33.7, 33.4, 32.6, 31.9, 30.7, 30.5, 29.22, 29.16, 29.1, 29.0, 28.49, 28.45, 27.90, 27.87, 25.7, 25.5, 25.23, 25.20, 25.1, 24.9. IR (film, cm^−1^) ν_max_ 2927, 2853, 1644, 1601, 1521, 1408, 1343, 1109, 1013, 858, 736. ESI-HRMS *m*/*z* 481.2036 [M + H]^+^ (calcd. for C_24_H_38_BrN_2_O_3_, 481.2066).

(2) To a solution of amide (**4c**) (1.50 g, 3.12 mmol) in DMF was added NaOAc (1.23 g, 15.0 mmol). The reaction mixture was refluxed for 4 h, diluted with AcOEt and H_2_O. The aqueous layer was extracted with AcOEt and the combined organic layer was washed successively with water and brine, dried over MgSO_4_, and concentrated in vacuo. The residue was purified by column chromatography (hexane/AcOEt = 3:1) to give **8** (1.52 g, quant.) as a brown oil. ^1^H-NMR (CDCl_3_, 600 MHz) δ 8.11 (m, 2H), 7.33 (m, 2H), 4.56–4.54 (m, 2H), 4.60 (m, 1H), 3.98 (m, 2H), 2.43–2.08 (m, 2H), 1.98 (m, 3H), 1.76–1.51 (m, 9H), 1.36–1.13 (m, 16H), 0.98 (m, 1H). ^13^C-NMR (CDCl_3_, 150 MHz) δ 173.41, 173.37, 161.0, 147.7, 147.0, 146.7, 146.5, 127.4, 126.5, 123.7, 123.3, 64.4, 57.4, 53.4, 46.2, 44.4, 33.8, 33.4, 32.0, 30.6, 29.30, 29.27, 29.24, 29.22, 29.14, 29.11, 29.04, 28.99, 28.4, 25.7, 25.5, 25.3, 25.2, 25.1, 25.0. IR (film, cm^−1^) ν_max_ 2928, 2854, 1736, 1646, 1521, 1343, 1238, 1109, 1033, 737. ESI-HRMS *m*/*z* 461.2986 [M + H]^+^ (calcd. for C_26_H_41_N_2_O_5_ 461.3016).

### 3.3. N-Cyclohexyl-N-(4-{N-[6-(4-methylphenyl)-2H-1-benzopyran-3-carbonyl]amino}benzyl)-11-undecanamido Acetate (***9***)

(1) To a solution of **8** (0.62 g, 1.35 mmol) in AcOEt was added 5% Pt/C (2 mg). The reaction mixture was vigorously stirred at room temperature under a hydrogen atmosphere for 4 h and filtered through a celite pad, which was washed with AcOEt. The filtrate was concentrated in vacuo. The residue was purified by column chromatography (AcOEt) to give amine (**8a**) (0.57 g, 1.32 mmol, 98%) as a yellow oil. ^1^H-NMR (CDCl_3_, 600 MHz) δ 6.94 (m, 2H), 6.56 (m, 2H), 4.49 (m, 1H), 4.36 (m, 2H), 3.99 (m, 2H), 3.70 (s, 2H), 2.38–2.16 (m, 2H), 1.99 (s, 3H), 1.73–1.53 (m, 9H), 1.41–1.18 (m, 16H), 0.98 (m, 1H). ^13^C-NMR (CDCl_3_, 150 MHz) δ: 173.6, 173.0, 161.0, 145.3, 144.8, 129.8, 128.12, 128.05, 126.6, 114.9, 114.8, 64.4, 60.1, 57.4, 53.5, 46.2, 43.8, 33.8, 33.6, 32.0, 30.5, 29.3, 29.2, 29.1, 29.01, 28.98, 28.4, 25.9, 25.6, 25.5, 25.4, 25.3, 25.1. IR (film, cm^−1^) ν_max_ 3351, 2927, 2853, 1735, 1629, 1518, 1419, 1363, 1239, 1176, 1035, 816. ESI-HRMS *m*/*z* 431.3273 [M + H]^+^ (calcd. for C_26_H_43_N_2_O_3_ 431.3274).

(2) To a solution of carboxylic acid (**S5**) (0.10 g, 0.38 mmol) in CHCl_3_ were added SOCl_2_ (0.10 mL, 1.38 mmol) and a DMF (1 drop) on ice. The reaction mixture was stirred at room temperature under nitrogen atmosphere for 2 h and concentrated in vacuo to give **3**. To a solution of **3** in THF was dropwise added a solution of amine (**8a**) (0.20 g, 0.46 mmol) and TEA (0.14 mL, 1.00 mmol) in THF on ice. The reaction mixture was stirred overnight at room temperature under nitrogen atmosphere, then diluted with AcOEt and H_2_O. The aqueous layer was extracted with AcOEt and the combined organic layer was washed successively with water and brine, dried over MgSO_4_, and concentrated in vacuo. The residue was purified by column chromatography (hexane/AcOEt = 2:1) to give **9** (0.25 g, 0.37 mmol, 97%) as a yellow oil. ^1^H-NMR (CDCl_3_, 500 MHz) δ 8.87 (m, 1H), 7.54 (m, 2H), 7.41 (m, 4H), 7.26 (m, 1H), 7.22 (d, *J* = 8.5 Hz, 2H), 7.08 (m, 2H), 6.91 (m, 1H), 5.11 (s, 2H), 4.49 (m, 2H), 4.54–3.61 (m, 1H), 4.08 (m, 2H), 2.45–2.17 (m, 2H), 2.38 (s, 3H), 2.04 (m, 3H), 1.78–1.55 (m, 9H), 1.43–1.19 (m, 16H), 1.01 (m, 1H). ^13^C-NMR (CDCl_3_, 125 MHz) δ: 174.2, 173.5, 163.6, 163.5, 154.1, 137.24, 137.20, 136.73, 136.69, 136.3, 135.6, 134.8, 134.7, 134.2, 129.8, 129.7, 129.4, 128.1, 128.0, 127.3, 127.2, 126.58, 126.55, 126.3, 126.1, 121.3, 121.2, 120.6, 120.3, 116.31, 116.26, 65.00, 64.96, 64.6, 57.8, 53.7, 46.2, 44.2, 34.3, 34.1, 33.8, 32.1, 30.7, 29.5, 29.33, 29.28, 29.14, 29.09, 28.49, 28.46, 25.9, 25.8, 25.7, 25.6, 25.3, 25.1, 24.9. IR (film, cm^−1^) ν_max_ 2927, 2854, 1735, 1600, 1531, 1488, 1411, 1319, 1247, 1020, 812. ESI-HRMS *m*/*z*: 679.4127 [M + H]^+^ (calcd. for C_43_H_55_N_2_O_5_ 679.4111).

### 3.4. N-[4-({[N-(11-Azido-1-oxoundecanyl)-N-cyclohexyl]amino}methyl)phenyl]-6-(4-methylphenyl)-2H-1-benzopyran-3-carboxamide (***10***)

(1) To a solution of **9** (0.25 g, 0.37 mmol) in MeOH was added K_2_CO_3_ (0.14 g, 1.01 mmol). The reaction mixture was stirred at room temperature for 2 h, diluted with AcOEt, and saturated NH_4_Cl aq. The aqueous layer was extracted with AcOEt and the combined organic layer was washed with water and brine, dried over MgSO_4_, and concentrated in vacuo. The residue was purified by column chromatography (hexane/AcOEt = 1:1) to give alcohol (**9a**) (0.26 g, quant.) as a brown oil. ^1^H-NMR (CDCl_3_, 600 MHz) δ 9.15 (m, 1H), 7.50 (m, 2H), 7.40 (m, 4H), 7.26 (m, 1H), 7.20 (d, *J* = 7.8 Hz, 2H), 7.07 (m, 2H), 6.89 (m, 1H), 5.09 (s, 2H), 4.47 (m, 2H), 4.62 (m, 1H), 3.59 (m, 2H), 2.43–2.16 (m, 3H), 2.36 (s, 3H), 1.76–1.49 (m, 9H), 1.40–1.16 (m, 16H), 1.00 (m, 1H). ^13^C-NMR (CDCl_3_, 150 MHz) δ: 174.2, 173.6, 163.7, 163.6, 154.0, 137.2, 137.15, 137.10, 136.60, 136.57, 136.4, 135.4, 134.7, 134.6, 134.0, 129.63, 129.56, 129.4, 128.2, 128.1, 127.10, 127.06, 126.50, 126.48, 126.3, 126.2, 126.0, 121.3, 121.2, 120.5, 120.4, 116.2, 116.1, 64.92, 64.89, 62.6, 57.8, 53.7, 46.2, 44.2, 33.9, 33.7, 32.60, 32.55, 32.0, 30.6, 29.29, 29.27, 29.25, 29.21, 29.17, 29.16, 29.13, 29.10, 25.8, 25.7, 25.61, 25.56, 25.4, 25.3, 25.0. IR (film, cm^−1^) ν_max_ 3298, 2928, 2854, 1653, 1620, 1533, 1487, 1412, 1320, 1253, 1136, 1020, 918, 812, 755, 665. ESI-HRMS *m*/*z* 659.3833 [M + Na]^+^ (calcd. for C_41_H_52_N_2_NaO_4_ 659.3825).

(2) To a solution of alcohol (**9a**) (1.42 g, 2.23 mmol) in CH_2_Cl_2_ were added TEA (2.80 mL, 20.1 mmol), TsCl (1.90 g, 9.97 mmol), and DMAP (0.12 g, 0.98 mmol). The reaction mixture was stirred at room temperature for 1.3 h, diluted with CH_2_Cl_2_, and saturated with NH_4_Cl aq. The aqueous layer was extracted with CH_2_Cl_2_, and the combined organic layer was washed with water and brine, dried over MgSO_4_, and concentrated in vacuo. The residue was purified by column chromatography (hexane/AcOEt = 2:1–1:1) to give tosylate (**9b**) (1.70 g, 2.15 mmol, 96%) as a yellow oil. ^1^H-NMR (CDCl_3_, 600 MHz) δ 8.62–6.88 (m, 17H), 5.12 (m, 2H), 4.47 (m, 2H), 4.52 (m, 1H), 3.99 (m, 2H), 2.44–2.16 (m, 8H), 1.77–1.58 (m, 9H), 1.40–1.15 (m, 16H), 1.00 (m, 1H). ^13^C-NMR (CDCl_3_, 150 MHz) δ: 174.1, 173.5, 163.6, 163.4, 154.1, 144.6, 143.2, 139.3, 137.19, 137.15, 137.0, 136.73, 136.69, 136.3, 135.7, 134.8, 134.7, 134.4, 133.1, 132.99, 132.96, 129.7, 129.5, 129.4, 128.0, 127.8, 127.4, 126.8, 126.6, 126.33, 126.28, 126.2, 120.3, 116.3, 116.2, 70.7, 64.9, 57.8, 53.7, 46.3, 44.2, 33.9, 33.7, 32.1, 30.6, 29.4, 29.3, 29.2, 29.11, 29.08, 28.8, 28.7, 25.9, 25.7, 25.4, 25.2, 25.1, 21.5, 21.4, 21.0. IR (film, cm^−1^) ν_max_ 2927, 2854, 1599, 1530, 1488, 1412, 1356, 1249, 1175, 1097, 926, 812, 755, 663, 554.

(3) To a solution of tosylate (**9b**) (70 mg, 89 µmol) in DMF was added NaN_3_ (65 mg, 1.0 mmol). The reaction mixture was stirred at room temperature for 10 h, diluted with AcOEt and H_2_O. The aqueous layer was extracted with AcOEt and the combined organic layer was washed with water and brine, dried over MgSO_4_, and concentrated in vacuo. The residue was purified by column chromatography (hexane/AcOEt = 1:1) to give **10** (45 mg, 67.5 mmol, 77%) as a yellow oil. ^1^H-NMR (CDCl_3_, 600 MHz) δ 9.07 (m, 1H), 7.87–6.88 (m, 12H), 5.10 (m, 2H), 4.48 (m, 2H), 4.53 (m, 1H), 3.22 (m, 2H), 2.45–2.18 (m, 5H), 1.77–1.52 (m, 9H), 1.39–1.19 (m, 16H), 1.02 (m, 1H). ^13^C-NMR (CDCl_3_, 150 MHz) δ: 174.2, 173.5, 163.6, 163.5, 154.0, 137.3, 137.12, 137.08, 136.60, 136.57, 136.4, 135.4, 134.62, 134.59, 134.0, 129.7, 129.6, 129.5, 129.4, 128.1, 127.9, 127.5, 127.32, 127.27, 127.1, 126.7, 126.5, 126.4, 126.3, 126.2, 126.0, 121.24, 121.20, 120.7, 120.5, 120.35, 120.28, 116.20, 116.15, 64.93, 64.91, 57.7, 53.7, 51.3, 46.2, 44.1, 34.0, 33.7, 32.0, 30.6, 29.4, 29.23, 29.19, 28.94, 28.92, 28.6, 26.53, 26.51, 25.8, 25.7, 25.6, 25.5, 25.4, 25.3, 25.0, 20.9. IR (film, cm^−1^) ν_max_ 2928, 2854, 2094, 1718, 1702, 1619, 1533, 1516, 1487, 1467, 1451, 1411, 1319, 1287, 1252, 1190, 1137, 1066, 1019, 1002, 917, 812, 755, 664.

### 3.5. N-(4-{[(N-{11-[4-(7-Hydroxy-4-methyl-2-oxo-2H-1-benzopyran-3-)-1,2,3-triazolyl]-1-oxoundecanyl}-N-cyclohexyl)amino]methyl}phenyl)-6-(4-methylphenyl)-2H-1-benzopyran-3-carboxamide (***2a***)

(1) To a solution of **10** (250 mg, 0.38 mmol) in CH_2_Cl_2_ were added **5** (160 mg, 0.66 mmol), CuI (76.0 mg, 0.40 mmol), and DIPEA (175 µL, 1.00 mmol). The reaction mixture was stirred at room temperature for 3 h, diluted with CH_2_Cl_2_, and saturated with NH_4_Cl aq. The aqueous layer was extracted with CH_2_Cl_2_, and the combined organic layer was washed with water and brine, dried over MgSO_4_, and concentrated in vacuo. The residue was purified by column chromatography (hexane/AcOEt = 1:1) to give triazole (**10a**) (51 mg, 56.7 µmol, 15%) as a brown oil. ^1^H-NMR (CDCl_3_, 600 MHz) δ 8.60 (m, 1H), 8.13 (m, 1H), 7.66–6.83 (m, 15H), 5.24–5.07 (m, 4H), 4.49 (m, 2H), 4.54 (m, 1H), 4.38 (m, 2H), 3.47 (m, 3H), 2.36 (s, 3H), 2.16 (s, 3H), 1.93 (m, 2H), 1.77–1.56 (m, 9H), 1.42–1.21 (m, 16H), 1.02 (m, 1H). ^13^C-NMR (CDCl_3_, 150 MHz) δ 174.1, 173.5, 163.7, 163.5, 160.6, 160.5, 159.9, 159.6, 159.5, 154.5, 154.1, 153.4, 153.24, 153.17, 146.0, 141.1, 137.14, 137.05, 136.7, 134.7, 129.8, 129.7, 129.4, 128.0, 127.8, 127.4, 127.3, 126.9, 126.55, 126.52, 126.3, 126.2, 125.6, 121.1, 120.4, 116.2, 114.3, 113.6, 113.5, 103.6, 103.3, 94.3, 82.2, 65.0, 57.7, 56.3, 53.7, 51.1, 50.4, 46.3, 44.2, 33.8, 33.7, 32.1, 30.7, 30.1, 30.0, 29.6, 29.3, 29.24, 29.16, 29.1, 29.0, 28.94, 28.87, 28.8, 28.7, 28.6, 26.4, 26.3, 26.2, 25.9, 25.7, 25.6, 25.4, 25.3, 25.24, 25.17, 21.1, 21.0, 17.0, 16.6. IR (film, cm^−1^) ν_max_ 2928, 2859, 1609, 1514, 996, 754. ESI-MS *m*/*z* 906.4 [M + H]^+^ (calcd. for C_55_H_64_N_5_O_7_; 906.5).

(2) A solution of triazole (**10a**) (12.8 mg, 14.2 µmol) in CH_2_Cl_2_ was dropwise added to 50% TFA/CH_2_Cl_2_. The reaction mixture was stirred at room temperature for 1.5 h and concentrated in vacuo. The residue was diluted with CHCl_3_ and H_2_O. The aqueous layer was extracted with CHCl_3_, and the combined organic layer was washed with 1M NaOH, dried over MgSO_4_, and concentrated in vacuo. **2a** (13 mg, quant.) was obtained as a yellow oil. ^1^H-NMR (CDCl_3_, 600 MHz) δ 9.65 (m, 1H), 9.00 (m, 1H), 8.03 (m, 1H), 7.90–6.61 (m, 15H), 5.09 (m, 2H), 4.57 (m, 3H), 4.25 (m, 2H), 2.67–2.18 (m, 8H), 1.88–1.58 (m, 9H), 1.49–1.17 (m, 16H), 1.05 (m, 1H). ^13^C-NMR (CDCl_3_, 150 MHz) δ 174.3, 163.8, 154.7, 154.2, 147.2, 143.7, 136.7, 136.1, 135.1, 129.9, 129.5, 128.2, 127.6, 126.9, 126.64, 126.59, 126.5, 126.4, 125.4, 121.9, 121.5, 120.6, 116.3, 113.9, 103.2, 65.1, 58.0, 51.1, 50.3, 46.5, 45.0, 33.7, 32.0, 30.7, 30.0, 29.6, 29.3, 29.1, 28.9, 28.84, 28.78, 28.7, 28.6, 26.1, 25.9, 25.8, 25.7, 25.5, 25.1, 21.1, 16.6. IR (film, cm^−1^) ν_max_ 2923, 2854, 1713, 1601, 1512, 1487, 1318, 1214, 813, 750. ESI-HRMS *m*/*z* 862.4517 [M+H]^+^ (calcd. for C_53_H_60_N_5_O_6_ 862.4544).

### 3.6. N-{4-[(Cyclohexylamino)methyl]phenyl}-6-(4-methylphenyl)-2H-chromene-3-carboxamide (***11***)

(1) To a solution of carboxylic acid (**S5**) (300 mg, 1.13 mmol) in CHCl_3_ (2.50 mL) was added SOCl_2_ (0.66 mL, 9.10 mmol) on ice. The reaction mixture was stirred at room temperature for 10 min, then DMF (71 µL, 0.92 mmol) was added to the mixture. The mixture was stirred at room temperature for 2 h and concentrated in vacuo to give **3**. To a solution of **3** in THF (1.30 mL) were dropwise added a solution of **4b** (446 mg, 1.47 mmol) and Et_3_N (613 µL, 4.40 mmol) in THF (1.30 mL) on ice. The reaction mixture was stirred at room temperature for 19 h, concentrated in vacuo, and diluted with CHCl_3_ and NH_4_Cl aq. The aqueous layer was extracted with CHCl_3_, and the combined organic layer was washed with brine, dried over MgSO_4_, and concentrated in vacuo. The residue was purified by column chromatography (hexane/AcOEt = 6:1–4:1) to give carbamate (**3a**) (625 mg, 1.13 mmol, quant.) as a pale-yellow powder. ^1^H-NMR (CDCl_3_, 600 MHz) δ 8.31 (m, 1H), 7.52 (m, 2H), 7.44 (dd, *J* = 8.4, 2.4 Hz, 1H), 7.40 (d, *J* = 7.8 Hz, 2H), 7.31 (s, 1H), 7.27 (d, *J* = 1.8 Hz, 1H), 7.22 (d, *J* = 8.4 Hz, 2H), 7.17 (d, *J* = 7.2 Hz, 2H), 6.92 (d, *J* = 8.4 Hz, 1H), 5.11 (s, 2H), 4.38–3.64 (m, 3H), 2.38 (s, 3H), 1.67–1.18 (m, 18H), 0.99 (m, 1H). ^13^C-NMR (CDCl_3_, 150 MHz) δ 163.4, 155.8, 154.1, 137.2, 136.7, 136.5, 136.2, 134.8, 129.8, 129.5, 127.8, 127.3, 126.9, 126.6, 126.3, 121.1, 120.0, 116.3, 79.7, 64.9, 55.3, 45.8, 31.1, 28.4, 25.8, 25.4, 21.0. IR (KBr, cm^−1^) ν_max_ 3286, 3195, 3126, 3058, 2971, 2935, 2852, 1687, 1644, 1600, 1534, 1515, 1486, 1447, 1404, 1365, 1326, 1279, 1247, 1164, 1148, 1100, 1022, 1006, 962, 944, 907, 880, 862, 830, 810, 780, 510. ESI-HRMS *m*/*z* 575.2879 [M + Na]^+^ (calcd. for C_35_H_40_N_2_NaO_4_ 575.2886. m.p. 188–189 °C.

(2) To a solution of carbamate (**3a**) (2.03 g, 3.67 mmol) in CH_2_Cl_2_ (18.0 mL) was dropwise added TFA (1.00 mL) on ice. The reaction mixture was stirred at room temperature for 30 min, then TFA (1.00 mL) was added to the mixture on ice. The mixture was stirred at room temperature for 1.5 h, then CH_2_Cl_2_ (18.0 mL) and TFA (2.0 mL) were added to the mixture on ice. The mixture was stirred at room temperature for 2 h, diluted with CH_2_Cl_2_ and 1 M NaHCO_3_ aq. on ice. The aqueous layer was extracted with CH_2_Cl_2_, and the combined organic layer was washed with brine, dried over MgSO_4_, and concentrated in vacuo. The residue was purified by column chromatography (CHCl_3_/MeOH = 95:5–70:30) to give **11** (1.47 g, 3.25 mmol, 88%) as a yellow oil. ^1^H-NMR (CDCl_3_, 500 MHz) δ 8.05 (s, 1H), 7.51 (d, *J* = 8.5 Hz, 2H), 7.42 (dd, *J* = 8.5, 2.0 Hz, 1H), 7.38 (d, *J* = 8.0 Hz, 2H), 7.26 (d, *J* = 8.5 Hz, 2H), 7.23 (d, *J* = 2.5 Hz, 1H), 7.21 (d, *J* = 8.0 Hz, 2H), 7.10 (s, 1H), 6.89 (d, *J* = 8.5 Hz, 1H), 5.05 (s, 2H), 3.76 (s, 2H), 2.51–2.45 (m, 1H), 2.38 (s, 3H), 2.05 (s, 1H), 1.91 (m, 2H), 1.73 (m, 2H), 1.62 (m, 1H), 1.19 (m, 5H). ^13^C-NMR (CDCl_3_, 150 MHz) δ 163.4, 154.0, 137.1, 136.8, 136.7, 136.3, 134.9, 129.9, 129.5, 128.8, 127.7, 127.3, 126.6, 126.4, 120.9, 120.3, 116.3, 64.9, 56.1, 50.2, 33.2, 26.0, 24.9, 21.0. IR (film, cm^−1^) ν_max_ 3299, 3189, 3119, 3023, 2926, 2852, 1651, 1600, 1518, 1487, 1450, 1412, 1320, 1254, 1215, 1186, 1135, 1111, 1020, 1006, 911, 891, 811, 756, 665. ESI-HRMS *m*/*z*: 453.2571 [M + H]^+^ (calcd. for C_30_H_33_N_2_O_2_ 453.2542).

### 3.7. N-{2-[2-(2-Azidoethoxy)ethoxy]ethyl}-N′-cyclohexyl-N′-({4-[6-(4-methylphenyl)-2H-chromene-3-amido]phenyl}methyl)pentanediamide (***12***)

(1) To a solution of **11** (400 mg, 0.88 mmol) in MeCN (3.00 mL) were added Et_3_N (616 µL, 4.42 mmol) and glutaric anhydride (303 mg, 2.66 mmol). The reaction mixture was stirred at room temperature for 80 min, then glutaric anhydride (202 mg, 1.77 mmol) was added to the mixture. The mixture was stirred at room temperature for 70 min, concentrated in vacuo, and diluted with AcOEt and 1 M HCl. The aqueous layer was extracted with AcOEt and the combined organic layer was washed with brine, dried over MgSO_4_, and concentrated in vacuo. The residue was purified by column chromatography (CHCl_3_/MeOH = 95:5) to give carboxylic acid (**11a**) (382 mg, 0.67 mmol, 76%) as a yellow oil. ^1^H-NMR (CDCl_3_, 500 MHz) δ 10.23 (s, 1H), 8.90 (m, 1H), 7.51 (m, 2H), 7.39 (m, 3H), 7.30 (m, 1H), 7.24 (m, 1H), 7.19 (d, *J* = 7.0 Hz, 2H), 7.05 (m, 2H), 6.88 (d, *J* = 8.5 Hz, 1H), 5.05 (s, 2H), 4.48 (m, 3H), 2.45 (m, 2H), 2.36 (s, 3H), 2.27 (m, 2H), 1.93 (m, 2H), 1.63 (m, 5H), 1.38–1.15 (m, 4H), 0.97 (m, 1H). ^13^C-NMR (CDCl_3_, 125 MHz) δ 177.3, 177.2, 173.6, 173.0, 163.9, 163.7, 154.0, 137.1, 137.0, 136.9, 136.7, 136.6, 136.3, 135.1, 134.7, 134.0, 129.72, 129.65, 129.4, 128.4, 128.2, 127.1, 127.0, 126.9, 126.5, 126.3, 126.1, 121.2, 121.1, 120.9, 120.6, 116.22, 116.18, 64.9, 64.8, 57.6, 53.9, 46.2, 44.4, 33.1, 32.9, 32.7, 32.5, 31.8, 30.5, 25.7, 25.5, 25.2, 25.0, 20.9, 20.6, 20.4. IR (film, cm^−1^) ν_max_ 3296, 3191, 3119, 3017, 2932, 2856, 1709, 1651, 1600, 1530, 1514, 1487, 1469, 1450, 1412, 1320, 1286, 1251, 1216, 1194, 1138, 1019, 1003, 914, 812, 755, 666. ESI-HRMS *m*/*z* 589.2693 [M + Na]^+^ (calcd. for C_35_H_38_N_2_NaO_5_ 589.2678).

(2) To a solution of carboxylic acid (**11a**) (176 mg, 0.31 mmol) in CH_2_Cl_2_ (0.50 mL) were added DIPEA (307 µL, 1.76 mmol), WSC•HCl (135 mg, 0.70 mmol), HOBt•H_2_O (108 mg, 0.71 mmol), and a solution of **7a** (68 mg, 0.39 mmol) in CH_2_Cl_2_ (0.20 mL). The reaction mixture was stirred at room temperature for 13 h, diluted with CH_2_Cl_2_ and H_2_O. The aqueous layer was extracted with CH_2_Cl_2_, and the combined organic layer was washed with brine, dried over MgSO_4_, and concentrated in vacuo. The residue was purified by column chromatography (CHCl_3_/MeOH = 100:0–97:3) to give **12** (200 mg, 0.28 mmol, 89%) as a yellow oil. ^1^H-NMR (CDCl_3_, 600 MHz) δ 9.15 (m, 1H), 7.53 (m, 2H), 7.35 (m, 3H), 7.29 (m, 1H), 7.20 (s, 1H), 7.15 (d, *J* = 7.8 Hz, 2H), 7.03 (m, 2H), 6.84 (m, 1H), 6.41 (m, 1H), 5.02 (s, 2H), 4.44–3.29 (m, 15H), 2.45–2.08 (m, 4H), 2.31 (s, 3H), 1.88 (m, 2H), 1.69–1.50 (m, 5H), 1.24 (m, 4H), 0.95 (m, 1H). ^13^C-NMR (CDCl_3_, 150 MHz) δ: 172.9, 172.5, 172.4, 163.5, 163.4, 153.79, 153.77, 137.0, 136.84, 136.82, 136.5, 136.42, 136.37, 135.1, 134.5, 134.4, 133.9, 129.4, 129.3, 129.2, 128.0, 127.8, 127.0, 126.93, 126.91, 126.3, 126.0, 125.9, 121.08, 121.05, 120.4, 120.2, 116.01, 115.98, 70.1, 69.83, 69.81, 69.7, 69.6, 69.45, 69.42, 64.73, 64.72, 57.2, 53.6, 50.2, 46.0, 44.0, 38.85, 38.82, 35.22, 35.16, 32.8, 32.4, 31.8, 30.3, 25.5, 25.4, 25.1, 24.9, 21.33, 21.27, 20.7. IR (film, cm^−1^) ν_max_ 3423, 3307, 3118, 3056, 3006, 2931, 2858, 2107, 1731, 1651, 1531, 1488, 1467, 1453, 1412, 1320, 1286, 1253, 1185, 1136, 1020, 997, 920, 899, 813, 755, 665, 643, 613, 572, 558, 513. ESI-HRMS *m*/*z* 745.3668 [M + Na]^+^ (calcd. for C_41_H_50_N_6_NaO_6_ 745.3690).

### 3.8. N′-Cyclohexyl-N-[2-(2-{2-[4-(7-hydroxy-4-methyl-2-oxo-2H-chromen-3-yl)-1H-1,2,3-triazol-1-yl]ethoxy}ethoxy)ethyl]-N′-({4-[6-(4-methylphenyl)-2H-chromene-3-amido]phenyl}methyl)pentanediamide (***2b***)

(1) To a solution of **12** (200 mg, 0.28 mmol) and **5** (72 mg, 0.29 mmol) in toluene (600 µL) were added CuI (79 mg, 0.41 mmol) and DIPEA (120 µL, 0.69 mmol). The reaction mixture was stirred at room temperature for 60 h, diluted with CH_2_Cl_2_ and NH_4_Cl aq. The aqueous layer was extracted with CH_2_Cl_2_, and the combined organic layer was washed with brine, dried over MgSO_4_, and concentrated in vacuo. The residue was purified by column chromatography (CHCl_3_/MeOH = 100:0–98:2) to give triazole (**12a**) (220 mg, 0.23 mmol, 82%) as a yellow oil. ^1^H-NMR (CDCl_3_, 600 MHz) δ 9.26 (m, 1H), 8.18 (m, 1H), 7.49 (m, 3H), 7.25 (m, 4H), 7.11 (s, 1H), 7.03 (d, *J* = 6.0 Hz, 2H), 6.96 (m, 2H), 6.85 (d, *J* = 9.0 Hz, 1H), 6.75 (m, 3H), 5.07 (m, 2H), 4.95 (s, 2H), 4.43–3.54 (m, 5H), 3.75 (m, 2H), 3.46–3.23 (m, 11H), 2.56 (m, 3H), 2.43–2.09 (m, 4H), 2.21 (s, 3H), 1.87 (m, 2H), 1.61–1.42 (m, 5H), 1.28–1.10 (m, 4H), 0.90 (m, 1H). ^13^C-NMR (CDCl_3_, 150 MHz) δ 172.7, 172.5, 172.2, 163.3, 163.2, 160.2, 160.1, 159.5, 153.5, 152.9, 149.53, 149.50, 140.5, 136.9, 136.51, 136.49, 136.3, 136.14, 136.11, 135.0, 134.1, 134.0, 133.7, 129.0, 127.8, 127.6, 126.8, 126.7, 126.3, 126.1, 126.0, 125.7, 125.6, 120.9, 120.8, 120.1, 119.9, 115.72, 115.69, 114.5, 113.2, 113.00, 112.98, 102.61, 102.59, 93.8, 69.9, 69.7, 69.6, 69.4, 68.7, 64.5, 57.0, 55.8, 53.3, 49.6, 45.8, 43.8, 38.8, 38.7, 35.0, 32.7, 32.3, 31.6, 30.2, 25.34, 25.28, 24.9, 24.7, 21.25, 21.22, 20.5, 16.4. IR (film, cm^−1^) ν_max_ 3320, 3190, 3123, 3056, 3006, 2932, 2858, 1709, 1659, 1616, 1578, 1530, 1516, 1488, 1467, 1453, 1412, 1385, 1337, 1320, 1279, 1254, 1221, 1143, 1073, 1019, 998, 970, 924, 814, 754, 711, 692, 665, 612, 512. ESI-HRMS *m*/*z* 989.4419 [M + Na]^+^ (calcd. for C_55_H_62_N_6_NaO_10_ 989.4425).

(2) Triazole (**12a**) (270 mg, 0.28 mmol) was added to 50% TFA/CH_2_Cl_2_ (2.00 mL) on ice. The reaction mixture was stirred at room temperature for 30 min, diluted with CH_2_Cl_2_ and NaHCO_3_ aq. on ice. The aqueous layer was extracted with CH_2_Cl_2_, and the combined organic layer was washed with brine, dried over MgSO_4_, and concentrated in vacuo. The residue was purified by column chromatography (CHCl_3_/MeOH = 100:0–97:3) to give **2b** (198 mg, 0.21 mmol, 77%) as a yellow oil. ^1^H-NMR (CDCl_3_, 600 MHz) δ 10.24 (m, 1H), 9.06 (m, 1H), 8.22 (m, 1H), 7.50 (m, 2H), 7.43 (m, 1H), 7.34 (m, 4H), 7.21 (m, 1H), 7.14 (d, *J* = 8.4 Hz, 2H), 7.02 (m, 3H), 6.80 (m, 2H), 6.67 (s, 1H), 5.04 (m, 2H), 4.47–3.63 (m, 5H), 3.80 (m, 2H), 3.52–3.33 (m, 8H), 2.58 (s, 3H), 2.52–2.20 (m, 4H), 2.31 (s, 3H), 1.96 (m, 2H), 1.60 (m, 5H), 1.36–1.13 (m, 4H), 0.90 (m, 1H). ^13^C-NMR (CDCl_3_, 150 MHz) δ: 173.4, 173.3, 173.2, 173.0, 163.70, 163.68, 161.21, 161.18, 161.15, 161.1, 153.9, 153.6, 150.9, 141.0, 136.9, 136.6, 136.2, 135.0, 134.6, 134.5, 134.0, 129.6, 129.5, 129.4, 128.3, 128.1, 127.0, 126.8, 126.7, 126.6, 126.5, 126.44, 126.38, 126.2, 126.0, 121.2, 121.1, 120.8, 120.6, 116.1, 113.7, 113.0, 111.8, 102.51, 102.45, 70.30, 70.25, 70.0, 69.9, 69.7, 69.0, 64.9, 64.8, 57.5, 53.9, 50.0, 46.2, 44.5, 39.3, 35.39, 35.35, 33.1, 32.6, 31.8, 30.5, 25.6, 25.5, 25.2, 25.0, 21.62, 21.58, 16.7. IR (film, cm^−1^) ν_max_ 3306, 3011, 2932, 2858, 1698, 1650, 1614, 1601, 1583, 1530, 1512, 1487, 1453, 1412, 1384, 1320, 1287, 1251, 1216, 1189, 1131, 1077, 1022, 982, 932, 894, 853, 814, 755, 687, 666, 612, 511. ESI-HRMS *m*/*z*: 945.4151 [M + Na]^+^ (calcd. for C_53_H_58_N_6_NaO_9_ 945.4163).

### 3.9. Tert-Butyl N-{2-[2-(2-{4-[Cyclohexyl({4-[6-(4-methylphenyl)-2H-chromene-3-amido]phenyl}methyl)carbamoyl]butanamido}ethoxy)ethoxy]ethyl}carbamate (***13***)

(1) To a solution of **11** (400 mg, 0.88 mmol) in MeCN (3.00 mL) were added TEA (616 µL, 4.42 mmol) and glutaric anhydride (303 mg, 2.66 mmol). The reaction mixture was stirred at room temperature for 80 min, then glutaric anhydride (202 mg, 1.77 mmol) was added to the mixture. The mixture was stirred at room temperature for 70 min, concentrated in vacuo, and diluted with AcOEt and 1M HCl. The aqueous layer was extracted with AcOEt and the combined organic layer was washed with brine, dried over MgSO_4_, and concentrated in vacuo. The residue was purified by column chromatography (CHCl_3_/MeOH = 95:5) to give carboxylic acid (**11a**) (382 mg, 0.67 mmol, 76%) as a yellow oil. ^1^H-NMR (CDCl_3_, 500 MHz) δ 10.23 (s, 1H), 9.89 (m, 1H), 7.51 (m, 2H), 7.40 (m, 3H), 7.30 (m, 1H), 7.24 (m, 1H), 7.19 (d, *J* = 7.0 Hz, 2H), 7.06 (m, 2H), 6.88 (d, *J* = 8.5 Hz, 1H), 5.05 (s, 2H), 4.41 (m, 3H), 2.45 (m, 2H), 2.36 (s, 3H), 2.28 (m, 2H), 1.97 (m, 2H), 1.63 (m, 5H), 1.38–1.15 (m, 4H), 0.96 (m, 1H). ^13^C-NMR (CDCl_3_, 125 MHz) δ 177.3, 177.2, 173.6, 173.0, 163.9, 163.7, 154.0, 137.1, 137.0, 136.9, 136.7, 136.6, 136.3, 135.1, 134.7, 134.0, 129.72, 129.65, 129.4, 128.4, 128.2, 127.1, 127.0, 126.9, 126.5, 126.3, 126.1, 121.17 121.14, 120.9, 120.6, 116.22, 116.18, 64.85, 64.82, 57.6, 53.9, 46.2, 44.4, 33.1, 32.9, 32.7, 32.5, 31.8, 30.5, 25.7, 25.5, 25.2, 25.0, 20.9, 20.6, 20.4. IR (film, cm^−1^) ν_max_ 3296, 3191, 3119, 3017, 2932, 2856, 1709, 1651, 1600, 1530, 1514, 1487, 1469, 1450, 1412, 1320, 1286, 1251, 1216, 1194, 1138, 1019, 1003, 914, 812, 755, 666. ESI-HRMS *m*/*z*: 589.2693 [M + Na]^+^ (calcd. for C_35_H_38_N_2_NaO_5_ 589.2678).

(2) To a solution of carboxylic acid (**11a**) (190 mg, 0.34 mmol) in CH_2_Cl_2_ (0.50 mL) were added DIPEA (292 µL, 1.68 mmol), WSC•HCl (129 mg, 0.67 mmol), HOBt•H_2_O (103 mg, 0.67 mmol), and a solution of **7b** (108 mg, 0.43 mmol) in CH_2_Cl_2_ (0.20 mL). The reaction mixture was stirred at room temperature for 14 h, diluted with CH_2_Cl_2_ and NaCl aq. The aqueous layer was extracted with CH_2_Cl_2_, and the combined organic layer was washed with brine, dried over MgSO_4_, and concentrated in vacuo. The residue was purified by column chromatography (CHCl_3_/MeOH = 100:0–98:2) to give **13** (241 mg, 0.30 mmol, 90%) as a yellow oil. ^1^H-NMR (CDCl_3_, 600 MHz) δ 8.79 (m, 1H), 7.53 (m, 2H), 7.40 (m, 3H), 7.29 (m, 1H), 7.27 (s, 1H), 7.20 (d, *J* = 7.8 Hz, 2H), 7.09 (m, 2H), 6.89 (m, 1H), 6.47 (m, 1H), 5.18 (s, 1H), 5.07 (s, 2H), 4.49–3.62 (m, 3H), 3.55–3.23 (m, 12H), 2.50–2.13 (m, 4H), 2.36 (s, 3H), 2.01–1.86 (m, 2H), 1.74–1.55 (m, 5H), 1.42–1.19 (m, 13H), 0.99 (m, 1H). ^13^C-NMR (CDCl_3_, 150 MHz) δ 173.2, 172.71, 172.65, 163.6, 163.5, 156.0, 154.0, 137.2, 137.1, 137.0, 136.74, 136.70, 136.4, 135.5, 134.80, 134.76, 134.2, 129.8, 129.7, 129.4, 128.0, 127.8, 127.3, 127.2, 126.5, 126.3, 126.1, 121.21, 121.17, 120.5, 120.3, 116.3, 116.2, 79.2, 70.1, 70.0, 69.72, 69.67, 65.0, 64.9, 57.5, 53.8, 46.2, 44.3, 40.2, 39.1, 39.0, 35.6, 35.5, 32.9, 32.5, 32.0, 30.6, 28.3, 25.8, 25.7, 25.3, 25.1, 21.6, 21.5, 21.0. IR (film, cm^−1^) ν_max_ 3319, 3002, 2975, 2932, 2859, 1695, 1652, 1532, 1488, 1453, 1412, 1390, 1365, 1320, 1283, 1252, 1173, 1136, 1109, 1020, 1006, 921, 858, 813, 755, 666, 612, 511. ESI-HRMS *m*/*z* 819.4286 [M + Na]^+^ (calcd. for C_46_H_60_N_4_NaO_8_ 819.4309).

### 3.10. N′-Cyclohexyl-N′-({4-[6-(4-methylphenyl)-2H-chromene-3-amido] phenyl}methyl)carbamoyl]butanamido-N′-(ethoxy)ethoxy]ethyl}carbamoyl)ethyl]-2,2-difluoro-4,6-dimethyl-1λ⁵,3-diaza-2-boratricyclo [7.3.0.0^3,7^]dodeca-1(12),4,6,8,10-pentaen-1-ylium-2-uide (***2c***)

(1) To a solution of **13** (1.25 g, 1.57 mmol) in CH_2_Cl_2_ (27.0 mL) was added TFA (3.00 mL) on ice. The reaction mixture was stirred at room temperature for 2 h, diluted with CH_2_Cl_2_ and an excess amount of NaHCO_3_ aq. on ice. The aqueous layer was extracted with CH_2_Cl_2_, and the combined organic layer was washed with brine, dried over MgSO_4_, and concentrated in vacuo to give crude amine (**13a**).

(2) To a solution of **6** (37 mg, 0.13 mmol) in CH_2_Cl_2_ (200 µL) were added DIPEA (110 µL, 0.63 mmol), WSC•HCl (49 mg, 0.26 mmol), HOBt•H_2_O (39 mg, 0.25 mmol), and a solution of the residue (**13a**) (90 mg) in CH_2_Cl_2_ (500 µL). The reaction mixture was stirred at room temperature for 17 h, diluted with CH_2_Cl_2_ and H_2_O. The aqueous layer was extracted with CH_2_Cl_2_, and the combined organic layer was washed with brine, dried over MgSO_4_, and concentrated in vacuo. The residue was purified by column chromatography (CHCl_3_/MeOH = 100:0–97:3) to give **2c** (85 mg, 87.5 µmol, 68%, 2 steps) as a red oil. ^1^H-NMR (CDCl_3_, 600 MHz) δ 8.93–8.73 (m, 1H), 7.59–7.47 (m, 2H), 7.38 (m, 3H), 7.26 (m, 1H), 7.22 (m, 1H), 7.19 (d, *J* = 7.2 Hz, 2H), 7.07 (m, 2H), 6.99 (m, 1H), 6.87 (m, 1H), 6.76 (m, 1H), 6.47 (m, 2H), 6.16 (m, 1H), 6.02 (m, 1H), 5.05 (m, 2H), 4.47–3.61 (m, 3H), 3.41 (m, 12H), 3.23 (m, 2H), 2.60–2.10 (m, 6H), 2.47 (m, 3H), 2.34 (s, 3H), 2.16 (m, 3H), 1.99–1.84 (m, 2H), 1.73–1.54 (m, 5H), 1.38–1.17 (m, 4H), 0.98 (m, 1H) ppm. ^13^C-NMR (CDCl_3_, 150 MHz) δ 173.5, 173.1, 172.67, 172.65, 172.5, 172.2, 171.9, 171.8, 171.7, 163.5, 163.4, 159.9, 159.8, 157.5, 157.3, 153.99, 153.96, 143.74, 143.67, 137.10, 137.07, 137.0, 136.7, 136.6, 136.4, 135.3, 134.91, 134.88, 134.7, 134.6, 134.2, 133.2, 129.7, 129.6, 129.4, 128.13, 128.10, 128.0, 127.8, 127.2, 127.11, 127.07, 126.5, 126.3, 126.1, 123.64, 123.60, 121.2, 120.5, 120.3, 117.00, 116.96, 116.20, 116.15, 70.03, 69.98, 69.60, 69.56, 64.92, 64.90, 57.4, 53.7, 46.1, 44.2, 39.1, 39.01, 38.95, 35.53, 35.48, 35.46, 35.2, 32.93, 32.89, 32.4, 31.9, 30.6, 25.7, 25.6, 25.3, 25.0, 24.6, 24.5, 21.5, 21.4, 20.9, 14.8, 11.1. IR (film, cm^−1^) ν_max_ 3413, 3307, 3056, 3006, 2931, 2858, 1721, 1651, 1605, 1530, 1488, 1443, 1412, 1373, 1321, 1252, 1216, 1194, 1175, 1136, 1087, 1064, 1019, 1000, 975, 910, 813, 754, 666, 612, 590, 512. ESI-HRMS *m*/*z* 993.4866 [M + Na]^+^ (calcd. for C_55_H_65_BF_2_N_6_NaO_7_ 993.4874).

### 3.11. 12-(2-{[(1S)-3-Carboxy-1-({2-[2-(2-{4-[cyclohexyl({4-[6-(4-methylphenyl)-2H-chromene-3-amido]phenyl}methyl)carbamoyl]butanamido}ethoxy)ethoxy]ethyl} carbamoyl)propyl]-carbamoyl}ethyl)-2,2-difluoro-4,6-dimethyl-1λ⁵,3-diaza-2-boratricyclo [7.3.0.0^3,^⁷]dodeca-1(12),4,6,8,10-pentaen-1-ylium-2-uide (***2d***)

(1) To a solution of **13** (1.25 g, 1.57 mmol) in CH_2_Cl_2_ (27.0 mL) was added TFA (3.0 mL) on ice. The reaction mixture was stirred at room temperature for 2 h, diluted with CH_2_Cl_2_ and 1 M NaHCO_3_ aq. on ice. The aqueous layer was extracted with CH_2_Cl_2_, and the combined organic layer was washed with brine, dried over MgSO_4_, and concentrated in vacuo to give activated aster (**6b**).

(2) To a solution of **6a** (5.0 mg, 10.5 µmol) in DMF (200 µL) were added DIPEA (9 µL, 51.7 µmol), WSC•HCl (4.0 mg, 20.9 µmol), HOBt•H_2_O (3.2 mg, 20.9 µmol), and a solution of the residue (**6b**) (8.0 mg) in DMF (200 µL). The reaction mixture was stirred at room temperature for 80 min, then a solution of the residue (**6b**) (5.0 mg) in DMF (100 µL) was added to the mixture. The mixture was stirred at room temperature for 40 min, diluted with AcOEt and NH_4_Cl aq. The aqueous layer was extracted with AcOEt and the combined organic layer was washed with brine, dried over MgSO_4_, and concentrated in vacuo. The residue was purified by column chromatography (CHCl_3_/MeOH = 100:0–96:4) to give ester (**14**) (7.0 mg, 6.05 µmol, 58%, 2 steps) as a red oil. ^1^H-NMR (CDCl_3_, 600 MHz) δ 8.60 (m, 1H), 7.54 (m, 2H), 7.40 (m, 3H), 7.23 (m, 4H), 7.10 (m, 3H), 6.99 (m, 1H), 6.83 (m, 1H), 6.88 (m, 1H), 6.81 (m, 2H), 6.17 (m, 1H), 6.02 (m, 1H), 5.06 (m, 2H), 4.49–3.64 (m, 4H), 3.60–3.29 (m, 12H), 3.21 (m, 2H), 2.60 (m, 3H), 2.48 (m, 3H), 2.37 (s, 3H), 2.31–2.15 (m, 8H), 2.04–1.77 (m, 4H), 1.75–1.56 (m, 5H), 1.40–1.19 (m, 13H), 1.00 (m, 1H). ^13^C-NMR (CDCl_3_, 150 MHz) δ 173.2, 172.95, 172.91, 172.8, 172.7, 172.5, 171.91, 171.86, 171.33, 171.27, 163.5, 163.4, 160.3, 160.2, 156.9, 156.8, 154.03, 154.01, 144.0, 143.9, 137.13, 137.09, 137.0, 136.75, 136.70, 136.4, 135.3, 135.1, 135.0, 134.75, 134.68, 134.3, 133.2, 129.7, 129.6, 129.5, 128.04, 127.99, 127.8, 127.23, 127.18, 127.0, 126.6, 126.3, 126.2, 123.71, 123.68, 121.20, 121.16, 120.5, 120.4, 120.3, 116.9, 116.3, 116.2, 80.7, 80.6, 70.14, 70.12, 70.06, 69.73, 69.70, 69.4, 64.9, 57.5, 53.7, 52.5, 46.2, 44.4, 39.2, 39.12, 39.07, 35.63, 35.58, 35.2, 35.1, 33.0, 32.5, 31.9, 31.5, 30.6, 28.0, 27.4, 25.8, 25.7, 25.4, 25.1, 24.4, 21.7, 21.5, 21.0, 14.8, 11.2. IR (film, cm^−1^) ν_max_ 3300, 3006, 2975, 2932, 2858, 1724, 1651, 1606, 1530, 1488, 1448, 1412, 1367, 1320, 1252, 1216, 1174, 1136, 1087, 1064, 1019, 1000, 974, 910, 813, 754, 666, 512. ESI-HRMS *m*/*z* 1178.5929 [M + Na]^+^ (calcd. for C_64_H_80_BF_2_N_7_NaO_10_ 1178.5925). [α]_D_^28^ −60.0 (*c* 0.1, CHCl_3_).

(3) 4 N HCl/AcOEt (0.30 mL) was added to ester (**14**) (3.0 mg, 2.59 µmol) on ice and the reaction mixture was stirred at room temperature for 2 h and concentrated in vacuo. The residue was purified by column chromatography (CHCl_3_/MeOH = 95:5) to give **2d** (2.1 mg, 1.91 µmol, 74%) as a red oil. ^1^H-NMR (CDCl_3_, 600 MHz) δ 8.68 (m, 1H), 7.54 (m, 2H), 7.40 (m, 3H), 7.23 (m, 5H), 7.06 (m, 4H), 6.87 (m, 2H), 6.76 (m, 1H), 6.15 (m, 1H), 6.03 (m, 1H), 5.06 (m, 2H), 4.57–3.65 (m, 4H), 3.55–3.27 (m, 12H), 3.21 (t, *J* = 7.2 Hz, 2H), 2.66–2.50 (m, 3H), 2.48 (m, 3H), 2.40–2.12 (m, 5H), 2.37 (s, 3H), 2.16 (m, 3H), 2.05–1.56 (m, 9H), 1.44–1.19 (m, 4H), 1.00 (m, 1H). ^13^C- NMR (CDCl_3_, 150 MHz) δ 175.24, 175.16, 174.0, 173.44, 173.36, 173.2, 172.2, 172.1, 171.40, 171.36, 163.55, 163.51, 160.29, 160.25, 156.9, 156.8, 156.5, 154.0, 143.9, 137.2, 137.1, 137.0, 136.8, 136.7, 136.5, 135.1, 134.9, 134.8, 134.7, 134.0, 133.2, 129.8, 129.7, 129.49, 129.47, 128.2, 128.1, 128.0, 127.9, 127.21, 127.18, 127.0, 126.7, 126.4, 126.3, 123.8, 121.25, 121.18, 120.6, 120.5, 120.4, 116.9, 116.3, 116.2, 70.2, 70.13, 70.09, 69.9, 69.8, 69.3, 64.97, 64.95, 57.7, 54.0, 52.21, 52.17, 46.3, 44.6, 39.3, 39.19, 39.15, 35.5, 35.4, 35.1, 33.1, 32.4, 31.9, 31.8, 30.6, 30.0, 29.8, 29.6, 27.9, 27.8, 25.74, 25.66, 25.3, 25.1, 24.44, 24.39, 21.7, 21.6, 21.0, 14.9, 11.2. IR (film, cm^−1^) ν_max_ 3298, 3006, 2932, 2858, 1720, 1650, 1606, 1530, 1488, 1445, 1412, 1376, 1321, 1252, 1216, 1175, 1137, 1087, 1063, 1017, 999, 974, 910, 813, 754, 665, 512. ESI-HRMS *m*/*z* 1122.5288 [M + Na]^+^ (calcd. for C_60_H_72_BF_2_N_7_NaO_10_ 1122.5299). [α]_D_^28^ −112.0 (*c* 0.1, CHCl_3_).

### 3.12. 12-[2-({2-[2-(2-Azidoethoxy)ethoxy]ethyl}carbamoyl)ethyl]-2,2-difluoro-4,6-dimethyl-1λ⁵,3-diaza-2-boratricyclo [7.3.0.0^3,^⁷]dodeca-1(12),4,6,8,10-pentaen-1-ylium-2-uide (***15***)

To a solution of BODIPY FL propionic acid (**6**) (80 mg, 0.27 mmol) in DMF (1.0 mL) was added DIPEA (238 µL, 1.37 mmol), WSC•HCl (105 mg, 0.55 mmol), HOBt•H_2_O (84 mg, 0.55 mmol), and 1-(2-aminoethoxy)-2-(2-azidoethoxy)ethane (**7a**) (95 mg, 0.55 mmol) at room temperature. After 3 h, the mixture was added to AcOEt and H_2_O. The organic layer was separated, washed with brine, dried over MgSO_4_, and concentrated in vacuo. The residue was purified with silica gel column chromatography (CHCl_3_/MeOH = 97:3) to give 12-[2-({2-[2-(2-azidoethoxy)ethoxy]ethyl}carbamoyl)ethyl]-2,2-difluoro-4,6-dimethyl-1λ^5^,3-diaza-2-boratricyclo [7.3.0.0^3,7^]dodeca-1(12),4,6,8,10-pentaen-1-ylium-2-uide (**15**) (70 mg, 0.16 mmol, 57%) as a red oil. ^1^H-NMR (CDCl_3_, 600 MHz) δ 7.06 (s, 1H), 6.86 (d, *J* = 4.2 Hz, 1H), 6.27 (d, *J* = 3.6 Hz, 1H), 6.09 (m, 2H), 3.63 (t, *J* = 4.8 Hz, 2H), 3.60–3.56 (m, 4H), 3.50 (t, *J* = 4.8 Hz, 2H), 3.43–3.40 (m, 2H), 3.34 (t, *J* = 4.8 Hz, 2H), 3.26 (t, *J* = 7.5 Hz, 2H), 2.61 (t, *J* = 7.5 Hz, 2H), 2.54 (s, 3H), 2.23 (s, 3H). ^13^C-NMR (CDCl_3_, 150 MHz) δ: 171.6, 160.0, 157.6, 143.7, 135.0, 133.3, 128.2, 123.7, 120.3, 117.4, 70.4, 70.2, 70.0, 69.8, 50.5, 39.1, 35.8, 24.7, 14.8, 11.2. IR (film, cm^−1^) ν_max_ 3418, 3311, 3096, 3070, 2924, 2870, 2108, 1656, 1606, 1529, 1488, 1442, 1345, 1305, 1252, 1174, 1136, 1185, 973, 933, 817, 750, 666. ESI-HRMS *m*/*z* 471.2132 [M + Na]^+^ (calcd. for C_20_H_27_BF_2_N_6_NaO_3_ 471.2103).

### 3.13. 12-{2-[(2-{2-[2-(4-Carboxybutanamido)ethoxy]ethoxy}ethyl)carbamoyl]ethyl}-2,2-difluoro-4,6-dimethyl-1λ^5^,3-diaza-2-boratricyclo [7.3.0.0^3,7^]dodeca-1(12),4,6,8,10-pentaen-1-ylium-2-uide (***16***)

To a solution of 12-[2-({2-[2-(2-Azidoethoxy)ethoxy]ethyl}carbamoyl)ethyl]-2,2-difluoro-4,6-dimethyl-1λ^5^,3-diaza-2-boratricyclo [7.3.0.0^3,7^]dodeca-1(12),4,6,8,10-pentaen-1-ylium-2-uide (**15**) (70 mg, 0.16 mmol) in MeOH (520 µL) was added glutaric anhydride (53 mg, 0.46 mmol) and 10% Pd/C (4.0 mg). The mixture was vigorously stirred at room temperature in H_2_ atmosphere. After 1 h, to the mixture was added glutaric anhydride (36 mg, 0.32 mmol) and Et_3_N (150 µL, 1.08 mmol) and stirred at room temperature. After 10 min, the mixture was filtrated with a celite pad and concentrated in vacuo. The residue was added to H_2_O, 1M HCl (adjusted to pH 2), and AcOEt. The organic layer was separated and washed with brine, dried over MgSO_4_, and concentrated in vacuo. The residue was purified with silica gel column chromatography (CHCl_3_/MeOH = 90:10) to give 12-{2-[(2-{2-[2-(4-Carboxybutanamido)ethoxy]ethoxy}ethyl)carbamoyl]ethyl}-2,2-difluoro-4,6-dimethyl-1λ^5^,3-diaza-2-boratricyclo [7.3.0.0^3,7^]dodeca-1(12),4,6,8,10-pentaen-1-ylium-2-uide (**16**) (63 mg, 0.12 mmol, 75%) as a red oil. ^1^H-NMR (CDCl_3_, 600 MHz) δ: 7.07 (s, 1H), 6.86 (d, *J* = 4.2 Hz, 1H), 6.59–6.54 (m, 2H), 6.27 (d, *J* = 3.6 Hz, 1H), 6.10 (s, 1H), 3.54 (m, 4H), 3.51 (t, *J* = 5.4 Hz, 2H), 3.49 (t, *J* = 5.4 Hz, 2H), 3.40 (m, 4H), 3.25 (t, *J* = 7.5 Hz, 2H), 2.63 (t, *J* = 7.5 Hz, 2H), 2.52 (s, 3H), 2.35 (t, *J* = 6.6 Hz, 2H), 2.25 (t, *J* = 7.2 Hz, 2H), 2.23 (s, 3H), 1.90 (quin, *J* = 7.2 Hz, 2H). ^13^C-NMR (CDCl_3_, 150 MHz) δ 175.9, 172.9, 172.4, 160.1, 157.4, 143.9, 135.0, 133.3, 128.2, 123.8, 120.4, 117.3, 70.1, 70.0, 69.8, 69.7, 39.2, 39.1, 35.6, 35.0, 32.7, 24.7, 20.7, 14.9, 11.2. IR (film, cm^−1^) ν_max_ 3306, 3094, 3307, 2932, 2871, 1725, 1649, 1607, 1530, 1488, 1442, 1347, 1253, 1175, 1137, 1086, 973, 899, 812, 752, 666. ESI-HRMS *m*/*z* 559.2509 [M + Na]^+^ (calcd. for C_25_H_35_BF_2_N_4_NaO_6_ 559.2515).

### 3.14. 12-(2-{[2-(2-{2-[4-(Diethylcarbamoyl)butanamido]ethoxy}ethoxy)ethyl]-carbamoyl}ethyl)-2,2-difluoro-4,6-dimethyl-1λ^5^,3-diaza-2-boratricyclo [7.3.0.0^3,7^]-dodeca-1(12),4,6,8,10-pentaen-1-ylium-2-uide (***17***)

To a solution of 12-{2-[(2-{2-[2-(4-Carboxybutanamido)ethoxy]ethoxy}ethyl)-carbamoyl]ethyl}-2,2-difluoro-4,6-dimethyl-1λ^5^,3-diaza-2-boratricyclo [7.3.0.0^3,7^]-dodeca-1(12),4,6,8,10-pentaen-1-ylium-2-uide (**16**) (10.0 mg, 18.6 µmol) in MeCN (300 µL) was added DIPEA (16 µL, 91.9 µmol), WSC•HCl (7.0 mg, 36.5 µmol), HOBt•H_2_O (6.0 mg, 39.2 µmol), and Et_2_NH (4 µL, 38.7 µmol) and stirred at room temperature. After 1 h, the mixture was added AcOEt and H_2_O and the organic layer was separated. The organic layer was washed with brine, dried over MgSO_4_, and concentrated in vacuo. The residue was purified with silica gel column chromatography (CHCl_3_/MeOH = 95:5) to give 12-(2-{[2-(2-{2-[4-(Diethylcarbamoyl)butanamido]ethoxy}ethoxy)ethyl]-carbamoyl}ethyl)-2,2-difluoro-4,6-dimethyl-1λ^5^,3-diaza-2-boratricyclo [7.3.0.0^3,7^]-dodeca-1(12),4,6,8,10-pentaen-1-ylium-2-uide (**17**) (6.0 mg, 10.1 µmol, 54%) as a red oil. ^1^H-NMR (CDCl_3_, 600 MHz) δ 7.08 (s, 1H), 6.87 (d, *J* = 3.6 Hz, 1H), 6.41 (t, *J* = 4.8 Hz, 1H), 6.38 (t, *J* = 4.8 Hz, 1H), 6.29 (d, *J* = 3.6 Hz, 1H), 6.11 (s, 1H), 3.56 (s, 4H), 3.52 (t, *J* = 5.4 Hz, 4H), 3.44–3.40 (m, 4H), 3.34 (q, *J* = 7.2 Hz, 2H), 3.28 (t, *J* = 7.8 Hz, 2H), 3.28 (q, *J* = 7.2 Hz, 2H), 2.63 (t, *J* = 7.2 Hz, 2H), 2.55 (s, 3H), 2.35 (t, *J* = 7.2 Hz, 2H), 2.25 (s, 3H), 2.24 (t, *J* = 7.2 Hz, 2H), 1.93 (quin, *J* = 7.2 Hz, 2H), 1.14 (t, *J* = 7.2 Hz, 3H), 1.08 (t, *J* = 7.2 Hz, 3H). ^13^C-NMR (CDCl_3_, 150 MHz) δ 172.8, 171.8, 171.6, 160.0, 157.8, 143.7, 135.0, 133.3, 128.3, 123.7, 120.3, 117.4, 70.20, 70.17, 69.9, 69.8, 41.9, 40.1, 39.2, 39.1, 35.8, 35.7, 31.9, 24.8, 21.5, 14.9, 14.3, 13.1, 11.3. IR (film, cm^−1^) ν_max_ 3419, 3299, 3082, 2971, 2931, 2874, 1647, 1606, 1529, 1488, 1439, 1374, 1253, 1175, 1136, 1086, 973, 750, 666. ESI-HRMS *m*/*z* 614.3308 [M + Na]^+^ (calcd. for C_29_H_44_BF_2_N_5_NaO_5_ 614.3301).

## 4. Cell Culture

K562 cells obtained from RIKEN Bio-Resource Center (Tsukuba, Japan) were cultured in RPMI-1640 with L-glutamine and phenol red supplemented with 10% fetal bovine serum, 1% (*w*/*v*) streptomycin/penicillin solution (26253-84, Nacalai tesque, Kyoto, Japan) and 1%NEAA in the presence of 5% CO_2_ in air at 37 °C. A 100 μL aliquot of K562 cells (5000 cells/mL) was added to a 96 well plate (TR5003; True Line, Los Angeles, CA, USA) and incubated in the presence of 5% CO_2_ in air at 37 °C. After 24 h, a 10 μL aliquot of corresponding synthetic probe (concentrations varying in the range of 5 mM–50 nM) was added to a each of the 96 wells and incubated for 24 h. A 10 μL WST-8 solution (mixture of WST-8 and 1-Methoxy PMS, Nacalai tesque, Kyoto, Japan) was added to each well and the incubation continued for 3 h. The visible absorbance at 450 nm and 630 nm as the reference wavelength of each well was quantified using a microplate reader (MTP-310, CORONA Electric Co., Ltd., Tokyo, Japan). HEK293 and HeLa cells obtained from RIKEN Bio-Resource Center (Tsukuba, Japan) were cultured in D-MEM (low glucose) with L-glutamine and phenol red (043-30085; FujifilmWako, Osaka, Japan), supplemented with 10% fetal bovine serum and 1% (*w*/*v*) streptomycin/penicillin solution (26253-84, Nacalai tesque, Japan) in the presence of 5% CO_2_ in air at 37 °C. A 100 μL aliquot of HEK293 cells (10,000 cells/mL) was added to a 96-well plate (TR5003; True Line, Los Angeles, CA, USA) and incubated in the presence of 5% CO_2_ in air at 37 °C. After 24 h, a 10 μL aliquot of corresponding synthetic probe (concentrations varying in the range of 5 mM–50 nM) was added to a each of the 96 wells and incubated for 24 h. A 10 μL WST-8 solution (mixture of WST-8 and 1-Methoxy PMS, Nacalai tesque, Kyoto, Japan) was added to each well and the incubation continued for 4 h. The visible absorbance at 450 nm and 630 nm as the reference wavelength of each well was quantified using a microplate reader (MTP-310, CORONA Electric Co., ltd., Tokyo, Japan).

## Data Availability

The data underlying this article are available in the article and in its online Supplementary Material.

## Supplementary Materials

The following supporting information can be downloaded at https://www.mdpi.com/article/10.3390/molecules30122655/s1, Scheme S1. Synthesis of phenylpyran derivative (**3**), Scheme S2. Synthesis of aniline derivative (**4**), Scheme S3. Synthesis of coumarin derivative (**5**), Scheme S4. Synthesis of BODIPY-FL derivatives (**6**) and (**6a**), Scheme S5. Synthesis of PEG linker (**7b**), Figure S1. Chemical structures of the synthesized compounds. Experimental and ^1^H and ^13^C NMR data.
