# Supplementary material for: Design and Synthesis of Fluorescence-Labeled TAK779 Analogs as Chemical Probes
Source: Molecules. 2025 Jun 19;30(12):2655. doi: 10.3390/molecules30122655 (PMC12195822; doi:10.3390/molecules30122655)

Supporting Information

Design and Synthesis of Fluorescence-Labeled TAK779 Analogs as Chemical Probes

Hiroyuki Konno [1,*], Takuya Saito [1], Taichi Aota [1], Daiki Takanuma [1], Mizuho Okuyama [1] and Chikako Yokoyama [1,2]

[1] Department of Chemistry and Biological Engineering, Graduate School of Science and Engineering, Yamagata University, Yonezawa 992-8510, Yamagata, Japan

[2] Department of Chemistry and Biological Engineering, Graduate School of Engineering, Osaka Metropolitan University, Sumiyoshi-ku, Osaka 558-8585, Japan

* Correspondence: konno@yz.yamagata-u.ac.jp; Tel./Fax: +81-(0)238-26-3131

Table of contents

Scheme S1. Synthesis of phenylpyran derivative (**3**) ……………………………..……3

Scheme S2. Synthesis of aniline derivative (**4**) … …………………..…………………3

Scheme S3. Synthesis of coumarin derivative (**5**)……………………………………..…3

Scheme S4. Synthesis of BODIPY-FL derivatives (**6**) and (**6a**) ……………………..…4

Scheme S5. Synthesis of PEG linker (**7b**) ……………………………………………… 4

Experimental………………………………………………………………………… 7 - 14

^{1}H and ^{13}C NMR data……………………………… ……………………… ………15 - 33

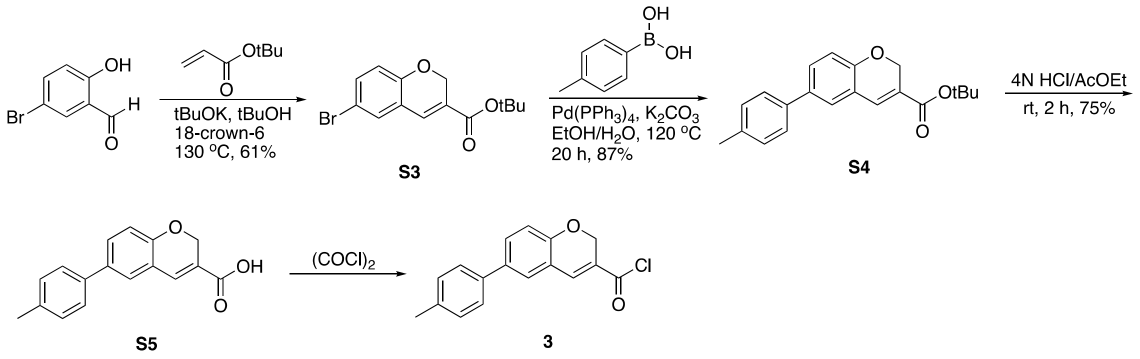

Scheme S1. Synthesis of phenylpyran derivative (**3**)

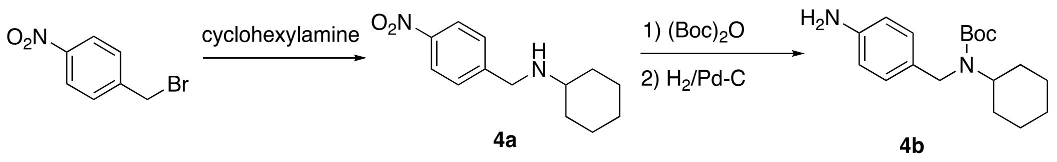

Scheme S2. Synthesis of aniline derivative (**4**)

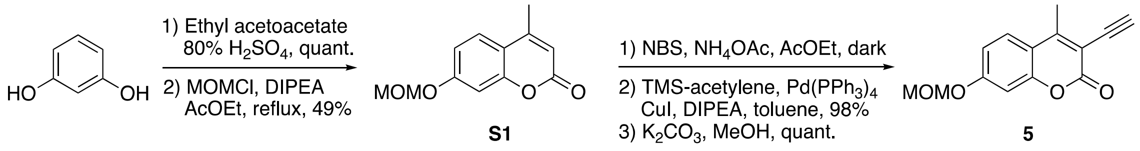

Scheme S3. Synthesis of coumarin derivative (**5**)

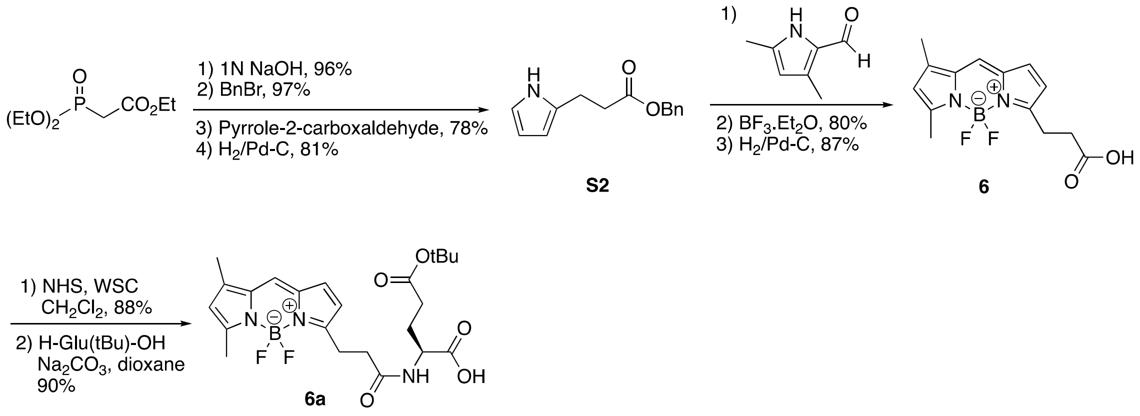

Scheme S4. Synthesis of BODIPY-FL derivatives (**6**) and (**6a**)

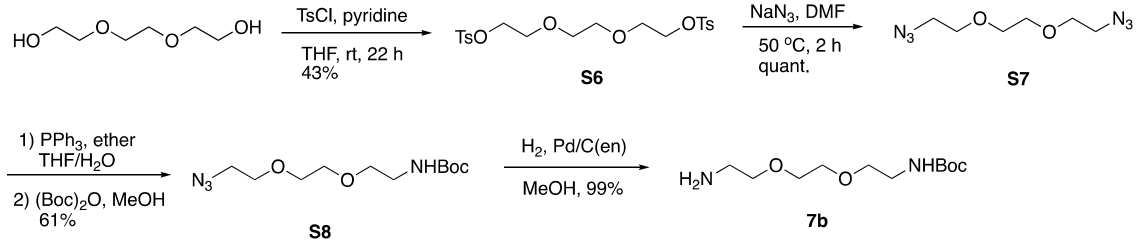

Scheme S5. Synthesis of PEG linker (**7b**)

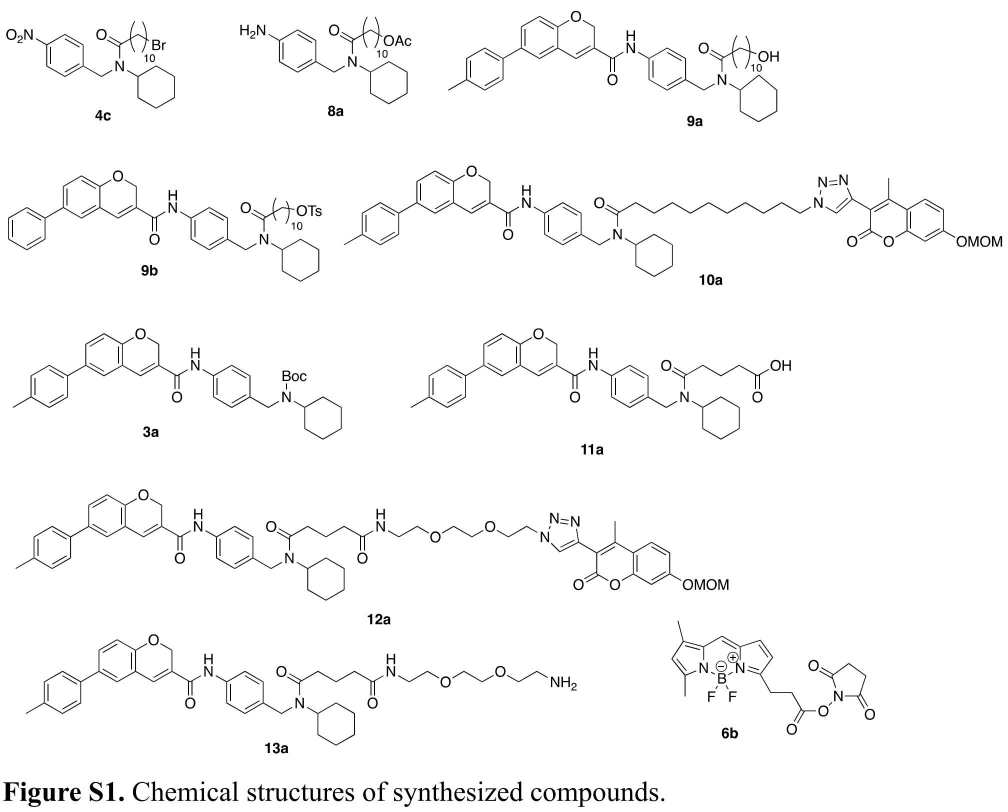

Figure S1. Chemical structures of synthesized compounds.

Experimental

General

All solvents were of reagent grade. THF was distilled from sodium and benzophenone ketyl. CH_2Cl_2 was distilled from CaH_2. All commercial reagents were of the highest purity available. Analytical TLC was performed on silica gel (60 F-254, Plates 0.25 mm). Column chromatography was carried out on silica gel 60 N (Kanto Chemical Co., 40-100 μm). ^{1}H (600, 500 or 400 MHz), and ^{13}C (150, 125 or 100 MHz) NMR spectra were recorded on a JEOL JNM-ECX 600, JNM-ECX 500 or JNM-ECX 400. Chemical shifts are expressed in ppm relative to TMS (0 ppm), $CHCl_3$ (7.26 ppm for ^{1}H and 77.0 ppm for ^{13}C) or DMSO-d_6 (2.50 ppm for ^{1}H and 39.5 ppm for ^{13}C). IR spectra were obtained on a JASCO FT/IR-460 Plus spectrometer. High-resolution mass spectra (HRMS) were obtained on a JEOL The AccuTOF JMS-T100LC (ESI). Melting points were measured on a AS ONE ATM-02. Optical rotations were measured on a JASCO DIP-371. Ultraviolet and visible absorption spectra were obtained on a HITACHI U-1900 or U-2900. Fluorescence spectra were recorded on a JASCO FP-8200 or HITACHI F-2700. Absorbances were measured on a CORONA ELECTRIC MTP-310 Lab.

4-Methylphenylboronic acid

THF (8.0 mL) was added to Mg (296 mg, 12.2 mmol) and I_2 (21 mg, 82.7 μmol) under nitrogen atmosphere. The reaction mixture was heated to reflux and 4-bromotoluene (1.00 mL, 8.13 mmol) was dropwise added to the mixture. The reaction mixture was refluxed for 2 h, then cooled to room temperature. THF (8.0 mL) was added to $B(OMe)_3$ (1.14 mL, 10.2 mmol) under nitrogen atmosphere. To the solution was dropwise added the mixture on ice. The reaction mixture was stirred at room temperature for 18 h, then cooled to room temperature. To the mixture was added 10% H_2SO_4 on ice. The reaction mixture was stirred at room temperature for 3 h, and concentrated in vacuo. The residue was recrystallized with H_2O to give 4-methylphenylboronic acid (490 mg, 3.60 mmol, 44%) as a white powder. ^{1}H-NMR ($CDCl_3$, 600 MHz) δ 8.13 (d, J = 7.8 Hz, 2H), 7.31 (d, J = 7.8 Hz, 2H), 2.44 (s, 3H) ppm. ^{13}C-NMR ($CDCl_3$, 150 MHz) δ 142.9, 135.7, 133.5, 128.7, 21.9 ppm. IR (KBr, cm^{-1}) ν_{max} 3277, 3081, 3049, 3021, 2921, 2861, 1924, 1614, 1402, 1370, 1347, 1310, 1182, 1109, 1082, 1022, 817, 735, 711, 685, 629, 528, 478. m.p. 266-268 °C.

tert-Butyl 6-bromo-2*H*-1-benzopyran-3-carboxylate (S3)

To a solution of 5-bromosalicylaldehyde (2.0 g, 9.95 mmol) in *t*-BuOH (20 mL) were

added *tert*-butyl acrylate (2.17 mL, 13.93 mmol), *t*-BuOK (558 mg, 4.97 mmol) and 18-crown-6 (131 mg, 0.50 mmol). The reaction mixture was stirred at room temperature for 1 h and at 130 °C for 125 h. After cooling to room temperature, the mixture was concentrated in vacuo. The residue was diluted with CH_2Cl_2 and 1 M NaOH. The aqueous layer was extracted with CH_2Cl_2 and the combined organic layer was washed with brine, dried over $MgSO_4$, and concentrated in vacuo to give **S3** (1.90 g, 6.11 mmol, 61%) as a white powder. ^{1}H-NMR ($CDCl_3$, 600 MHz) δ 7.27 (dd, *J* = 9.0, 2.4 Hz, 1H), 7.23 (m, 2H), 6.71 (d, *J* = 9.0 Hz, 1H), 4.94 (d, *J* = 1.2 Hz, 2H), 1.52 (s, 9H). ^{13}C-NMR ($CDCl_3$, 150 MHz) δ 163.4, 154.0, 133.9, 131.1, 130.9, 125.4, 122.9, 117.8, 113.5, 81.5, 64.8, 28.1 ppm. IR (KBr, cm^{-1}) ν_{max} 2979, 2929, 1703, 1644, 1562, 1481, 1370, 1326, 1289, 1260, 1205, 1163, 1091, 999, 930, 849, 821, 720, 615, 464. m.p. 93-94 °C.

***tert*-Butyl 6-(4-methylphenyl)-2*H*-1-benzopyran-3-carboxylate (S4)**
To a solution of **S3** (1.90 g, 6.11 mmol) in toluene/EtOH/H_2O (28 mL, 2:1:1) was added 4-methylphenylboronic acid (996 mg, 7.33 mmol). The reaction mixture was stirred at room temperature under nitrogen atmosphere for 30 min and K_2CO_3 (1.57 g, 11.4 mmol) and $Pd(PPh_3)_4$ (706 mg, 0.61 mmol) were added. The mixture was stirred at 120 °C under nitrogen atmosphere for 20 h, and concentrated in vacuo. The residue was diluted with AcOEt and H_2O. The aqueous layer was extracted with AcOEt and the combined organic layer was washed with brine, dried over $MgSO_4$, and concentrated in vacuo. The residue was purified by column chromatography (hexane/AcOEt = 40:1) to give **S4** (1.72 g, 5.33 mmol, 87%) as a pale-yellow powder. ^{1}H-NMR ($CDCl_3$, 600 MHz) δ 7.43 (m, 4H), 7.34 (d, *J* = 2.4 Hz, 1H), 7.24 (d, *J* = 7.8 Hz, 2H), 6.91 (d, *J* = 8.4 Hz, 1H), 5.00 (d, *J* = 1.2 Hz, 2H), 2.40 (s, 3H), 1.56 (s, 9H). ^{13}C-NMR ($CDCl_3$, 150 MHz) δ 163.8, 154.3, 137.2, 136.7, 134.8, 132.6, 130.0, 129.5, 127.0, 126.4, 124.5, 121.3, 116.2, 81.2, 64.8, 28.1, 21.0. IR (KBr, cm^{-1}) ν_{max} 3016, 2976, 2921, 2852, 1704, 1649, 1494, 1461, 1397, 1370, 1341, 1310, 1279, 1252, 1228, 1160, 1132, 1086, 1004, 912, 849, 808. m.p. 80-81 °C.

6-(4-Methylphenyl)-2*H*-1-benzopyran-3-carboxylic acid (S5)
4 N HCl/AcOEt (27.0 mL) was added to **S4** (1.72 g, 5.33 mmol) on ice. The reaction mixture was stirred at room temperature for 2 h, and concentrated in vacuo. The residue was recrystallized with hexane and AcOEt to give **S5** (1.07 g, 4.02 mmol, 75%) as a pale-yellow powder. ^{1}H-NMR (DMSO-d_6, 500 MHz) δ 7.61 (d, *J* = 2.5 Hz, 1H), 7.50 (m, 4H), 7.22 (d, *J* = 8.0 Hz, 2H), 6.90 (d, *J* = 8.5 Hz, 1H), 4.94 (d, *J* = 1.5 Hz, 2H), 2.31 (s, 3H). ^{13}C-NMR (DMSO-d_6, 125 MHz) δ 165.6, 153.8, 136.4, 136.3, 133.8, 132.4, 129.7, 129.5, 127.0, 126.0, 123.7, 121.2, 116.1, 64.4, 20.7. IR (KBr, cm^{-1}) ν_{max} 3026, 2913, 2862, 1688,

1634, 1607, 1580, 1486, 1441, 1340, 1307, 1265, 1244, 1216, 1185, 1144, 1092, 1020, 1003, 923, 909, 813, 731, 664, 615, 553. m.p. 233-234 °C.

***N*-[(4-Nitrophenyl)methyl]cyclohexanamine (4a)**

To a solution of cyclohexylamine (5.73 mL, 50.0 mmol) in THF (100 mL) was added 4-nitrobenzyl chloride (3.43 g, 20.0 mmol). The reaction mixture was stirred at 50 °C for 6 h. The mixture was cooled to room temperature and TBAI (738 mg, 2.00 mmol) was added. The reaction mixture was stirred at 50 °C for 7 h and cooled to room temperature. The mixture was concentrated in vacuo, diluted with AcOEt and H_2O. The aqueous layer was extracted with AcOEt and the combined organic layer was washed with H_2O and brine, dried over $MgSO_4$, and concentrated in vacuo. The residue was purified by column chromatography (hexane/AcOEt = 1:1-1:3) to give **4a** (4.58 g, 19.5 mmol, 98%) as a brown oil. ^{1}H-NMR ($CDCl_3$, 600 MHz) δ 8.14 (d, *J* = 8.4 Hz, 2H), 7.49 (d, *J* = 8.4 Hz, 2H), 3.90 (s, 2H), 2.44 (tt, *J* = 10.2, 3.6 Hz, 1H), 1.89 (dd, *J* = 12.6, 3.0 Hz, 2H), 1.71 (dt, *J* = 12.6, 3.0 Hz, 2H), 1.59 (dt, *J* = 12.6, 3.0 Hz, 1H), 1.29 (s, 1H), 1.22 (qt, *J* = 12.6, 3.0 Hz, 2H), 1.16 (tt, *J* = 12.6, 3.0 Hz, 1H), 1.09 (qd, *J* = 12.6, 3.0 Hz, 2H). ^{13}C-NMR ($CDCl_3$, 150 MHz) δ 149.1, 146.8, 128.5, 123.5, 56.3, 50.2, 33.5, 26.0, 24.9. IR (film, cm^{-1}) ν_{max}: 3351, 3320, 3203, 3109, 3077, 3057, 2926, 2852, 2707, 2660, 2591, 2449, 2212, 1928, 1800, 1677, 1602, 1520, 1492, 1449, 1344, 1287, 1260, 1240, 1177, 1122, 1108, 1014, 992, 955, 931, 889, 852, 793, 738, 699, 665, 621.

***tert*-Butyl *N*-[(4-aminophenyl)methyl]-*N*-cyclohexylcarbamate (4b)**

To a solution of **4a** (1.49 g, 6.36 mmol) in CH_2Cl_2 (12.5 ml) were added Et_3N (2.66 mL, 19.1 mmol) and $(Boc)_2O$ (1.94 g, 8.28 mmol). The reaction mixture was stirred at room temperature for 4 h, diluted with CH_2Cl_2 and NH_4Cl aq. The aqueous layer was extracted with CH_2Cl_2 and the combined organic layer was washed with brine, dried over $MgSO_4$, and concentrated in vacuo. The residue was dissolved in CH_2Cl_2 (5.0 mL), and ammonia solution (25%, 1.0 mL) was added. The reaction mixture was vigorously stirred at room temperature for 5 min, diluted with CH_2Cl_2 and 1 M HCl. The aqueous layer was extracted with CH_2Cl_2 and the combined organic layer was washed with brine, dried over $MgSO_4$, and concentrated in vacuo to give carbamate (2.02 g, 6.04 mmol, 95%) as a colorless oil. ^{1}H-NMR ($CDCl_3$, 600 MHz) δ 8.14 (d, *J* = 8.4 Hz, 2H), 7.37 (d, *J* = 8.4 Hz, 2H), 4.47-3.72 (m, 3H), 1.74-1.29 (m, 18H), 1.00 (m, 1H). ^{13}C-NMR ($CDCl_3$, 150 MHz) δ 155.2, 148.5, 146.7, 127.4, 126.9, 123.4, 80.1, 56.8, 55.1, 46.0, 31.2, 28.2, 25.8, 25.4. IR (film, cm^{-1}) ν_{max} 2971, 2931, 2856, 1691, 1601, 1522, 1494, 1453, 1403, 1365, 1344, 1280, 1246, 1207, 1166, 1149, 1056, 1104, 1007, 965, 945, 908, 885, 859, 844, 798, 773, 760,

739, 707. ESI-HRMS *m*/*z* 357.1762 $[M+Na]^+$ (calcd. for $C_{18}H_{26}N_2NaO_4$ 357.1790). To a solution of carbamate (2.02 g, 6.04 mmol) in AcOEt (12.0 mL) was added 5% Pt/C (0.20 g). The reaction mixture was vigorously stirred at room temperature under hydrogen atmosphere for 3.5 h, then filtered through celite pad, which was washed with AcOEt. The filtrate was concentrated in vacuo. The residue was purified by column chromatography (hexane/AcOEt = 4:1-2:1) to give **4b** (1.83 g, 6.01 mmol, quant.) as a white powder. ^{1}H-NMR ($CDCl_3$, 600 MHz) δ 7.02 (m, 2H), 6.64 (d, *J* = 8.4 Hz, 2H), 4.23-3.53 (m, 5H), 1.71-1.31 (m, 18H), 0.98 (m, 1H). ^{13}C-NMR ($CDCl_3$, 150 MHz) δ 155.6, 144.1, 130.8, 128.2, 127.6, 115.2, 79.2, 57.1, 55.3, 46.6, 45.9, 31.1, 28.4, 25.9, 25.5. IR (film, cm^{-1}) ν_{max} 3457, 3359, 3227, 3005, 2976, 2931, 2855, 1676, 1626, 1585, 1518, 1452, 1411, 1391, 1365, 1323, 1302, 1279, 1246, 1209, 1166, 1103, 1056, 1005, 957, 908, 895, 879, 840, 817, 756, 665. ESI-HRMS *m*/*z* 327.2042 $[M+Na]^+$ (calcd. for $C_{18}H_{28}N_2NaO_2$ 327.2048). m.p. 97-98 °C.

7-(Methoxymethoxy)-4-methyl-2-oxo-2*H*-1-benzopyran (S1)

To a solution of resorcinol (1.00 g, 9.08 mmol) in 80% H_2SO_4 was dropwise added ethyl acetoacetate (1.28 mL, 10.0 mmol). The reaction mixture was stirred overnight at room temperature, then cooled on ice. The precipitate was collected by filtration, dissolved in acetone, and dried over $MgSO_4$. The filtrate was concentrated in vacuo to give coumarin (1.96 g, quant.) as a white powder. ^{1}H-NMR (DMSO-d_6, 600 MHz) δ 10.5 (s, 1H), 7.52 (d, *J* = 7.8 Hz, 1H), 6.77 (dd, *J* = 9.0, 1.8 Hz, 1H), 6.67 (d, *J* = 2.4 Hz, 1H), 6.07 (s, 1H), 2.32 (s, 3H). ^{13}C-NMR (DMSO-d_6, 150 MHz) δ 161.2, 160.3, 154.8, 153.4, 126.5, 112.8, 112.0, 110.3, 102.2, 18.1. IR (KBr, cm^{-1}) ν_{max} 3163, 1678, 1599, 1452, 1389, 1365, 1334, 1274, 1237, 1214, 1159, 1132, 1067, 981, 867, 847, 747, 583. ESI-HRMS *m*/*z* 177.0569 $[M+H]^+$ (calcd. for $C_{10}H_9O_3$ 177.0552). m.p. 188-190 °C. To a solution of coumarin (5.67 g, 32.2 mmol) in AcOEt were added DIPEA (35.0 mL, 201 mmol) and MOMCl (8.00 mL, 105 mmol). The reaction mixture was refluxed for 5 h, diluted with AcOEt and saturated NH_4Cl aq. The aqueous layer was extracted with AcOEt and the combined organic layer was washed with water and brine, dried over $MgSO_4$, and concentrated in vacuo. The residue was purified by column chromatography (AcOEt), then recrystallized with hexane and AcOEt to give **S1** (3.51 g, 15.9 mmol, 49%) as a white powder. ^{1}H-NMR ($CDCl_3$, 600 MHz) δ 7.43 (d, *J* = 9.0 Hz, 1H), 6.98 (d, *J* = 3.0 Hz, 1H), 6.89 (dd, *J* = 9.0, 2.4 Hz, 1H), 6.13 (s, 1H), 5.21 (s, 2H), 3.47 (s, 3H), 2.38 (s, 3H). ^{13}C-NMR ($CDCl_3$, 150 MHz) δ 161.1, 160.0, 154.9, 152.3, 125.5, 114.4, 113.1, 112.4, 103.8, 94.3, 56.3, 18.6. IR (KBr, cm^{-1}) ν_{max} 2966, 2916, 2852, 1720, 1704, 1611, 1389, 1365, 1342, 1269, 1224, 1187, 1151, 1132, 1086, 1063, 1013, 988, 967, 922, 876, 841, 821. ESI-HRMS *m*/*z* 221.0785

$[M+H]^+$ (calcd. for $C_{12}H_{13}O_4$ 221.0814). m.p. 105-106 °C.

3-Ethynyl-7-(methoxymethoxy)-4-methyl-2-oxo-2*H*- 1-benzopyran (5)

To a solution of **S1** (174 mg, 0.79 mmol) in AcOEt (4.00 mL) were added NBS (211 mg, 1.19 mmol) and NH_4OAc (12 mg, 0.16 mmol). The reaction mixture was stirred at room temperature in the dark for 3 h, diluted with AcOEt and H_2O. The aqueous layer was extracted with AcOEt and the combined organic layer was washed with brine, dried over $MgSO_4$, and concentrated in vacuo. The residue was recrystallized with hexane and AcOEt to give bromide (233 mg, 0.78 mmol, 99%) as a white powder. ^{1}H-NMR ($CDCl_3$, 600 MHz) δ 7.56 (m, 1H), 7.00 (m, 2H), 5.23 (s, 2H), 3.48 (s, 3H), 2.58 (s, 3H). ^{13}C-NMR ($CDCl_3$, 150 MHz) δ 160.2, 157.2, 153.2, 151.0, 126.0, 114.2, 113.8, 110.2, 103.6, 94.4, 56.4, 19.4. IR (KBr, cm^{-1}) ν_{max} 2966, 2921, 2834, 1717, 1617, 1592, 1509, 1384, 1348, 1276, 1249, 1224, 1187, 1156, 1137, 1086, 1068, 1000, 977, 920, 854, 816, 751, 725, 688, 642, 602, 542. To a solution of bromide (100 mg, 0.33 mmol) in toluene (2.50 mL) were added DIPA (235 μL, 1.67 mmol), trimethylsilylacetylene (95 μL, 0.67 mmol), $Pd(PPh_3)_4$ (25 mg, 21.6 μmol) and CuI (8.0 mg, 42.0 μmol). The reaction mixture was stirred at 60 °C under nitrogen atmosphere for 3 h, diluted with AcOEt, saturated NH_4Cl aq. and 4 M HCl. The aqueous layer was extracted with AcOEt and the combined organic layer was washed with brine, dried over $MgSO_4$, and concentrated in vacuo. The residue was purified by column chromatography ($CHCl_3$/AcOEt = 99:1) to give TMS acetylene (94 mg, 0.30 mmol, 89%) as a brown powder. ^{1}H-NMR ($CDCl_3$, 600 MHz) δ 7.53 (m, 1H), 6.98 (m, 2H), 5.22 (s, 2H), 3.48 (s, 3H), 2.58 (s, 3H), 0.28 (s, 9H). ^{13}C-NMR ($CDCl_3$, 150 MHz) δ 160.4, 159.5, 155.0, 153.7, 126.2, 114.1, 113.6, 108.4, 104.7, 103.7, 97.9, 94.3, 56.4, 17.3, -0.1. IR (KBr, cm^{-1}) ν_{max} 2961, 2899, 2825, 2154, 1718, 1618, 1593, 1431, 1386, 1286, 1247, 1212, 1153, 1093, 997, 956, 923, 875, 842, 813, 768, 704. ESI-HRMS *m*/*z* 317.1210 $[M+H]^+$ (calcd. for $C_{17}H_{21}O_4Si$ 317.1209). m.p. 116-117 °C. To a solution of TMS acetylene (0.10 g, 0.32 mmol) in MeOH was added K_2CO_3 (0.14 g, 1.01 mmol). The reaction mixture was stirred at room temperature for 0.3 h, diluted with AcOEt and saturated NH_4Cl aq. The aqueous layer was extracted with AcOEt and the combined organic layer was washed with water and brine, dried over $MgSO_4$, and concentrated in vacuo. The residue was purified by column chromatography (hexane/AcOEt = 3:1) to give **5** (0.23 g, quant.) as a brown powder. ^{1}H-NMR ($CDCl_3$, 600 MHz) δ 7.53 (d, J = 9.0 Hz, 1H), 6.98 (dd, J = 9.0, 2.4 Hz, 1H), 6.96 (d, J = 2.4 Hz, 1H), 5.22 (s, 2H), 3.54 (s, 1H), 3.47 (s, 3H), 2.59 (s, 3H). ^{13}C-NMR ($CDCl_3$, 150 MHz) δ 160.6, 159.8, 155.8, 153.8, 126.2, 113.8, 113.7, 107.4, 103.6, 94.3, 86.4, 56.4, 17.2. IR (KBr, cm^{-1}) ν_{max} 3236, 2975, 2922, 1718, 1615, 1597, 1550, 1507, 1427, 1383, 1361,

1281, 1237, 1212, 1152, 1074, 1028, 980, 944, 922, 848, 817, 769, 721, 699. ESI-HRMS *m*/*z* 245.0805 $[M+H]^+$ (calcd. for $C_{14}H_{13}O_4$ 245.0814). m.p. 107 °C.

Benzyl 3-(1*H*-pyrrol-2-yl)propanoate (S2)

To a solution of ethyl diethylphosphonoacetate (15.0 g, 66.9 mmol) in MeOH (90 mL) was added 1 M NaOH (90 mL) on ice. The reaction mixture was stirred at room temperature for 1 h, and concentrated in vacuo. The residue was adjusted to pH 2 with 4 M HCl. The aqueous layer was extracted with AcOEt and the combined organic layer was washed with brine, dried over $MgSO_4$, and concentrated in vacuo to give diethylphosphonoacetic acid (12.6 g, 64.2 mmol, 96%) as a colorless oil. ^{1}H-NMR ($CDCl_3$, 600 MHz) δ 10.40 (s, 1H), 4.17 (m, 4H), 2.98 (m, 2H), 1.31 (t, J = 7.2 Hz, 6H). ^{13}C-NMR ($CDCl_3$, 125 MHz) δ 168.0 (d, J = 6.0 Hz), 63.2 (d, J = 7.1 Hz), 34.0 (d, J = 135 Hz), 16.1 (d, J = 7.3 Hz). IR (film, cm^{-1}) ν_{max} 3409, 2987, 2931, 2625, 2519, 1729, 1479, 1394, 1237, 1119, 1023, 977, 905, 843, 782, 666, 602. To a solution of diethylphosphonoacetic acid (12.3 g, 62.7 mmol) in DMF (120 mL) was added K_2CO_3 (8.67 g, 62.7 mmol) on ice. The reaction mixture was stirred at room temperature for 30 min, then BnBr (8.94 mL, 75.3 mmol) was dropwise added on ice. The reaction mixture was stirred at room temperature for 21 h, diluted with hexane/AcOEt (3:1) and H_2O. The aqueous layer was extracted with hexane/AcOEt (3:1) and the combined organic layer was washed with brine, dried over $MgSO_4$, and concentrated in vacuo. The residue was diluted with AcOEt and H_2O. The aqueous layer was extracted with AcOEt and the combined organic layer was washed with brine, dried over $MgSO_4$, and concentrated in vacuo. The residue was purified by column chromatography (hexane/AcOEt = 1:1) to give benzyl diethylphosphonoacetate (17.5 g, 61.1 mmol, 97%) as a colorless oil. ^{1}H-NMR ($CDCl_3$, 500 MHz) δ 7.32 (m, 5H), 5.14 (s, 2H), 4.09 (dt, J = 8.5, 7.0 Hz, 4H), 2.98 (d, J = 21.5 Hz, 2H), 1.26 (t, J = 7.0 Hz) ppm. ^{13}C-NMR ($CDCl_3$, 125 MHz) δ 165.5 (d, J = 6.0 Hz), 135.2, 128.4, 128.25, 128.21, 67.1, 62.6 (d, J = 7.3 Hz), 34.2 (d, J = 134 Hz), 16.1 (d, J = 6.0 Hz). IR (film, cm^{-1}) ν_{max} 3470, 3065, 3033, 2984, 2934, 2904, 1737, 1643, 1498, 1456, 1394, 1373, 1271, 1210, 1163, 1116, 1054, 1025, 972, 888, 838, 781, 750, 699, 665, 580. To a solution of benzyl diethylphosphonoacetate (8.20 g, 28.6 mmol) in THF (55.0 mL) was added NaH (60% in oil, 1.26 g, 28.9 mmol) on ice. The reaction mixture was stirred at room temperature for 10 min, then a solution of pyrrole-2-carbaldehyde (2.62 g, 27.5 mmol) in THF (14.0 mL) was dropwise added to the mixture on ice. The reaction mixture was stirred at room temperature for 1.5 h, diluted with 5% citric acid aq. (10.0 mL) on ice. The aqueous layer was extracted with AcOEt and the combined organic layer was washed successively with water and brine, dried over $MgSO_4$,

and concentrated in vacuo. The residue was purified by column chromatography (hexane/AcOEt = 6:1) to give benzyl 3-(1*H*-pyrrol-2-yl)-2-propenoate (4.88 g, 21.5 mmol, 78%) as a white powder. ^{1}H-NMR ($CDCl_3$, 600 MHz) δ 9.07 (s, 1H), 7.64 (d, *J* = 15.6 Hz, 1H), 7.27 (m, 5H), 6.92 (m, 1H), 6.58 (m, 1H), 6.29 (m, 1H), 6.13 (d, *J* = 15.6 Hz, 1H). ^{13}C-NMR ($CDCl_3$, 150 MHz) δ 167.7, 136.2, 134.9, 128.5, 128.3, 128.12, 128.07, 122.7, 114.6, 110.9, 110.6, 66.1. IR (film, cm^{-1}) ν_{max} 3335, 3111, 3087, 3064, 3033, 2954, 2886, 1684, 1624, 1547, 1497, 1454, 1416, 1375, 1322, 1289, 1263, 1216, 1169, 1126, 1096, 1036, 974, 907, 881, 849, 815, 736, 697, 666. To a solution of benzyl 3-(1*H*-pyrrol-2-yl)-2-propenoate (8.70 g, 38.3 mmol) in MeOH (132 mL) were added 10% Pd/C (0.79 g) and Ph_2S (25 μL, 0.15 mmol). The reaction mixture was vigorously stirred at room temperature under hydrogen atmosphere for 3.5 h, then 10% Pd/C (0.40 g) was added. The mixture was vigorously stirred at room temperature under hydrogen atmosphere for 2 h, filtered through celite pad, which was washed with MeOH. The filtrate was concentrated in vacuo. The residue was purified by column chromatography (hexane/AcOEt = 10:1) to give **S2** (7.14 g, 31.1 mmol, 81%) as a colorless oil. ^{1}H-NMR ($CDCl_3$, 600 MHz) δ 8.51 (s, 1H), 7.41 (m, 5H), 6.69 (m, 1H), 6.17 (q, *J* = 3.0 Hz, 1H), 5.98 (s, 1H), 5.20 (s, 2H), 2.98 (t, *J* = 6.6 Hz, 2H), 2.74 (t, *J* = 6.6 Hz, 2H). ^{13}C-NMR ($CDCl_3$, 150 MHz) δ 173.7, 135.6, 130.7, 128.5, 128.2, 128.1, 116.7, 107.9, 105.4, 66.4, 34.5, 22.5. IR (film, cm^{-1}) ν_{max} 3389, 3093, 3064, 3033, 2957, 2921, 1955, 1877, 1727, 1607, 1570, 1497, 1455, 1429, 1384, 1353, 1310, 1287, 1255, 1214, 1192, 1156, 1118, 1093, 1028, 999, 962, 911, 884, 793, 718, 665, 440.

BODIPY FL propionic acid (6)

To a solution of **S2** (4.82 g, 21.0 mmol) in $PhCF_3$ (75.0 mL) were added 3,5-dimethylpyrrole-2-carbaldehyde (2.59 g, 21.0 mmol) and $POCl_3$ (1.97 mL, 21.7 mmol) on ice. The reaction mixture was stirred at room temperature for 1 h, then BF_3•Et_2O (10.6 mL, 84.1 mmol) and DIPEA (14.7 mL, 84.1 mmol) were added on ice. The reaction mixture was stirred at room temperature for 12 h, diluted with AcOEt and 5% $NaHCO_3$ aq. (50.0 mL) on ice, then filtered through celite pad, which was washed with AcOEt. The aqueous layer was extracted with AcOEt and the combined organic layer was washed with brine, dried over $MgSO_4$, and concentrated in vacuo. The residue was purified by column chromatography (hexane/AcOEt = 10:1 - 4:1) to give BODIPY FL benzyl propionate (6.43 g, 16.8 mmol, 80%) as a red oil. ^{1}H-NMR ($CDCl_3$, 500 MHz) δ 7.36-7.30 (m, 5H), 7.04 (s, 1H), 6.83 (d, *J* = 4.0 Hz, 1H), 6.22 (d, *J* = 4.0 Hz, 1H), 6.07 (s, 1H), 5.15 (s, 2H), 3.34 (t, *J* = 7.5 Hz, 2H), 2.83 (t, *J* = 7.5 Hz, 2H), 2.55 (s, 3H), 2.19 (s, 3H). ^{13}C-NMR ($CDCl_3$, 125 MHz) δ 172.1, 160.1, 156.6, 143.8, 135.7, 135.0, 133.1, 128.3,

128.04, 127.99, 127.9, 123.7, 120.2, 116.4, 66.2, 33.2, 23.7, 14.7, 11.0. IR (film, cm^{-1}) ν_{max} 3065, 3033, 2954, 1735, 1606, 1529, 1488, 1445, 1381, 1358, 1317, 1251, 1169, 1135, 1085, 1059, 973, 934, 906, 815, 792, 751, 698, 667. To a solution of BODIPY FL benzyl propionate (10.5 g, 27.5 mmol) in MeOH (230 mL) was added 10% Pd/C (0.55 g). The reaction mixture was vigorously stirred at room temperature under hydrogen atmosphere for 3.5 h, filtered through celite pad, which was washed with MeOH. The filtrate was concentrated in vacuo to give **6** (7.42 g, 25.4 mmol, 92%) as a red powder. ^{1}H-NMR (DMSO-d_6, 600 MHz) δ 12.32 (s, 1H), 7.69 (s, 1H), 7.08 (d, *J* = 3.6 Hz, 1H), 6.38 (d, *J* = 3.6 Hz, 1H), 6.30 (s, 1H), 3.08 (t, *J* = 7.8 Hz, 2H), 2.65 (t, *J* = 7.8 Hz, 2H), 2.47 (s, 3H), 2.25 (s, 3H). ^{13}C-NMR (DMSO-d_6, 150 MHz) δ173.5, 159.5, 156.9, 144.3, 134.6, 133.0, 128.8, 125.4, 120.4, 116.5, 32.3, 23.5, 14.5, 11.0. IR (KBr, cm^{-1}) ν_{max} 2921, 1704, 1604, 1525, 1489, 1439, 1424, 1365, 1319, 1292, 1250, 1214, 1196, 1169, 1137, 1104, 1077, 1049, 1013, 972, 926, 903, 821, 798, 757, 734, 711, 670, 647, 592, 532, 492.

12-(2-{[(1*S*)-4-(*tert*-Butoxy)-1-carboxy-4-oxobutyl]carbamoyl}ethyl)-2,2-difluoro-4,6-dimethyl-1λ^5,3-diaza-2-boratricyclo[7.3.0.0^{3,7}]dodeca-1(12),4,6,8,10-pentaen-1-ylium-2-uide (6a)

To a solution of **6** (15 mg, 51.4 μmol) in CH_2Cl_2 (300 μL) was added WSC•HCl (20 mg, 104 μmol). The reaction mixture was stirred at room temperature for 15 min, then NHS (12 mg, 104 μmol) was added to the mixture. The mixture was stirred at room temperature for 1 h, and concentrated in vacuo. The residue was purified by column chromatography ($CHCl_3$) to give NHS ester (14 mg, 36.0 μmol, 70%) as a red oil. ^{1}H-NMR ($CDCl_3$, 600 MHz) δ 7.10 (s, 1H), 6.89 (d, *J* = 4.2 Hz, 1H), 6.33 (d, *J* = 4.2 Hz, 1H), 6.12 (s, 1H), 3.38 (t, *J* = 7.2 Hz, 2H), 3.08 (t, *J* = 7.2 Hz, 2H), 2.84 (m, 4H), 2.57 (s, 3H), 2.25 (s, 3H). ^{13}C-NMR ($CDCl_3$, 150 MHz) δ 169.0, 167.8, 161.2, 154.7, 144.4, 135.5, 133.3, 127.9, 124.0, 120.7, 116.7, 30.3, 25.6, 23.3, 15.0, 11.3. IR (film, cm^{-1}) ν_{max} 3117, 3024, 2925, 2854, 1816, 1784, 1739, 1607, 1529, 1488, 1446, 1370, 1316, 1254, 1202, 1176, 1140, 1085, 1065, 1001, 973, 928, 902, 841, 813, 756, 670, 649, 590. ESI-HRMS *m*/*z* 412.1245 $[M+Na]^+$ (calcd. for $C_{18}H_{18}BF_2N_3NaO_4$ 412.1256). To a solution of H-Glu(tBu)-OH (370 mg, 1.82 mmol) in dioxane (12.0 mL) and H_2O (4.0 mL) was dropwise added a solution of NHS ester (400 mg, 1.03 mmol) in dioxane (3.0 mL) on ice. The reaction mixture was stirred at room temperature for 10 min, then Na_2CO_3 (193 mg, 1.82 mmol) was added to the mixture on ice. The mixture was stirred at room temperature for 1.5 h, then adjusted to pH 2 with 1 M HCl on ice. The aqueous layer was extracted with AcOEt and the combined organic layer was washed with brine, dried over $MgSO_4$, and concentrated in vacuo. The residue was purified by column chromatography ($CHCl_3$/MeOH = 95:5-

90:10) to give **6a** (440 mg, 0.92 mmol, 90%) as a red oil. ^{1}H-NMR ($CDCl_3$, 600 MHz) δ 9.89 (s, 1H), 7.05 (s, 1H), 6.82 (d, *J* = 4.2 Hz, 1H), 6.75 (d, *J* = 7.2 Hz, 1H), 6.24 (d, *J* = 7.2 Hz, 1H), 6.07 (s, 1H), 4.52 (td, *J* = 7.8, 4.8 Hz, 1H), 3.25 (t, *J* = 7.8 Hz, 2H), 2.68 (t, *J* = 7.2 Hz, 2H), 2.52 (s, 3H), 2.26 (m, 2H), 2.20 (s, 3H), 2.12 (m, 1H), 1.91 (m, 1H), 1.41 (s, 9H). ^{13}C-NMR ($CDCl_3$, 150 MHz) δ 174.6, 172.7, 172.5, 160.1, 156.8, 143.8, 135.0, 133.3, 128.3, 123.8, 120.3, 117.3, 80.9, 51.9, 35.2, 31.4, 27.9, 26.8, 24.4, 14.8, 11.2. IR (film, cm^{-1}) ν_{max} 3391, 3321, 3069, 2979, 2932, 1728, 1662, 1607, 1529, 1488, 1445, 1408, 1368, 1331, 1308, 1254, 1174, 1153, 1138, 1086, 1065, 1031, 999, 973, 911, 846, 809, 782, 733, 670, 649, 590. ESI-HRMS *m*/*z* 500.2158 $[M+Na]^+$ (calcd. for $C_{23}H_{30}BF_2N_3NaO_5$ 500.2144). $[\alpha]_D^{28}$ -323.0 (*c* 0.1, $CHCl_3$).

1,2-Bis(2-azidoethoxy)ethane (S7)

To a solution of **S6** (3.19 g, 6.96 mmol) in DMF (9.0 mL) was added NaN_3 (2.72 g, 41.8 mmol). The reaction mixture was stirred at 50 °C for 2 h, diluted with hexane/AcOEt (4:1) and H_2O. The aqueous layer was extracted with hexane/AcOEt (4:1) and the combined organic layer was washed with brine, dried over $MgSO_4$, and concentrated in vacuo. The residue was purified by column chromatography (hexane/AcOEt = 4:1) to give **S7** (1.40 g, quant.) as a colorless oil. ^{1}H-NMR ($CDCl_3$, 600 MHz) δ 3.67 (t, *J* = 4.8 Hz, 4H), 3.66 (s, 4H), 3.37 (t, *J* = 4.8 Hz, 4H). ^{13}C-NMR ($CDCl_3$, 150 MHz) δ 70.6, 70.0, 50.6. IR (film, cm^{-1}) ν_{max} 2925, 2869, 2525, 2107, 1442, 1345, 1286, 1123, 1036, 990, 933, 853, 822, 646, 556, 502.

1,1-Dimethylethyl-*N*-2-[2-(2-azidoethoxy)ethoxy]ethylcarbamate (S8)

To a solution of **S7** (694 mg, 3.47 mmol) in ether (5.0 mL), THF (5.0 mL) and 1 M HCl (5.0 mL) was dropwise added a solution of PPh_3 (909 mg, 3.47 mmol) in Et_2O (5.0 mL) over 3 h. The reaction mixture was stirred at room temperature for 15 h, diluted with Et_2O and 4 M HCl. The aqueous layer was washed with Et_2O and adjusted to pH 14 with 5 M NaOH on ice. The aqueous layer was extracted with CH_2Cl_2. The combined organic layer was dried over $MgSO_4$, and concentrated in vacuo to give **7a** as a yellow oil. ^{1}H-NMR ($CDCl_3$, 400 MHz) δ 3.54-3.47 (m, 6H), 3.36 (t, *J* = 5.2 Hz, 2H), 3.23 (t, *J* = 5.2 Hz, 2H), 2.70 (t, *J* = 5.2 Hz, 2H), 1.45 (s, 2H). ^{13}C-NMR ($CDCl_3$, 100 MHz) δ 73.0, 70.5, 70.1, 69.9, 50.5, 41.5. IR (film, cm^{-1}) ν_{max} 3369, 2926, 2871, 2108, 1645, 1575, 1474, 1347, 1303, 1119, 931, 850, 821, 642, 556. To a solution of the residue in MeOH (7.0 mL) was added $(Boc)_2O$ (2.27 g, 10.4 mmol). The reaction mixture was stirred at room temperature for 4 h, and concentrated in vacuo. The residue was purified by column chromatography (hexane/AcOEt = 2:1) to give **S8** (576 mg, 2.10 mmol, 61%, 2 steps) as a colorless oil.

^{1}H-NMR ($CDCl_3$, 400 MHz) δ 5.02 (s, 1H), 3.66 (t, *J* = 5.2 Hz, 2H), 3.62 (m, 4H), 3.53 (t, *J* = 5.2 Hz, 2H), 3.38 (t, *J* = 5.2 Hz, 2H), 3.30 (m, 2H), 1.42 (s, 9H). ^{13}C-NMR ($CDCl_3$, 150 MHz) δ 155.9, 79.1, 70.5, 70.2, 70.1, 70.0, 50.6, 40.2, 28.3. IR (film, cm^{-1}) ν_{max} 3360, 2977, 2930, 2870, 2109, 1713, 1514, 1455, 1391, 1366, 1278, 1251, 1173, 1122, 1041, 1001, 970, 931, 864, 827, 781, 758, 646, 557.

***tert*-Butyl *N*-{2-[2-(2-aminoethoxy)ethoxy]ethyl}carbamate (7b)**

To a solution of **S8** (631 mg, 2.30 mmol) in MeOH (11.5 mL) was added 5% Pd/C(en) (63 mg). The reaction mixture was vigorously stirred at room temperature under hydrogen atmosphere for 30 min, filtered through celite pad, which was washed with MeOH. The filtrate was concentrated in vacuo to give **7b** (564 mg, 2.27 mmol, 99%) as a colorless oil. ^{1}H-NMR ($CDCl_3$, 600 MHz) δ 5.25 (s, 1H), 3.53 (s, 4H), 3.46 (t, *J* = 4.8 Hz, 2H), 3.43 (t, *J* = 4.8 Hz, 2H), 3.23 (m, 2H), 2.79 (t, *J* = 4.8 Hz, 2H), 1.91 (s, 2H), 1.35 (s, 9H). ^{13}C-NMR ($CDCl_3$, 150 MHz) δ 155.9, 78.9, 73.1, 70.02, 69.97, 41.4, 40.1, 28.2. IR (film, cm^{-1}) ν_{max} 3354, 2975, 2932, 2869, 1695, 1516, 1454, 1391, 1365, 1276, 1250, 1172, 1114, 1039, 1000, 969, 865, 819, 781, 758.

^{1}H-NMR of **4c**

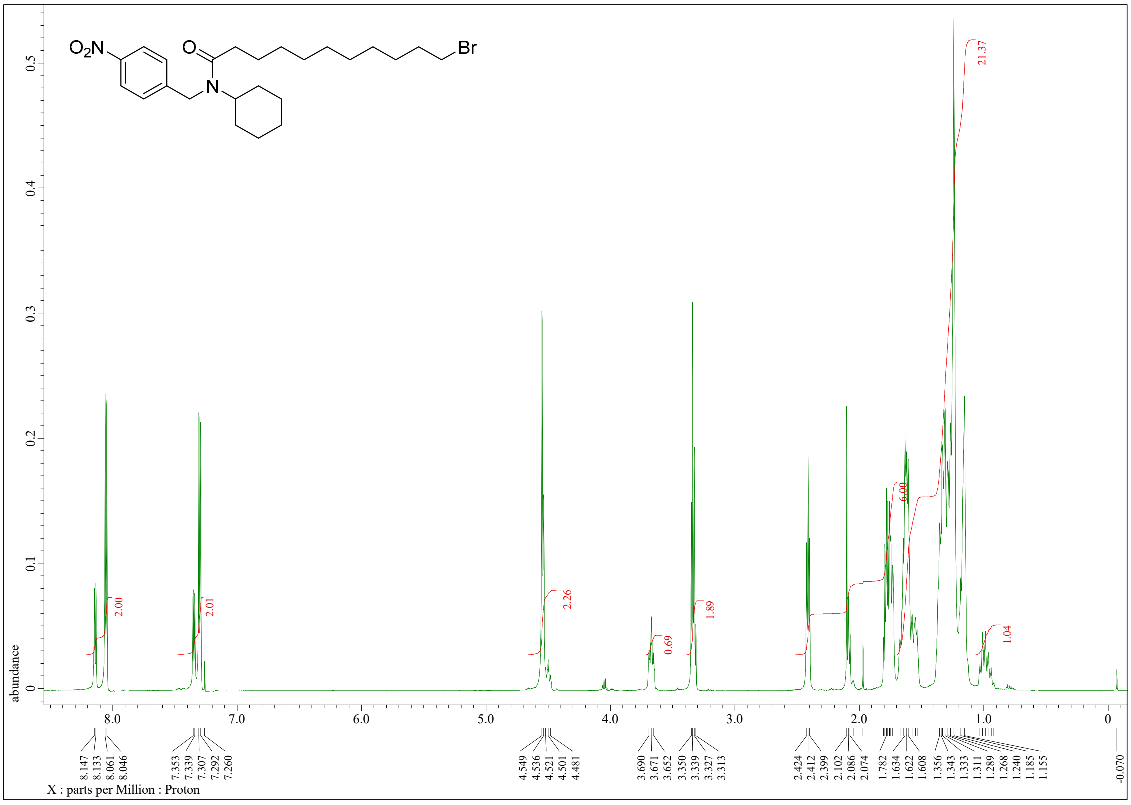

^{13}C-NMR of **4c**

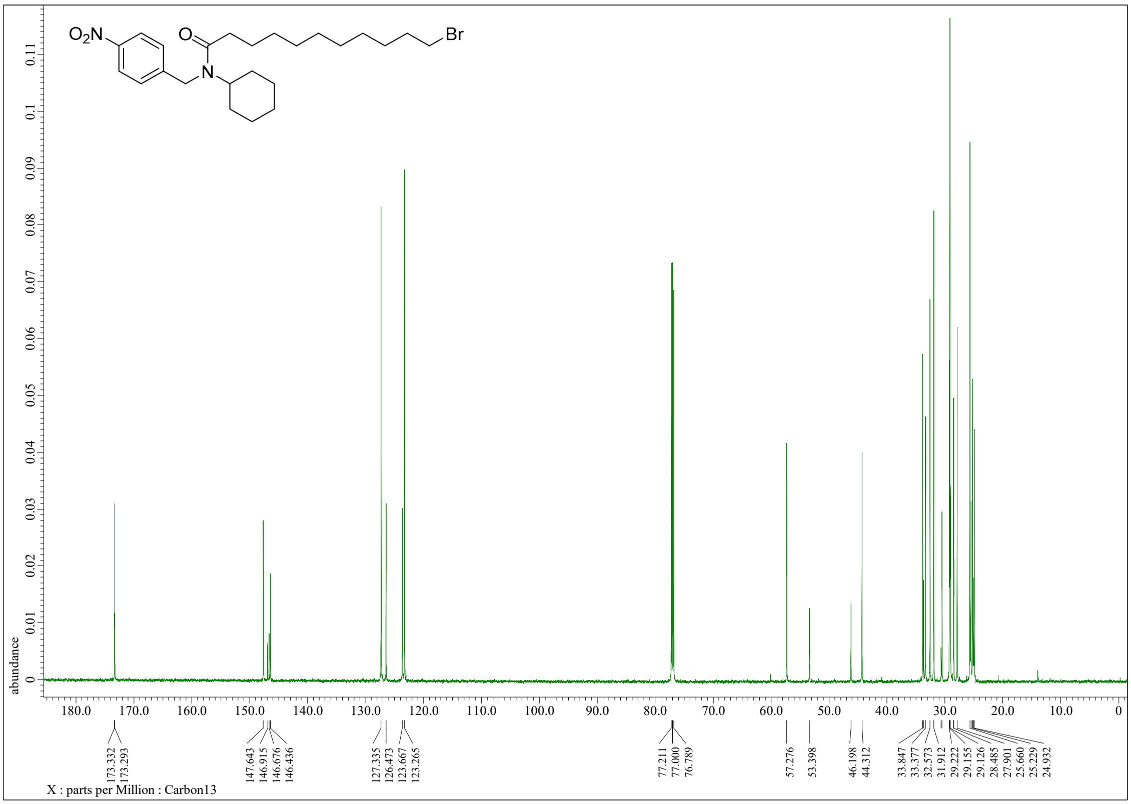

^{1}H-NMR of **8**

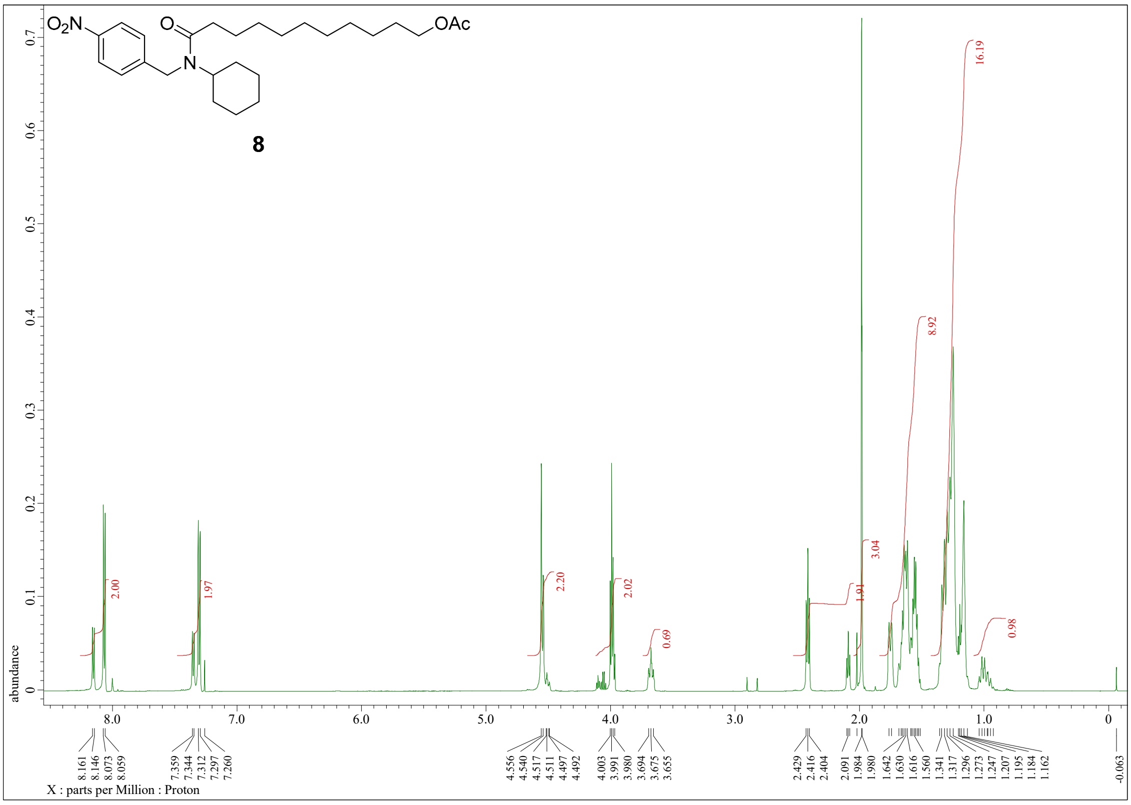

^{13}C-NMR of **8**

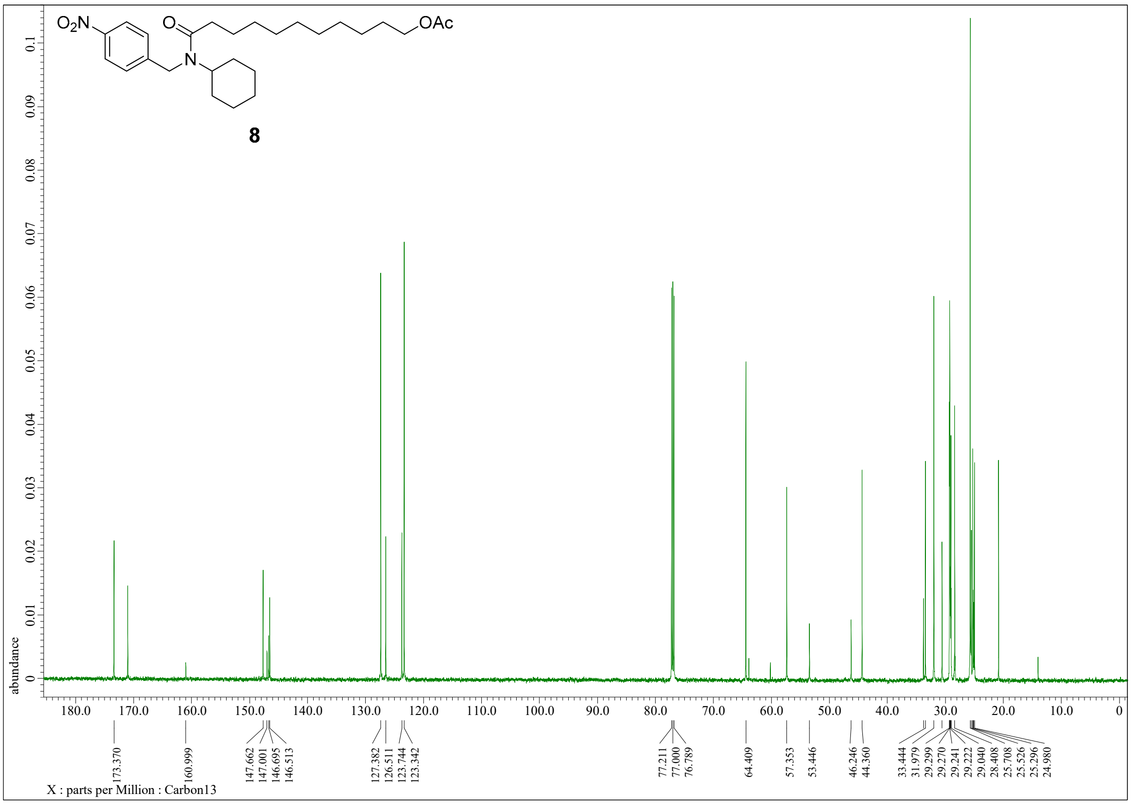

^{1}H-NMR of **8a**

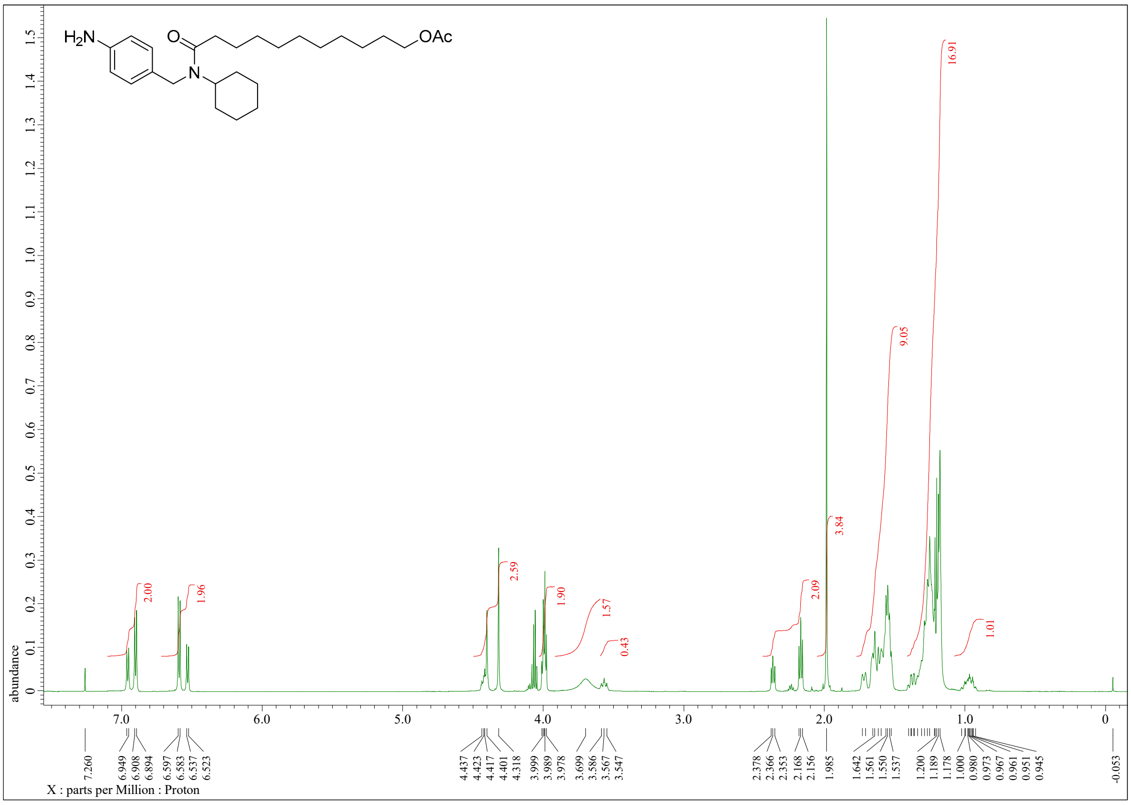

^{13}C-NMR of **8a**

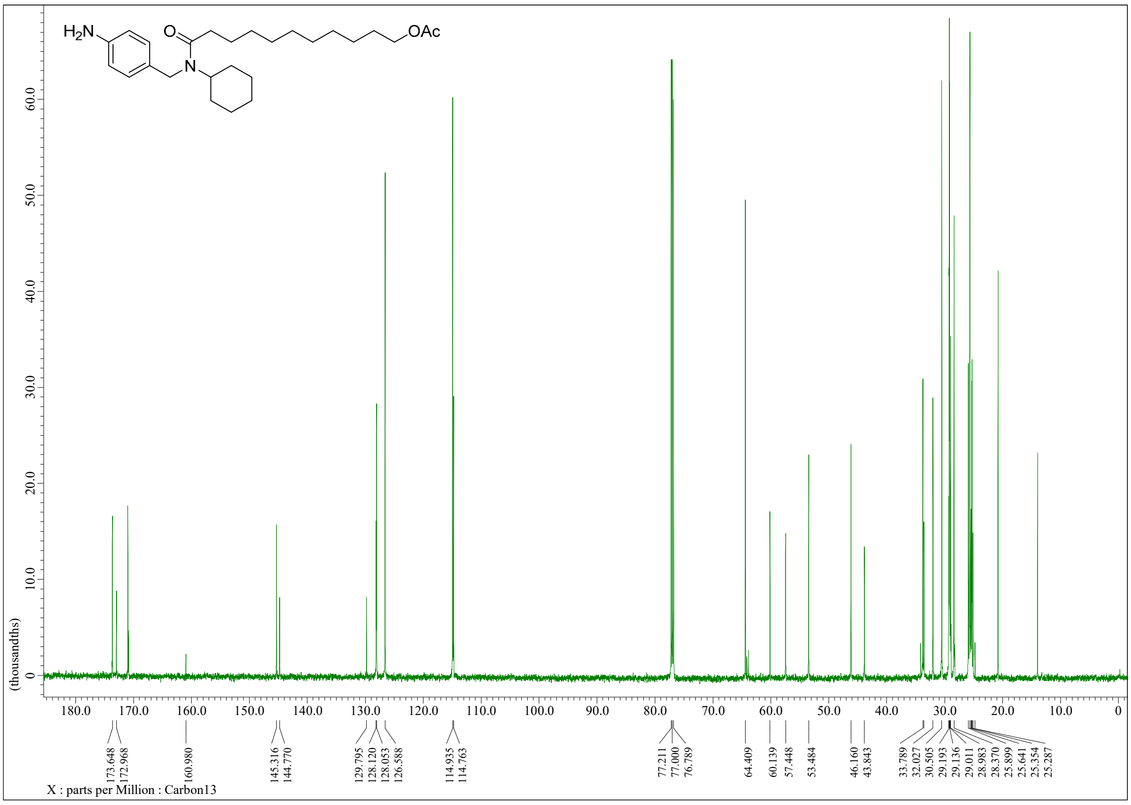

^{1}H-NMR of **9**

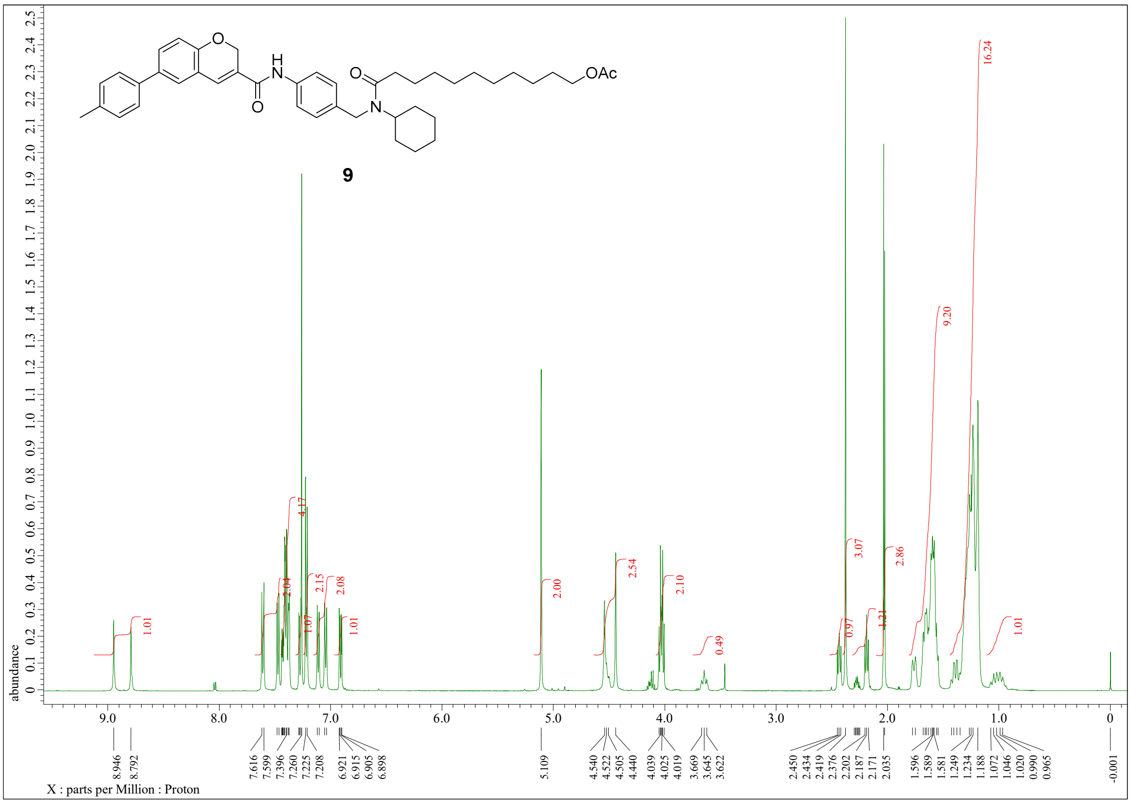

^{13}C-NMR of **9**

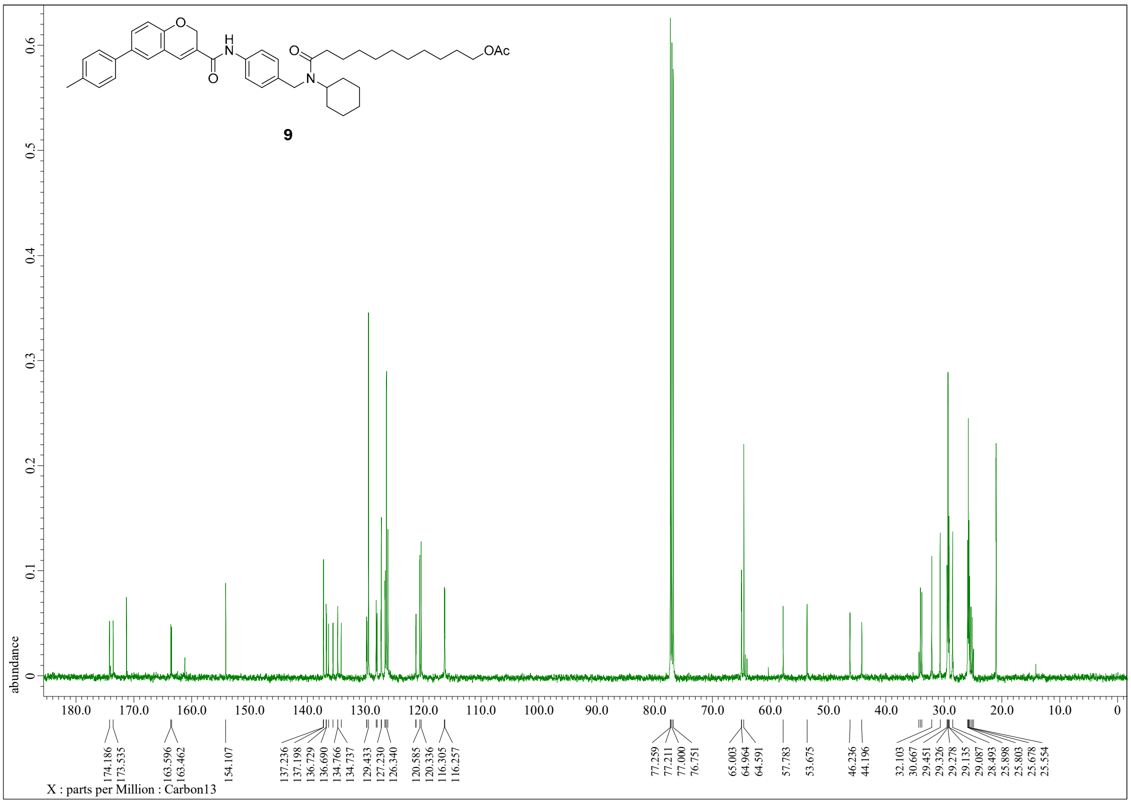

^{1}H-NMR of **9a**

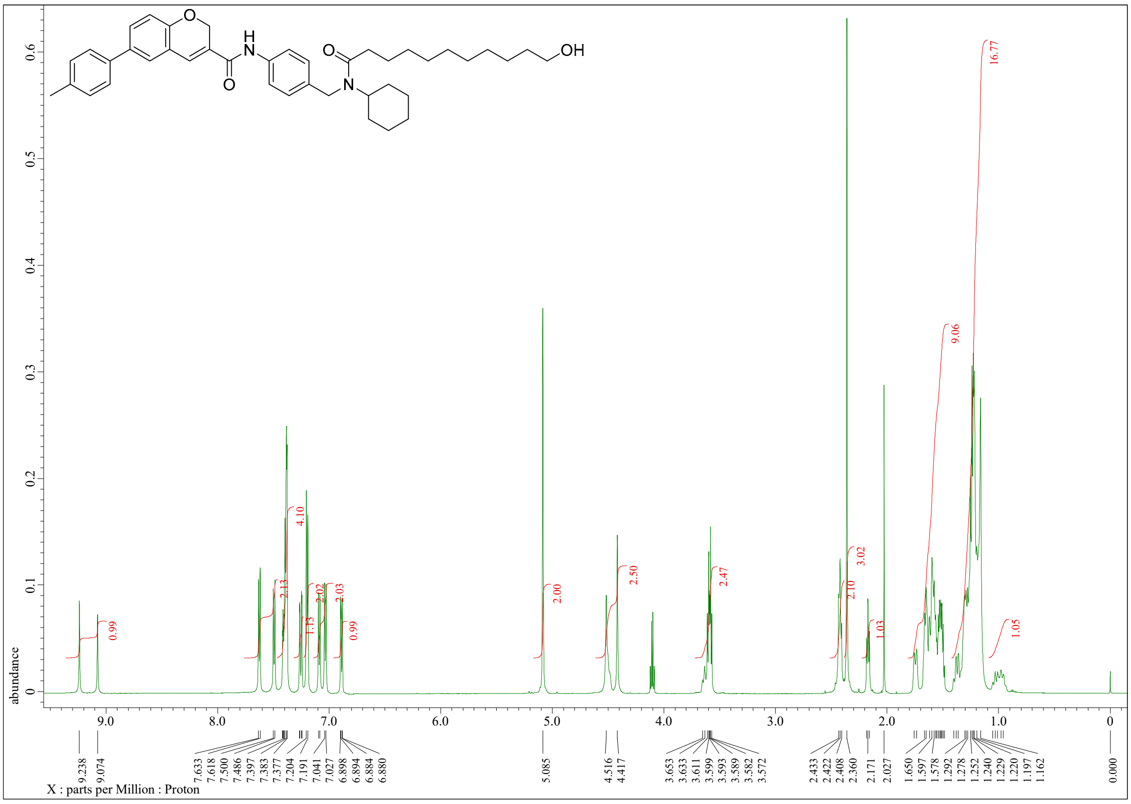

^{13}C-NMR of **9a**

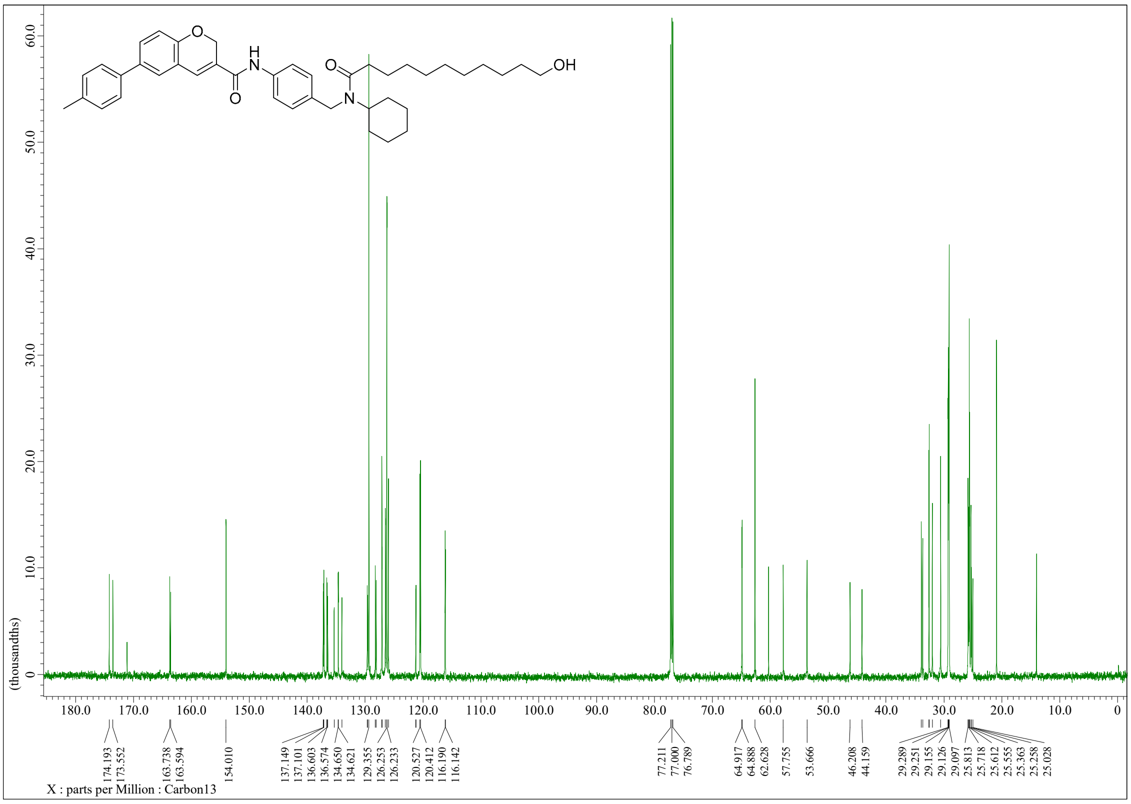

^{1}H-NMR of **9b**

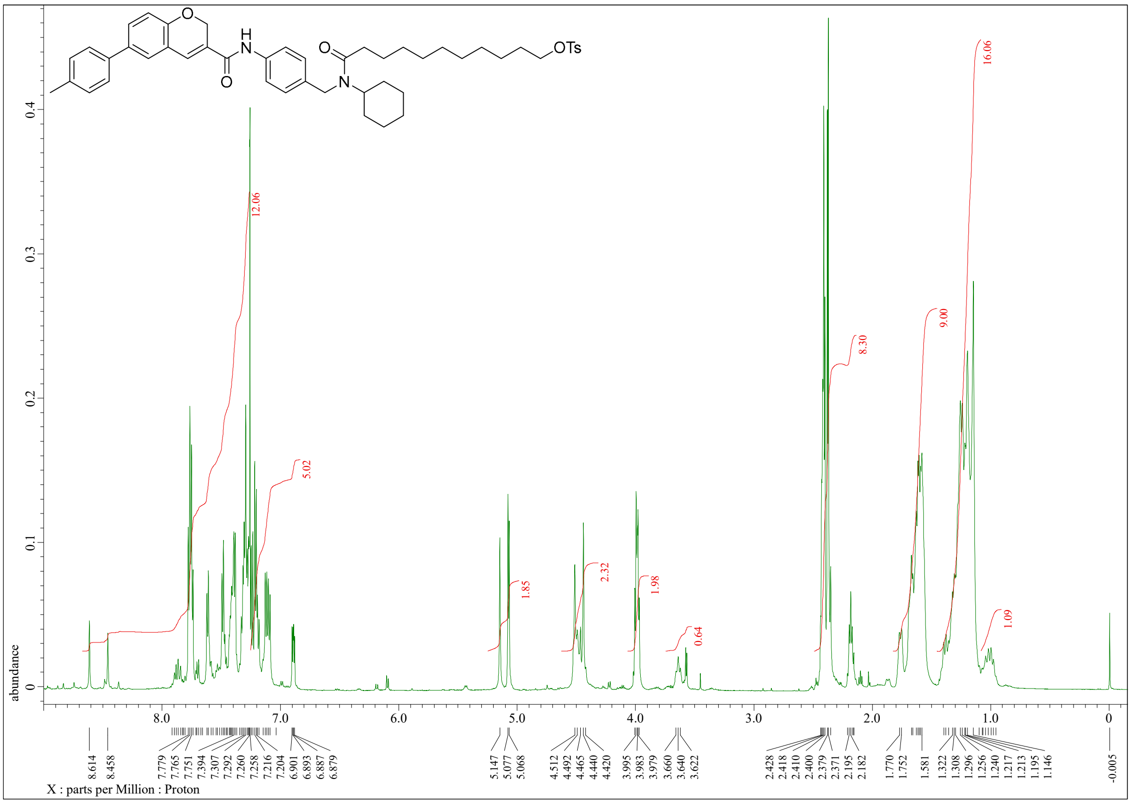

^{13}C-NMR of **9b**

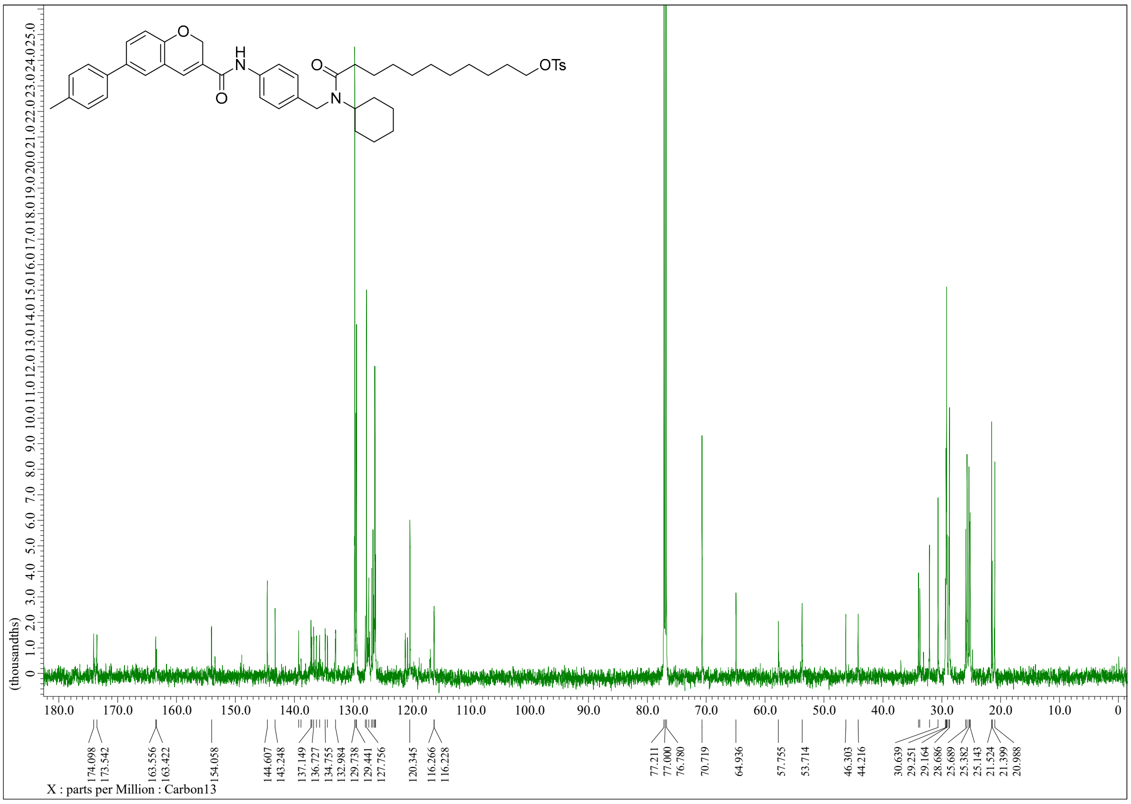

^{1}H-NMR of **10**

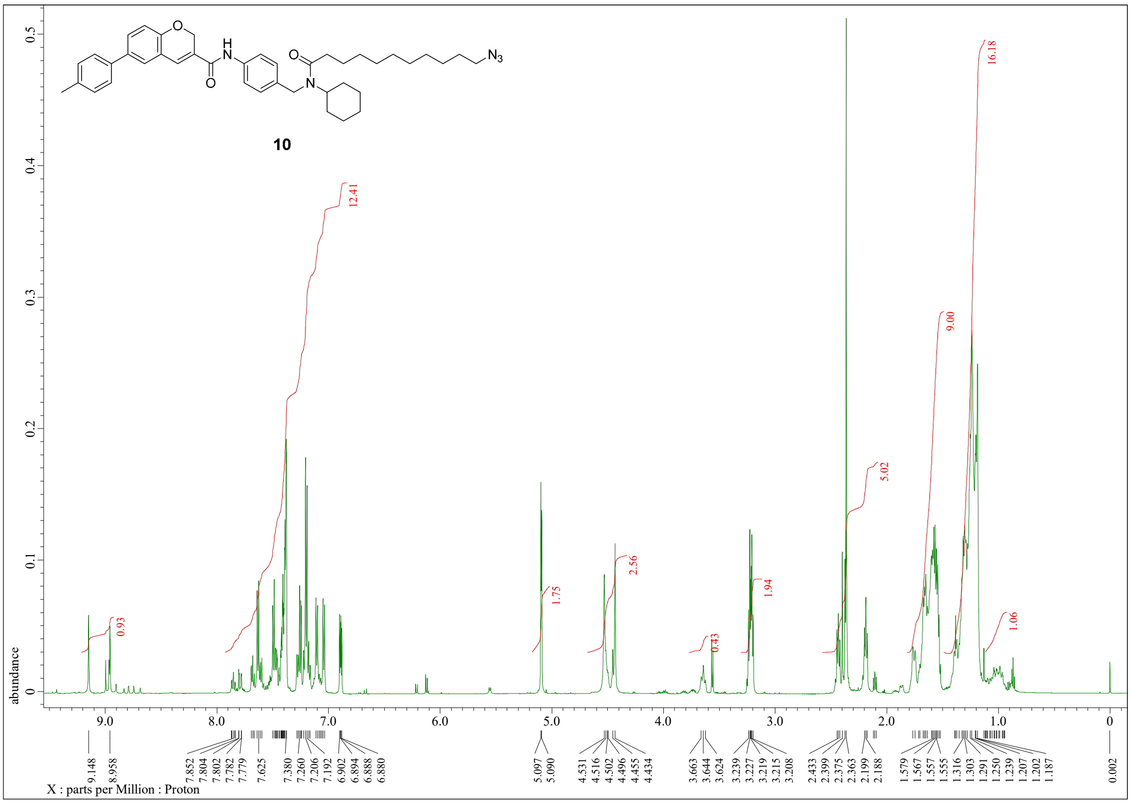

^{13}C-NMR of **10**

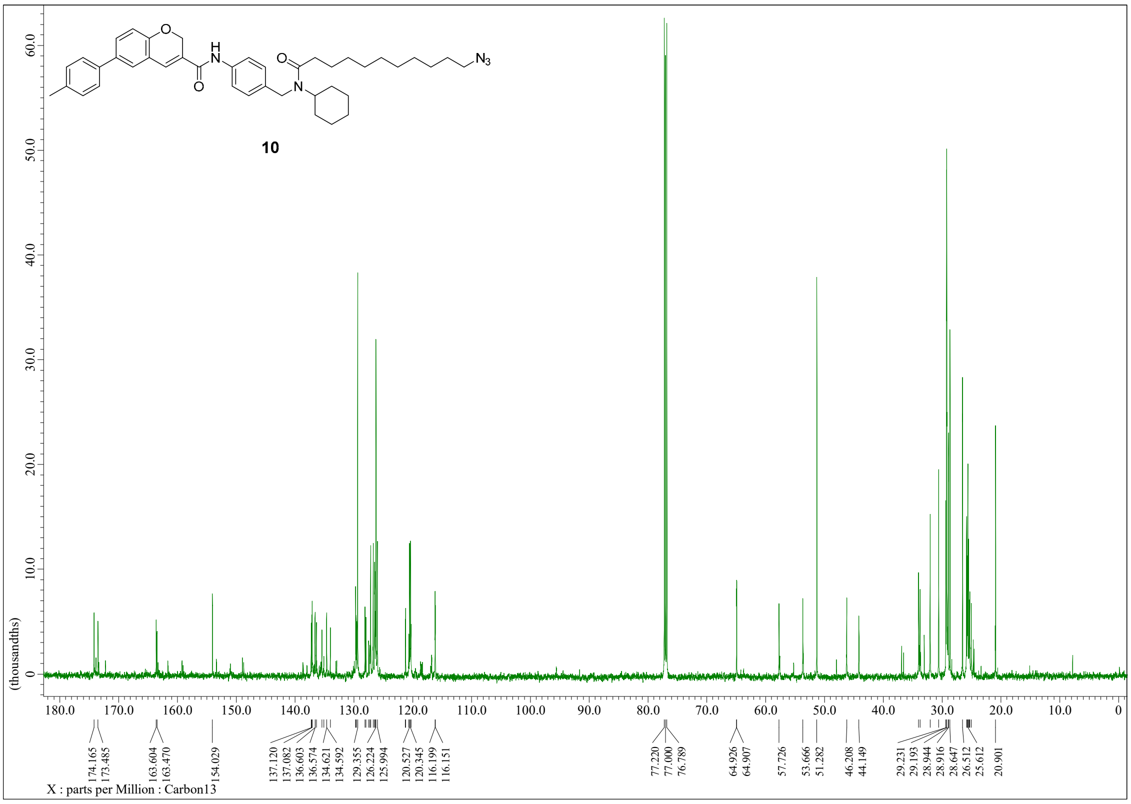

^{1}H-NMR of **10a**

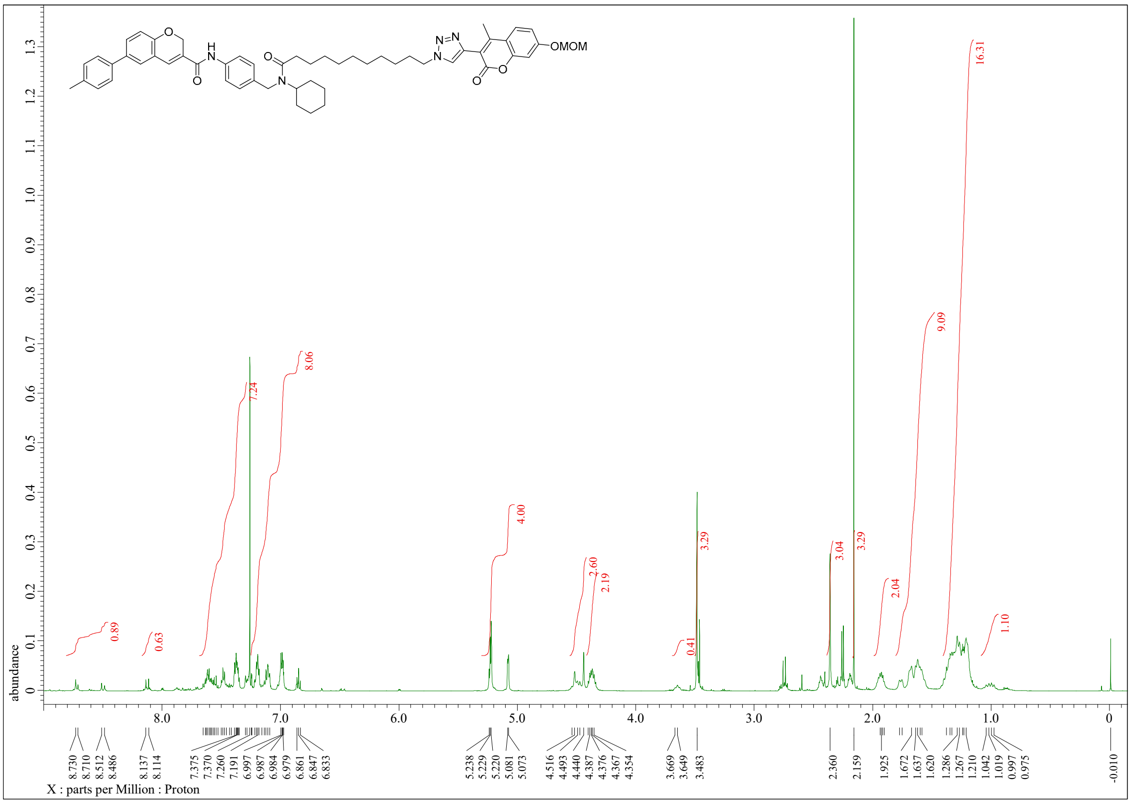

^{13}C-NMR of **10a**

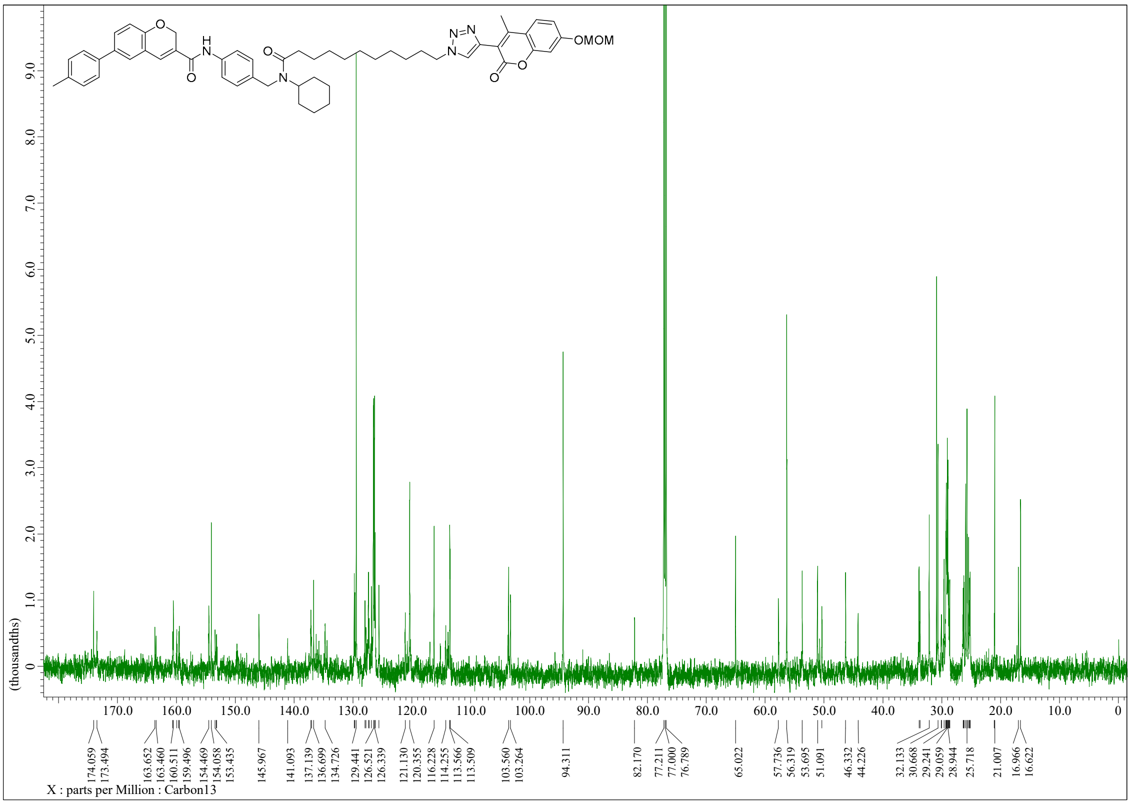

^{1}H-NMR of **2a**

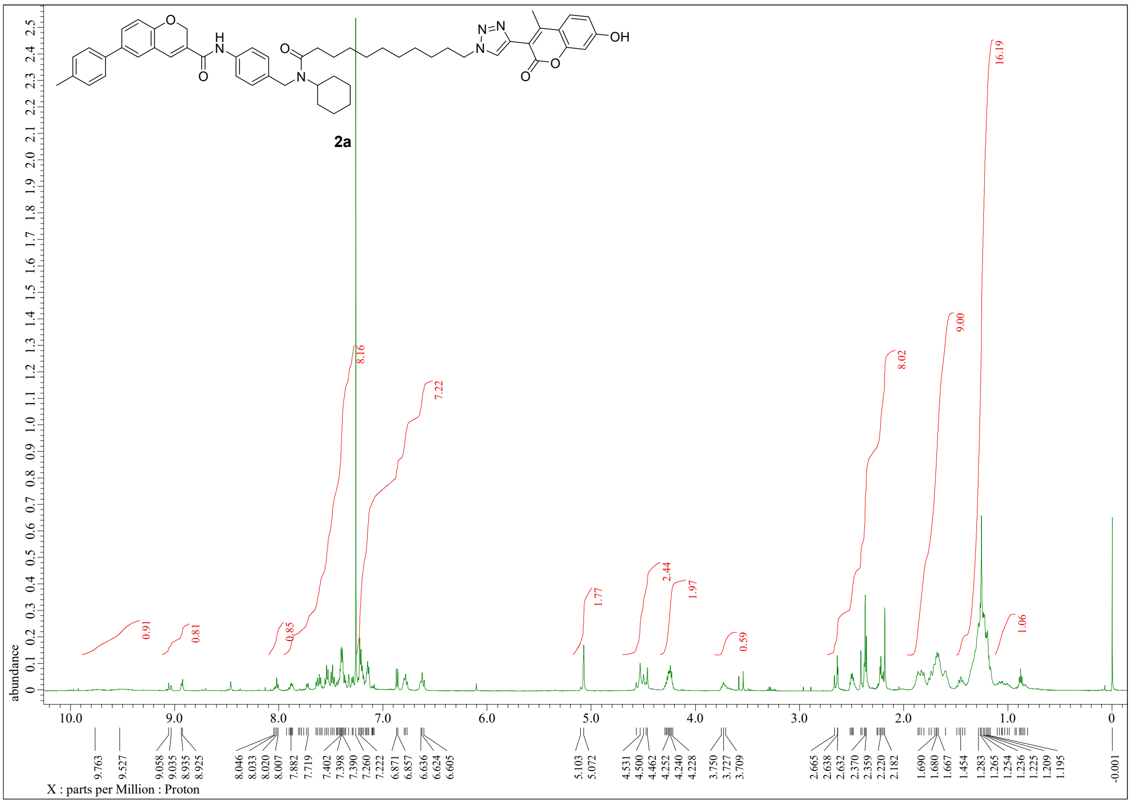

^{13}C-NMR of **2a**

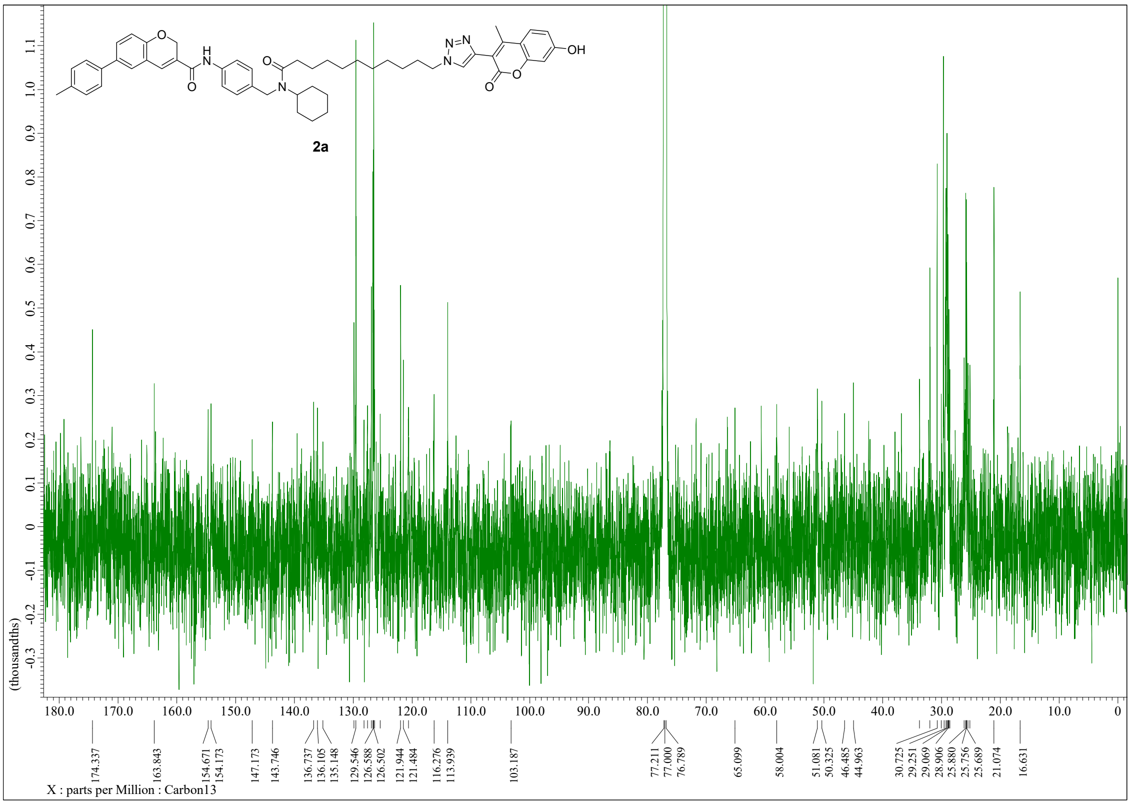

^{1}H-NMR of **3a**

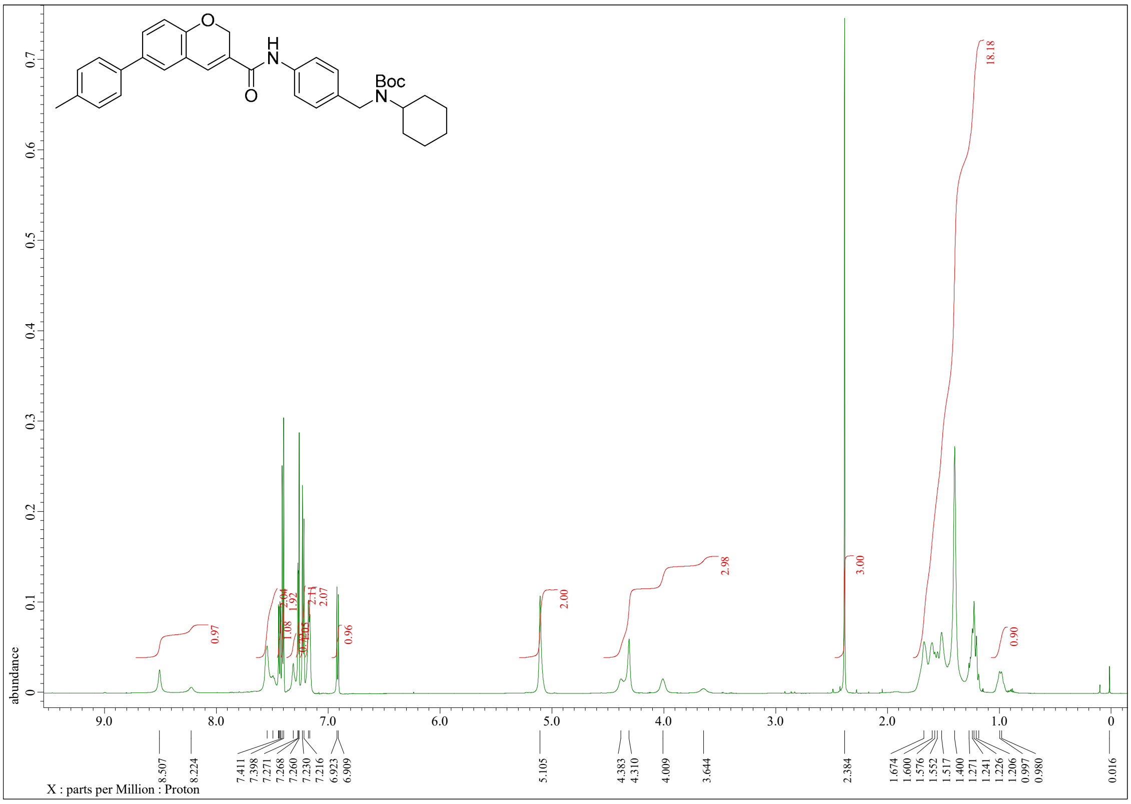

^{13}C-NMR of **3a**

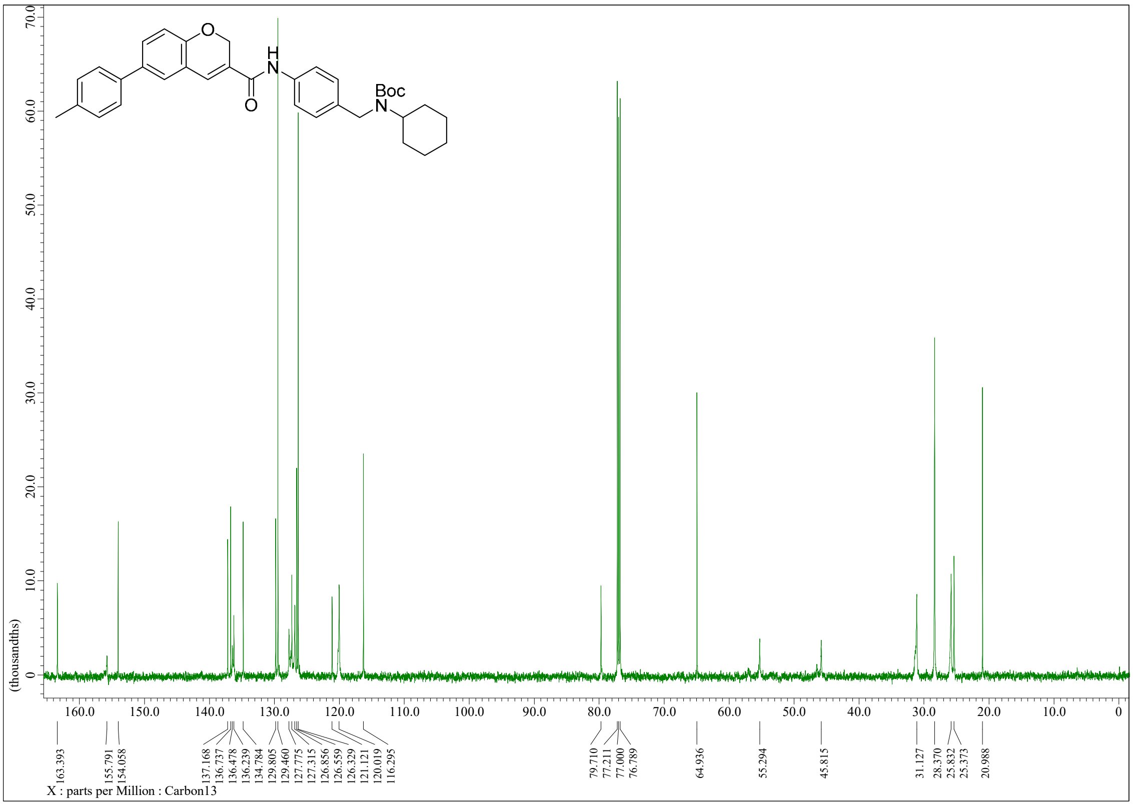

^{1}H-NMR of **11**

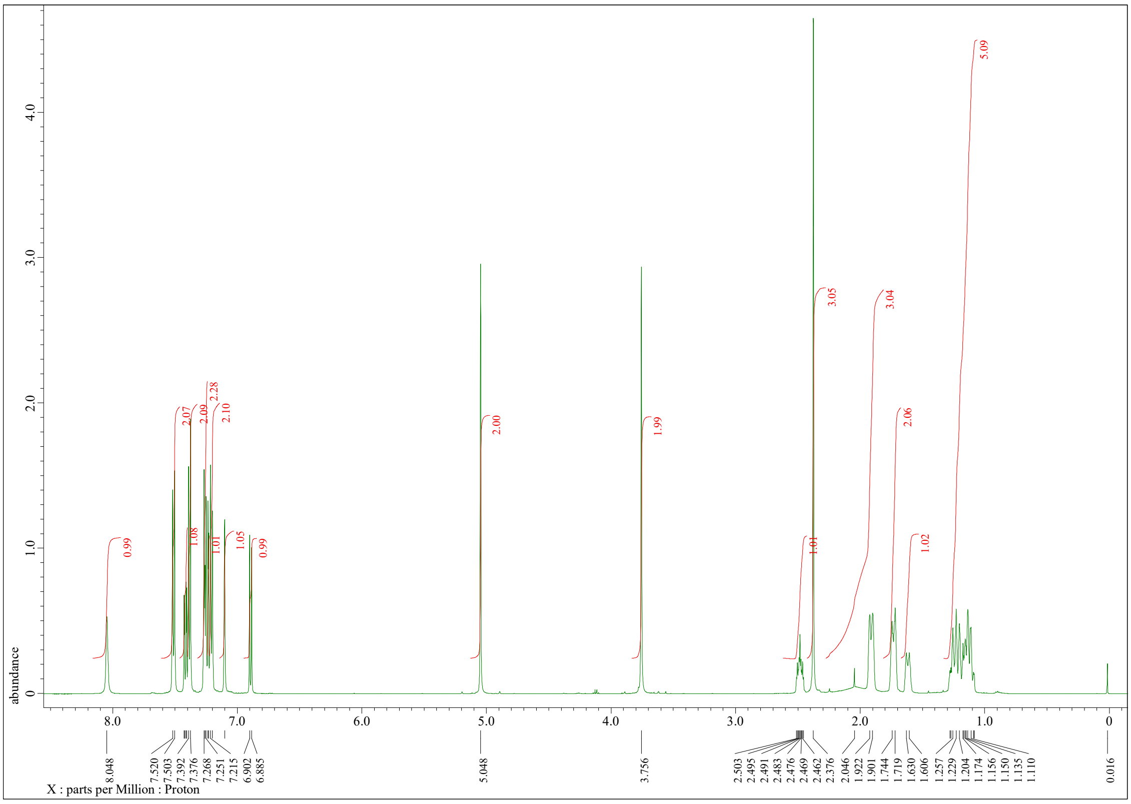

^{13}C-NMR of **11**

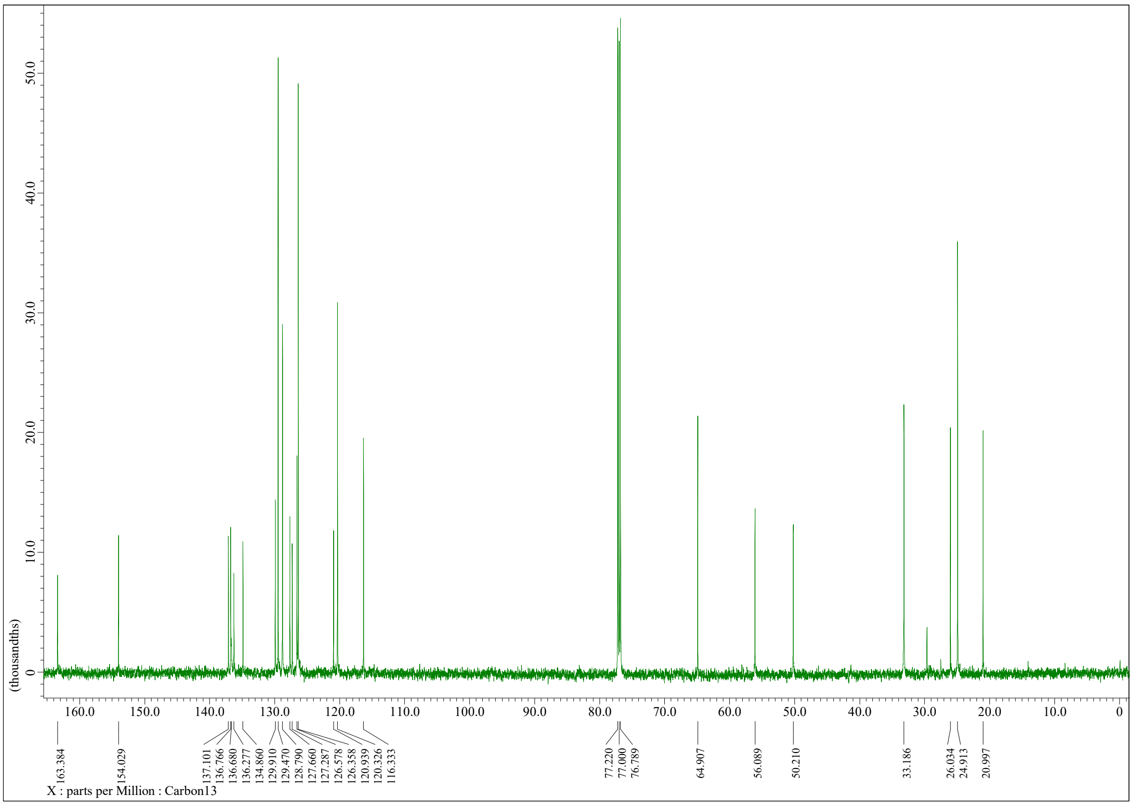

^{1}H-NMR of **11a**

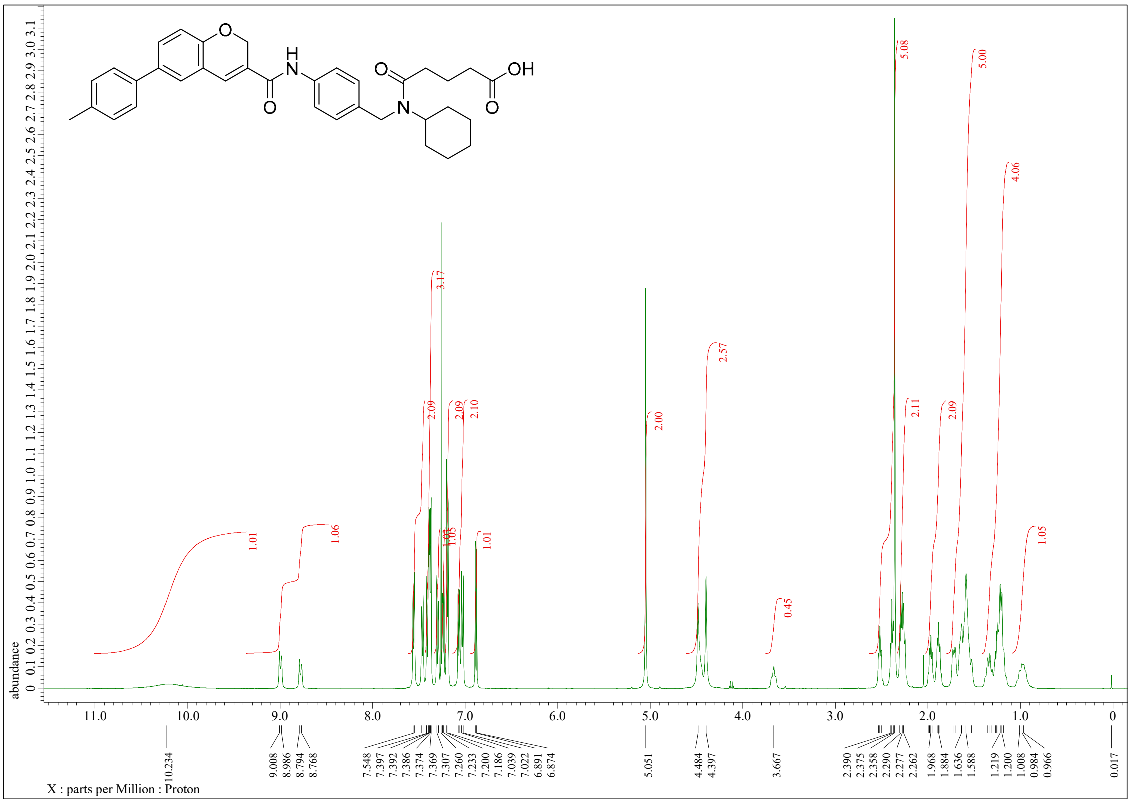

^{13}C-NMR of **11a**

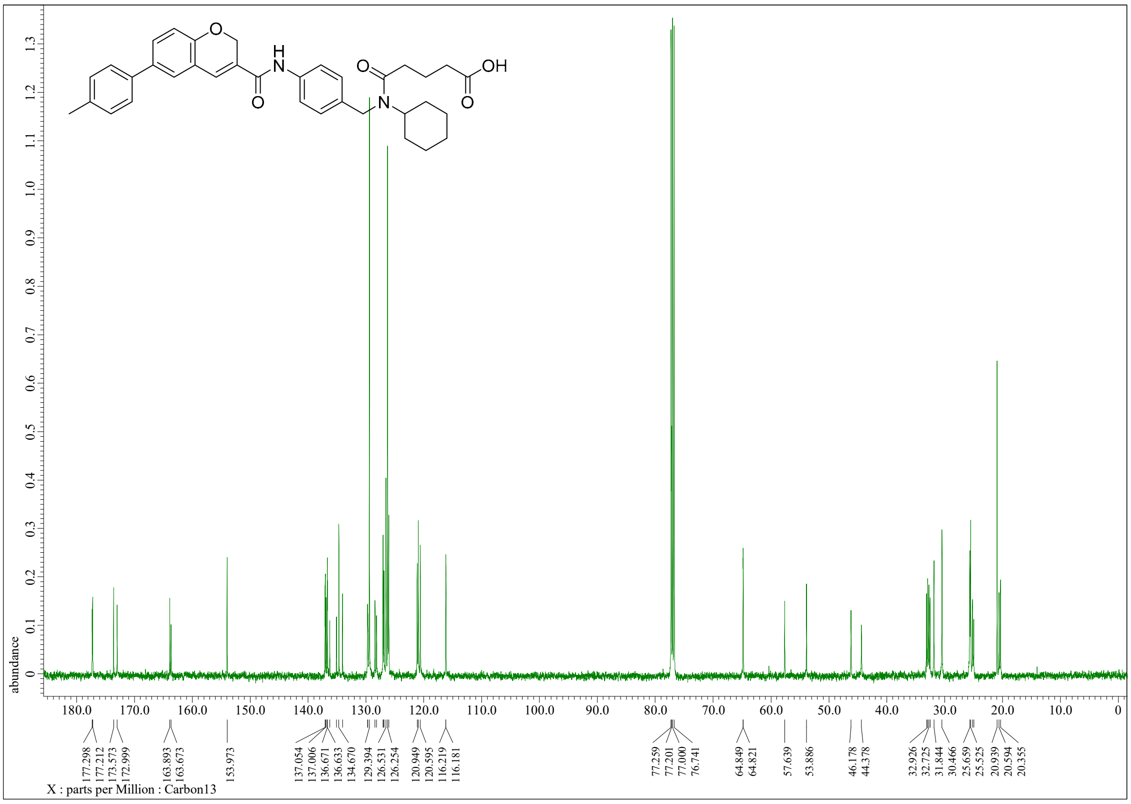

^{1}H-NMR of **12**

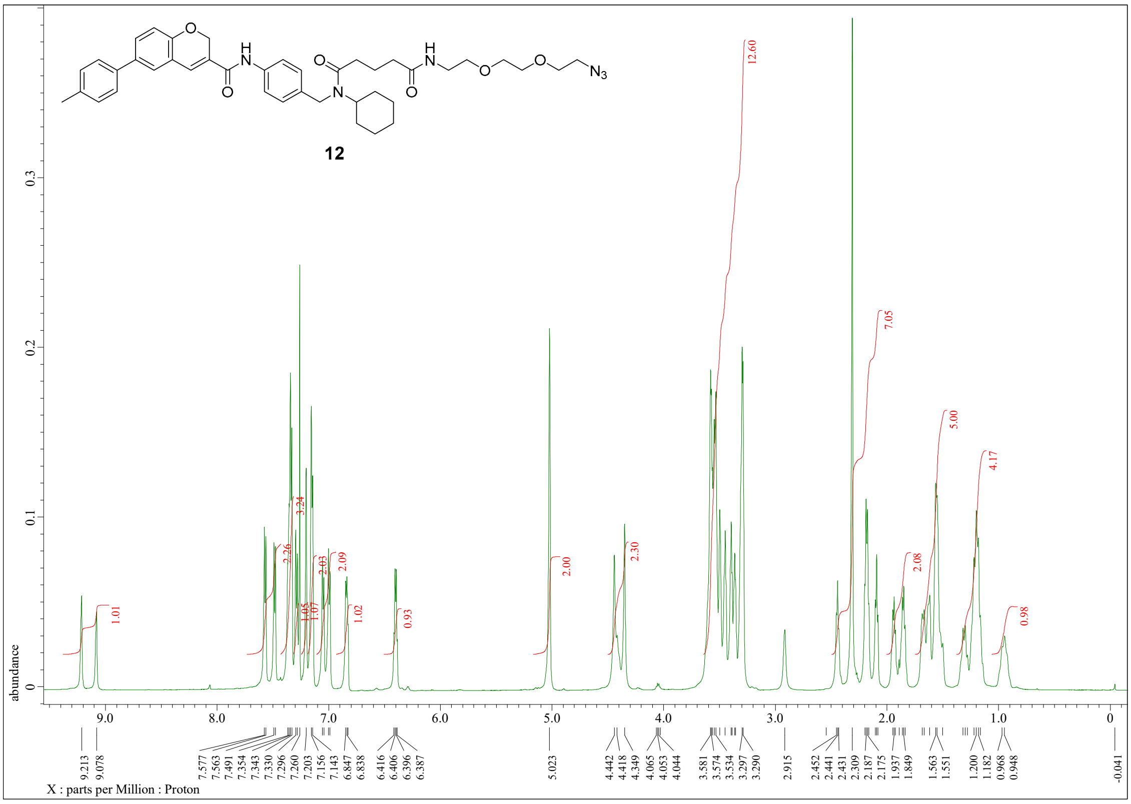

^{13}C-NMR of **12**

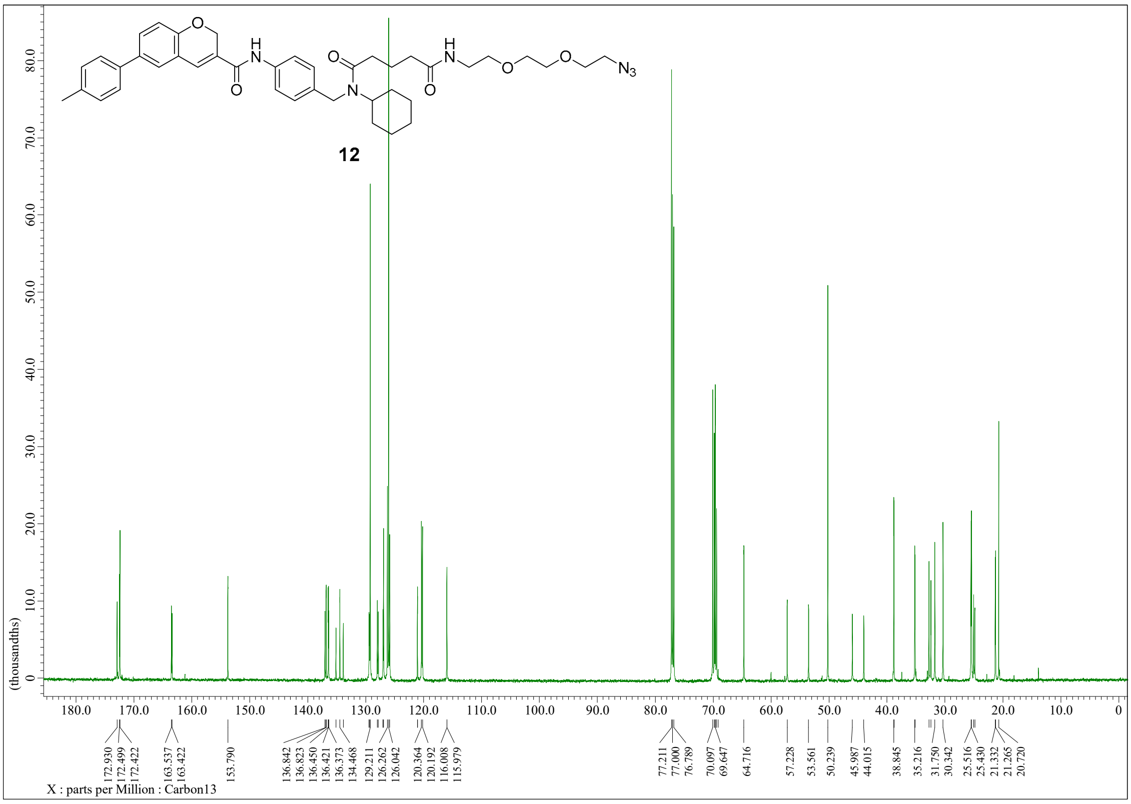

^{1}H-NMR of **12a**

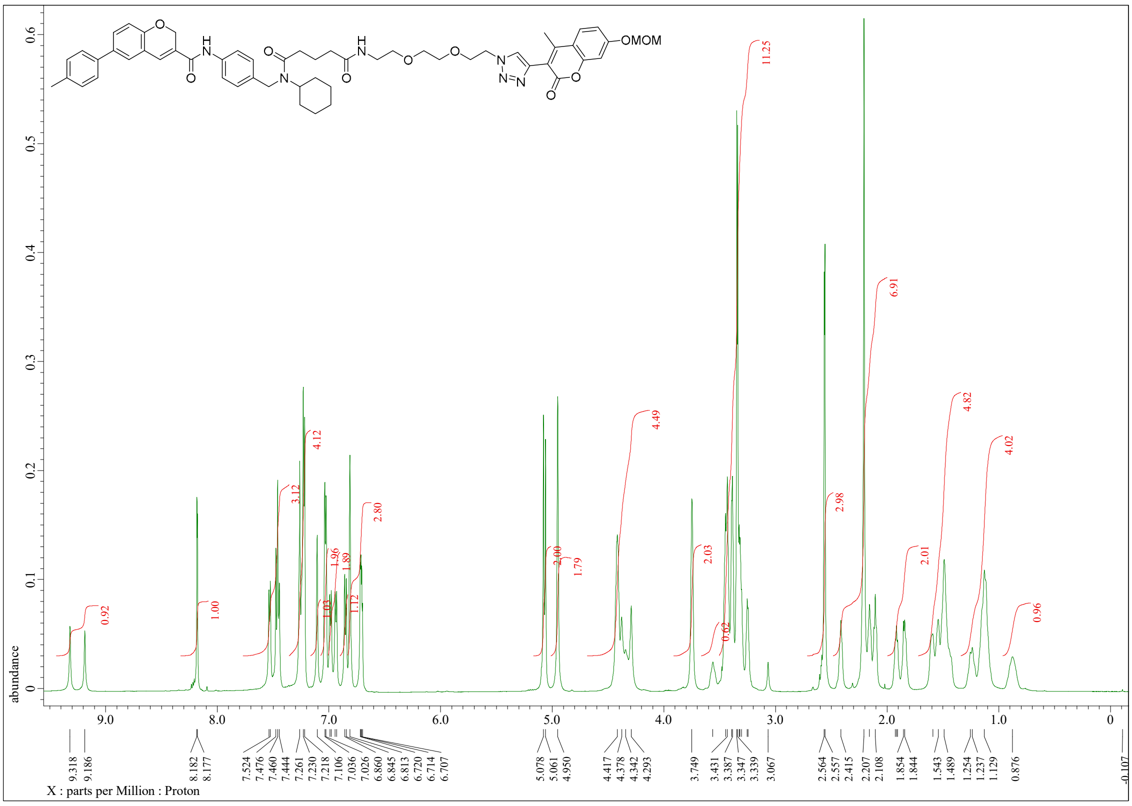

^{13}C-NMR of **12a**

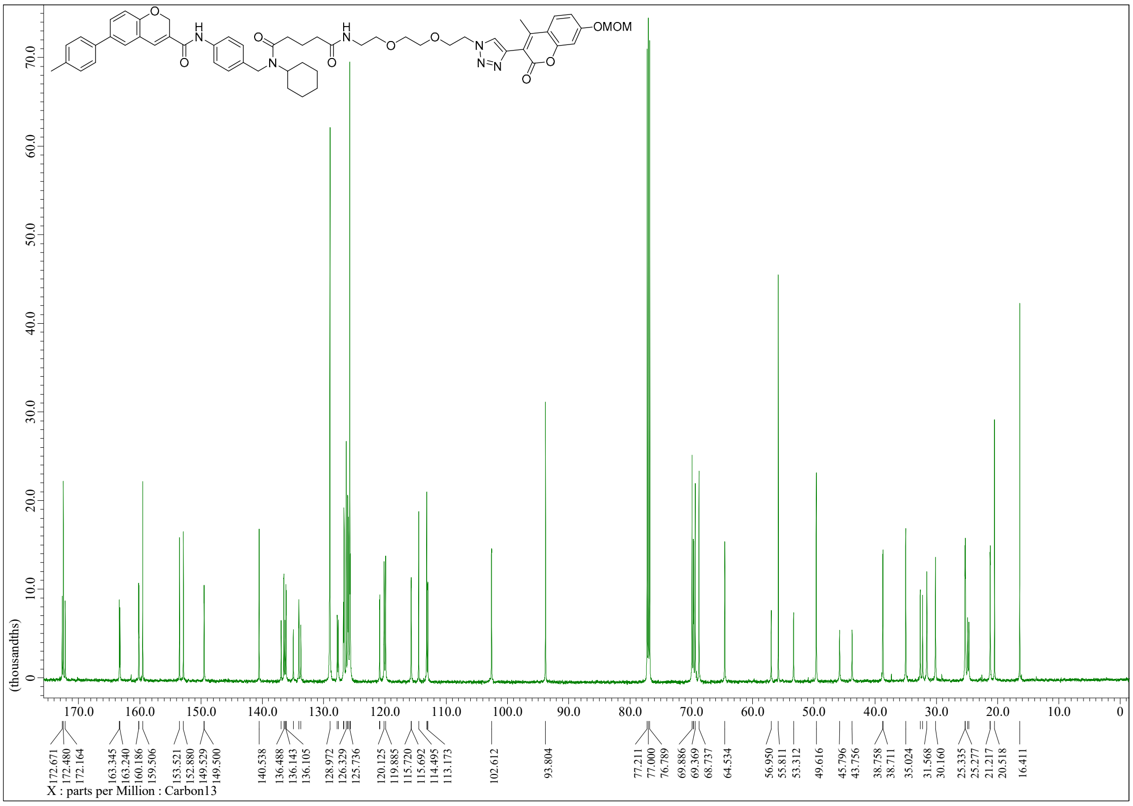

^{1}H-NMR of **2b**

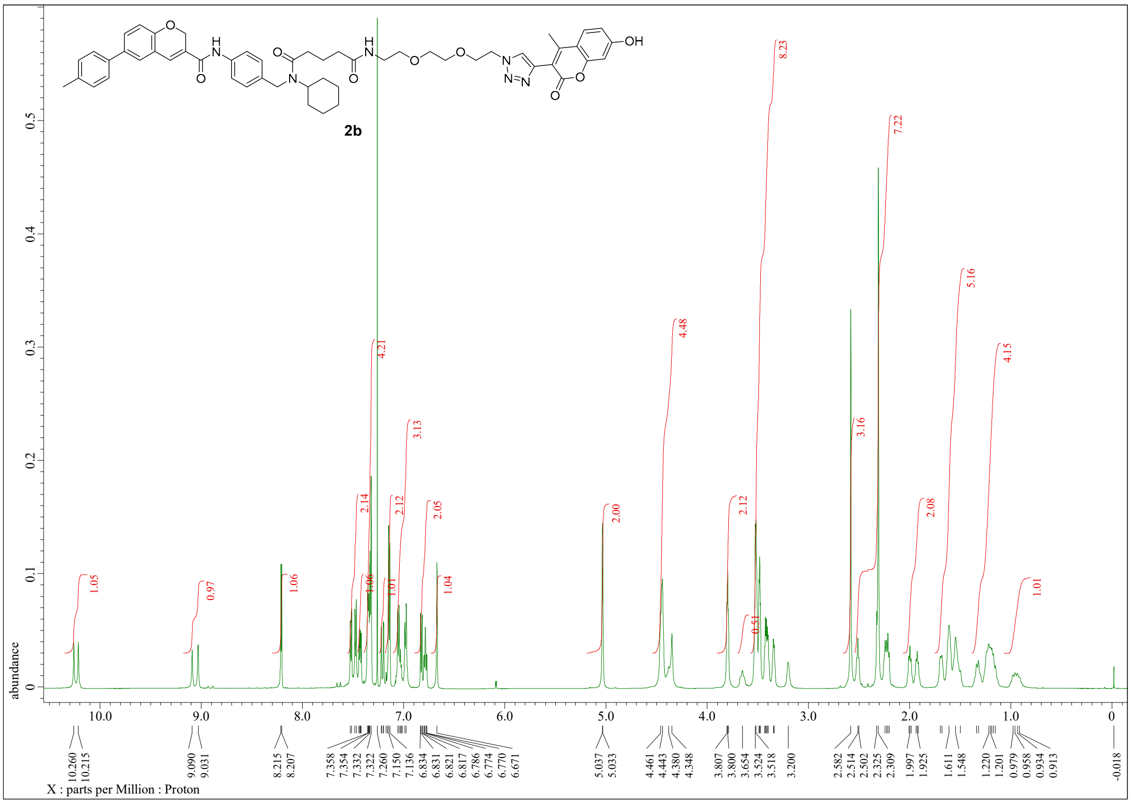

^{13}C-NMR of **2b**

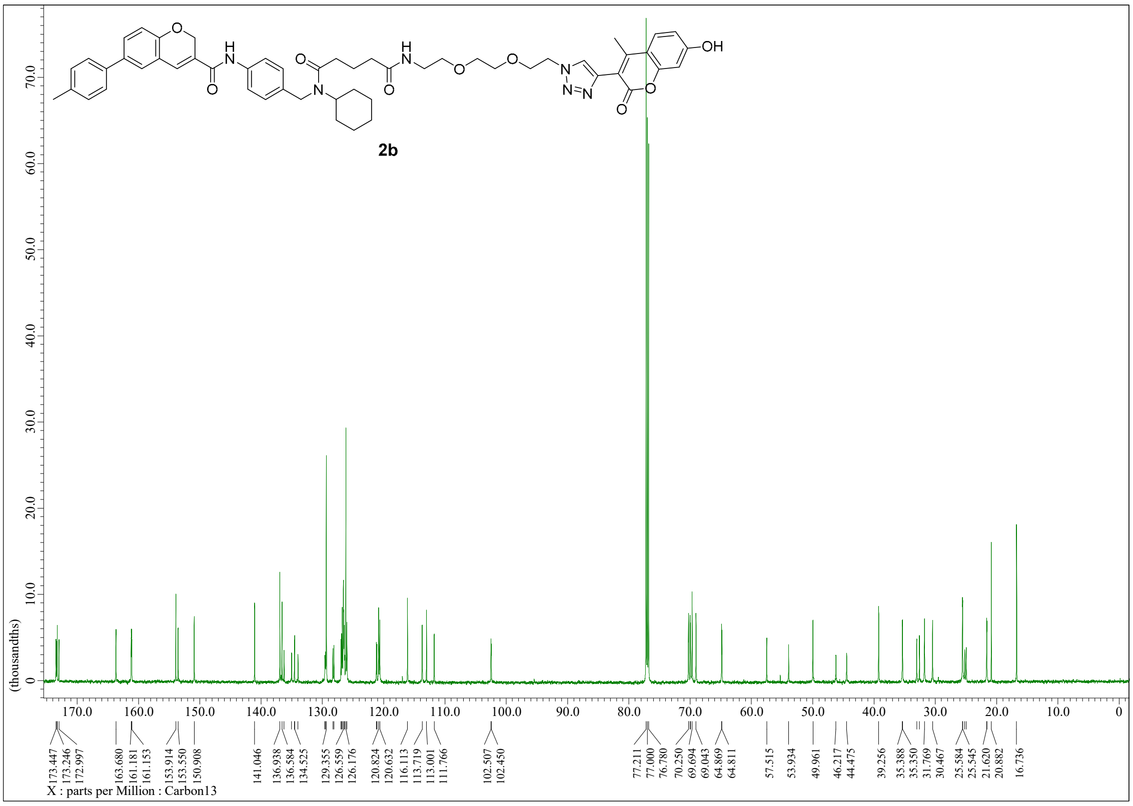

^{1}H-NMR of **13**

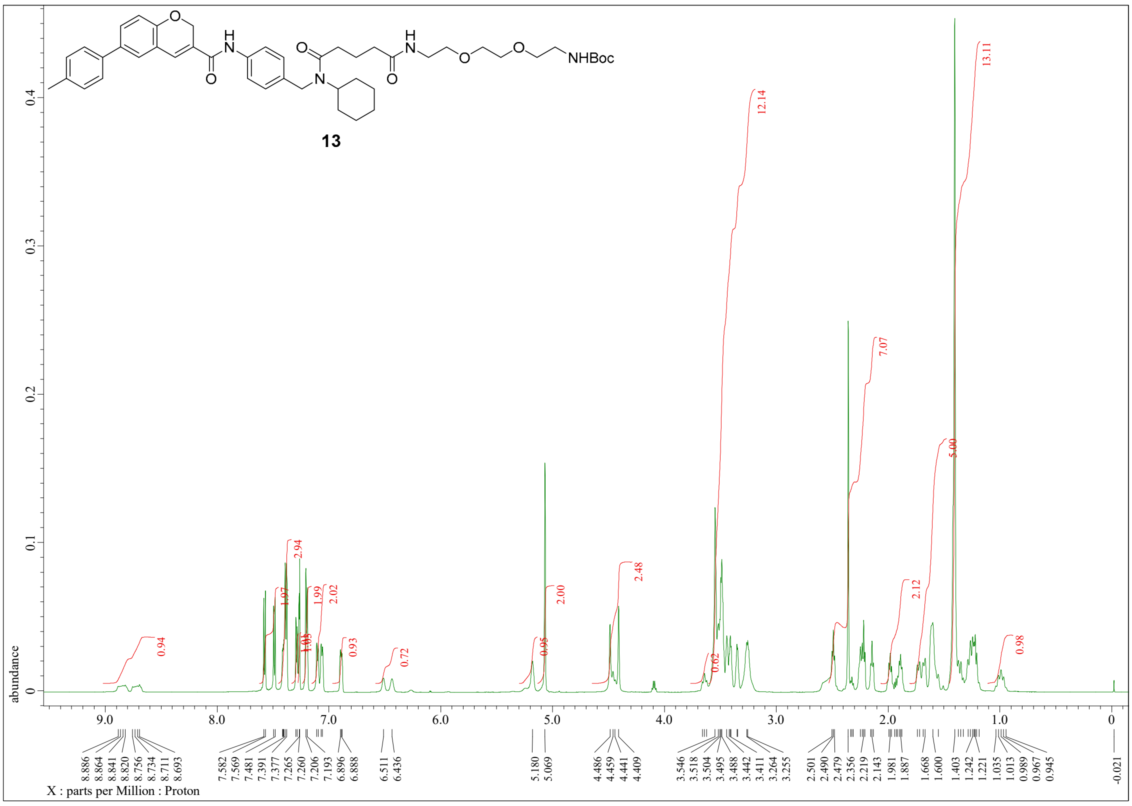

^{13}C-NMR of **13**

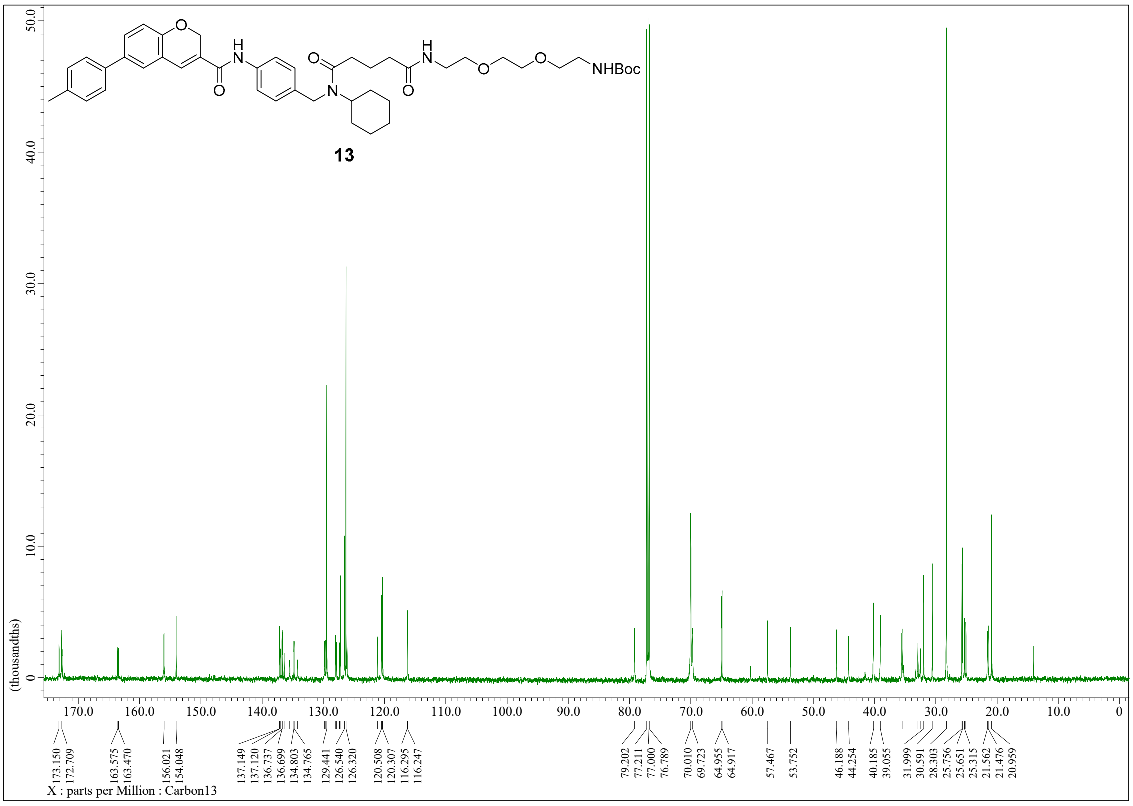

^{1}H-NMR of **2c**

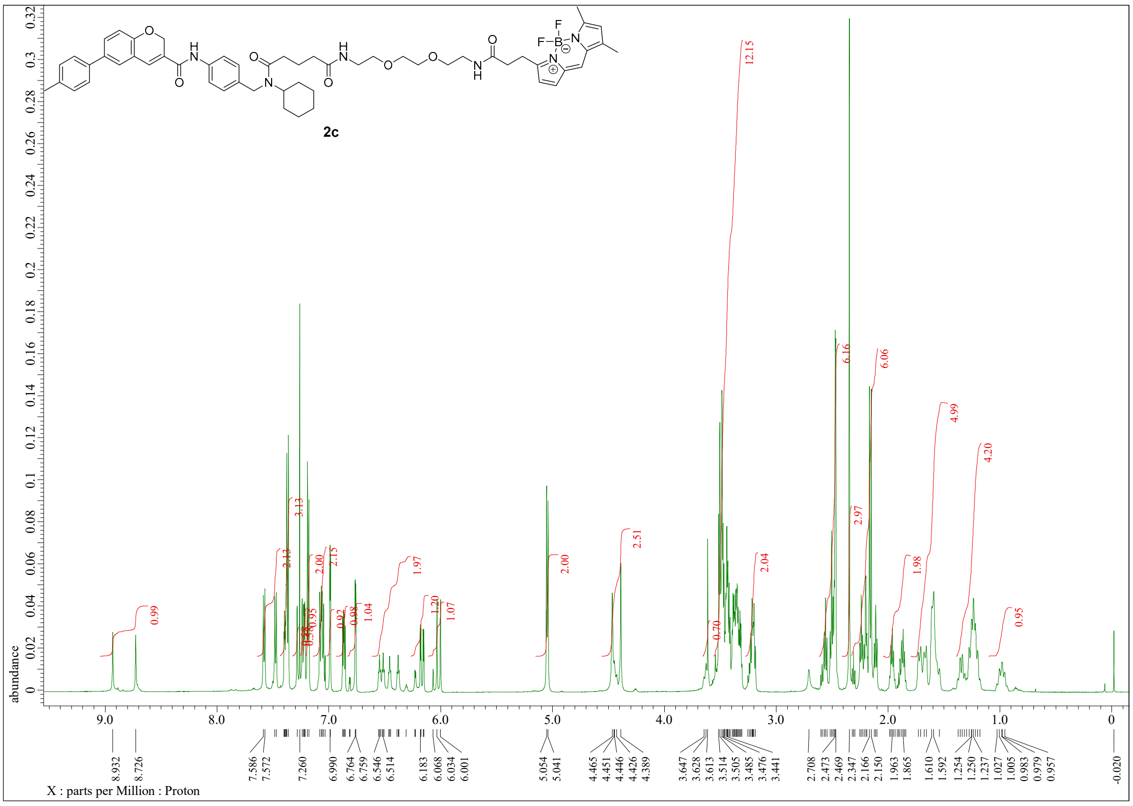

^{13}C-NMR of **2c**

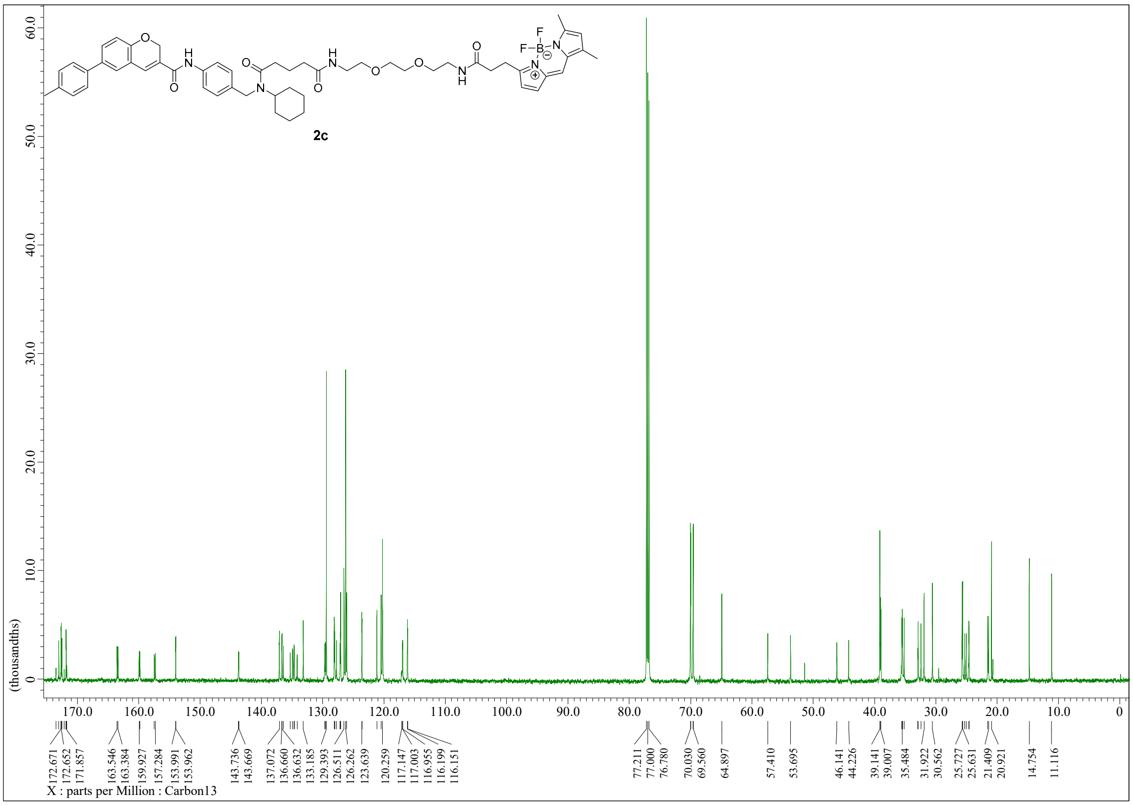

^{1}H-NMR of **14**

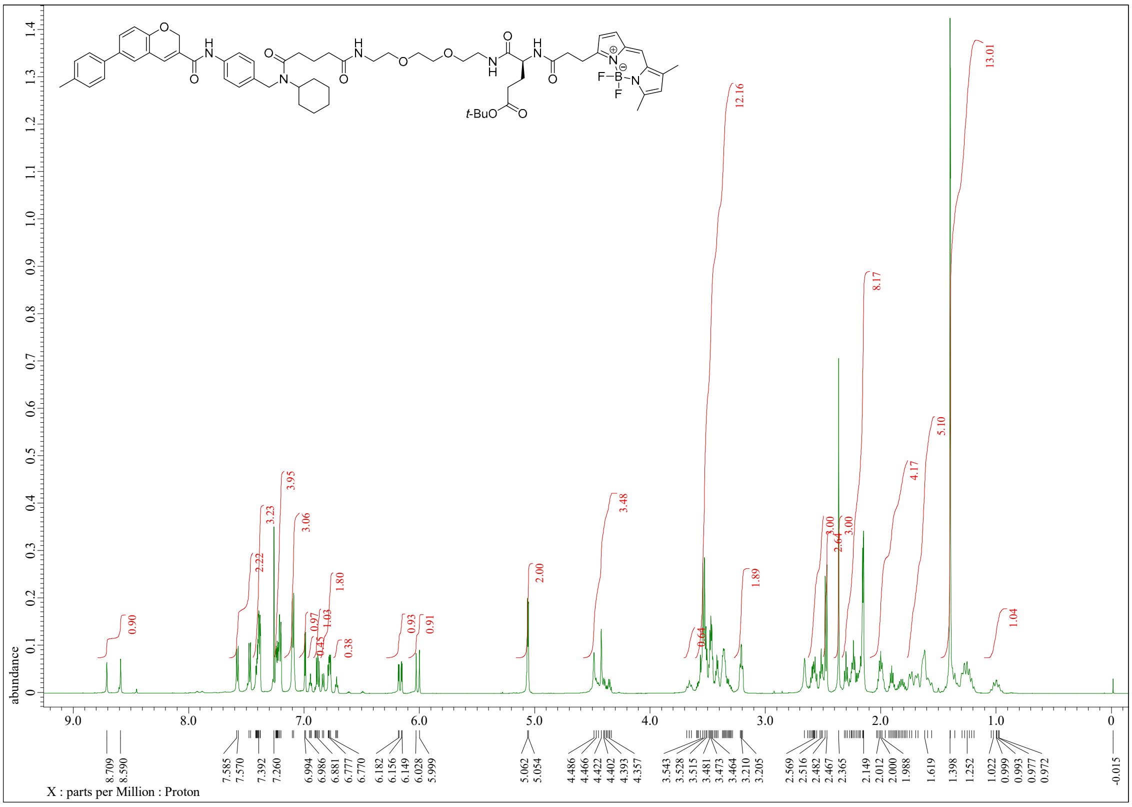

^{13}C-NMR of **14**

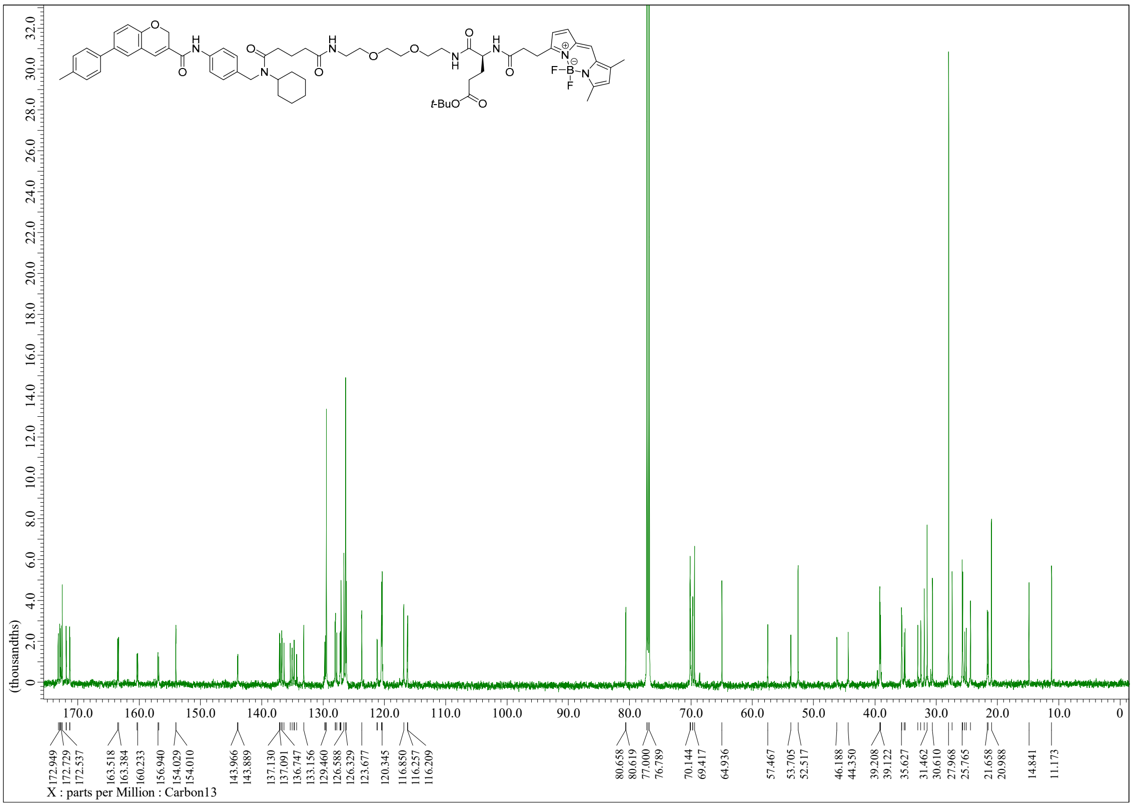

^{1}H-NMR of **2d**

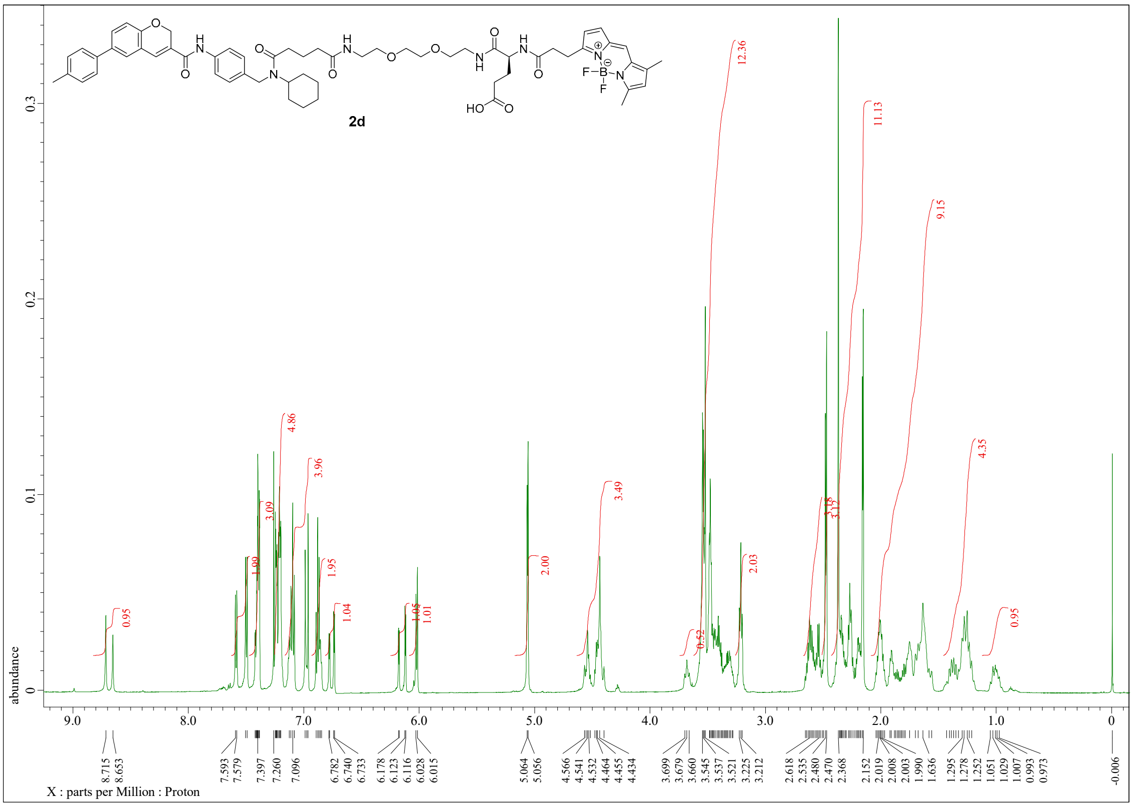

^{13}C-NMR of **2d**

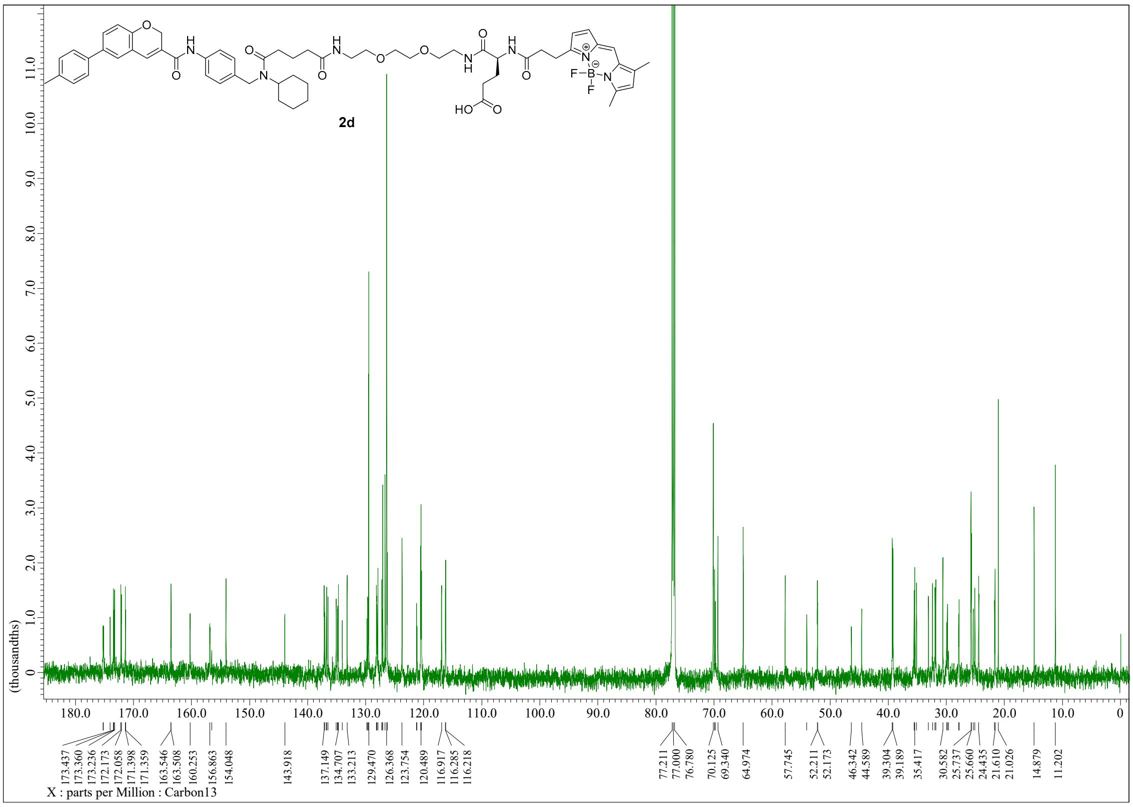

Supplement: Supplementary file 1 [file molecules-30-02655-s001.zip › molecules-3702083-supplementary.pdf]
